# Direct Oral Anticoagulants vs. Vitamin-K Antagonists in the Elderly With Atrial Fibrillation: A Systematic Review Comparing Benefits and Harms Between Observational Studies and Randomized Controlled Trials

**DOI:** 10.3389/fcvm.2020.00132

**Published:** 2020-09-10

**Authors:** Nan-Nan Shen, Yue Wu, Na Wang, Ling-Cong Kong, Chi Zhang, Jia-Liang Wang, Zhi-Chun Gu, Jin Chen

**Affiliations:** ^1^Department of Pharmacy, Affiliated Hospital of Shaoxing University, Shaoxing, China; ^2^Department of Pharmacy, Department of Cardiology, Renji Hospital, School of Medicine, Shanghai Jiao Tong University, Shanghai, China; ^3^Department of Pharmacy, Renmin Hospital, Wuhan University, Wuhan, China; ^4^Department of Pharmacy, The Second Affiliated Hospital of Chongqing Medical University, Chongqing, China; ^5^Department of Evidence-Based Medicine and Clinical Epidemiology, West China Hospital, Sichuan University, Chengdu, China

**Keywords:** stroke, embolism, bleeding, real-world study, warfarin, dabigatran

## Abstract

**Background:** The publication of high-quality observational studies (OSs) has fueled reassessment of the treatment effects of direct oral anticoagulants (DOACs) in the elderly with atrial fibrillation (AF).

**Methods:** The MEDLINE, EMBASE, and Cochrane Library databases were systematically searched (through July 1, 2019) for eligible OSs and randomized controlled trials (RCTs) that reported effectiveness outcomes [stroke or systemic embolism (SE)] or safety outcomes [intracranial hemorrhage (ICH), major bleeding, gastrointestinal bleeding (GIB), myocardial infarction (MI), and all-cause mortality] for DOACs and vitamin-K antagonists (VKAs) in elderly AF patients. A random-effects model was applied to calculate adjusted hazard ratios (HRs) for OSs and relative risks (RRs) for RCTs. Interaction analyses and the ratio of HR (RHR) were used to assess and compare OSs and RCTs.

**Results:** A total of 32 studies involving 547,419 patients were included. No significant difference in treatment effect estimates was found between 27 OSs and 5 RCTs [*P*_*interaction*_ > 0.05 for each and all 95% confidence interval (CI) of RHR crossed 1.0]. Compared with VKAs, DOACs significantly reduced risk for stroke/SE (OSs, HR: 0.87, 95% CI: 0.81–0.94; RCT, RR: 0.82, 95% CI: 0.67–0.96), and ICH (OSs: 0.47 [0.37–0.57]; RCTs: 0.47 [0.31–0.63]), without increasing risk for GIB (OSs: 1.21 [0.98–1.43]; RCTs: 1.34 [0.91–1.77]), and all-cause mortality (OSs: 1.01 [0.92–1.11]; RCTs: 0.94 [0.87–1.00]). Among OSs, DOACs significantly decreased risk for major bleeding (0.87 [0.77–0.98]) and MI (0.89 [0.79–0.99]). It was found that dabigatran, but not other DOACs, significantly increased risk for GIB (1.48 [1.23–1.72]).

**Conclusions:** DOACs were demonstrated to be more effective and safer than VKAs in elderly AF patients, whereas dabigatran users had a 48% increase in risk for GIB.

## Introduction

Atrial fibrillation (AF) prevalence increases with age, from 0.1% at age <35 years to 14% at age >75 years (1, 2). AF patients aged ≥75 years are considered to have an increased risk factor in the stroke risk-stratification tool and contribute 1 point to the CHADS_2_ score and 2 points to the CHA_2_DS_2_-VASc score. Other stroke risk factors, including cardiac failure, hypertension, diabetes mellitus, and stroke history, etc., are also highly prevalent in the elderly, which means that aging is regarded as one of the strongest risk factors for stroke in AF patients (3, 4).

Currently, anticoagulant therapy remains the mainstay for the prevention of stroke in AF. Direct oral anticoagulants (DOACs: dabigatran, rivaroxaban, apixaban, and edoxaban et al.) have favorable practical advantages and efficacy, meaning they represent an alternative to Vitamin K antagonists (VKAs) (5–7). However, no randomized controlled trials (RCT) for elderly populations have been conducted to address the benefits and harms of DOACs, and the results that do exist, mainly from subgroup analyses of RCTs, are not completely credible (8–11). RCT excludes high-risk elderly patients, thus the extrapolation of RCT results in real-world practice is low, due to these differences in patient characteristics.

At present, there is increased awareness that high-quality observational studies (OSs) can support and extend RCT findings to large patient populations in real clinical settings and, as such, may facilitate the validation of conclusions drawn from RCTs. Contemporary OSs (12–19) have addressed these issues concerning elderly AF populations and fueled systematic reassessment of the benefits and harms associated with DOACs. We, therefore, conducted a systematic review and meta-analysis to compare the benefits and harms of DOACs vs. VKAs between high-quality OSs and RCTs among elderly AF patients.

## Methods

This systematic review was established according to the PRISMA Statement and Cochrane Collaboration (20, 21). The protocol for this review was prospectively registered in PROSPERO (CRD42019142881, www.crd.york.ac.uk/PROSPERO/display_record.php?RecordID=142881).

### Data Sources and Searches

Relevant studies were identified by performing English-language searches of MEDLINE, EMBASE, and the Cochrane Library databases (inception to July 1, 2019) using the search strategy outlined in Supplementary Table 1. In addition, relevant articles from reference lists were also searched.

### Study Selection and Outcomes

Studies were eligible for inclusion if they were RCTs or OSs; included elderly patients (≥75 years) with AF; compared DOACs with VKAs (warfarin, phenprocoumon et al.); and reported benefits and harmful outcomes. For the highest quality OSs, only nationwide or health insurance database studies that reported adjusted or matched data using an authorized method to minimize confounding [covariate adjustment (CA), propensity score adjustment (PSA), propensity score matching (PSM), inverse probability of treatment weighting (IPTW)] were included. If multiple OSs from the same data source were identified, the one that reported adjusted data with the longest study period was used. Studies that reported only crude results or were published only in a conference abstract or letter were excluded. Two authors (N-NS and YW) independently reviewed each title and abstract, and assessed full texts of retrieved studies, with any disagreements resolved via consultation with the corresponding authors (Z-CG and JC). The outcomes of this study were stroke or systemic embolism (SE), intracranial hemorrhage (ICH), major bleeding, gastrointestinal bleeding (GIB), myocardial infarction (MI), and all-cause mortality. Major bleeding was defined as a decrease in hemoglobin level of 2 g/dL or greater within a 24-h period, or leading to a transfusion of two or more units of packed red cells, or requiring an additional endoscopy intervention, according to International Society on Thrombosis and Hemostasis (ISTH) criteria (22).

### Data Extraction and Quality Assessment

Two authors (N-NS and YW) independently extracted the data using a priori designed form, which included study characteristics, patient demographics and clinical characteristics of the included studies, and data on clinical outcomes (occurrence number and the total number for RCTs; adjusted HR for OSs). If appropriate, data from DOACs-naïve patients was extracted because the use of data from VKAs-switchers may lead to the overestimation of bleeding risk of DOACs vs. VKAs. The methodological quality of each included RCT was assessed according to the Cochrane Collaboration Risk of Bias Tool (23). Considering an inherently higher bias risk of OSs relative to RCTs, the methodological quality of each OS was evaluated using the following items: (1) using authorized adjustment method to deal with selection bias; (2) potential for residual confounding; (3) using methods to handle time-varying covariates and information censoring; and (4) reporting baseline characteristics and outcome measures in detail (24). Ratings of low, moderate, or high risk bias were assigned to each item.

### Data Synthesis and Statistical Analysis

To compare the benefits and harms of DOACs vs. warfarin, a random-effects meta-analysis was performed to synthesize the data of OSs [adjusted hazard ratios (HRs) and 95% confidence intervals (95%CI)] and RCTs [relative risks (RRs) and 95%CI], respectively, with an *I*^2^-test >50% representing considerable heterogeneity (25). Afterward, to evaluate the comparability between OSs and RCTs, interaction analysis (*P* for interaction) was applied and the ratio of HRs (RHR), which were calculated by dividing the OS summary HR by the RCT summary RR (26). 95% CIs of RHR were calculated by summing the estimate variances of OSs and RCTs. An RHR <1 indicated a greater treatment effect estimate of OSs compared to RCTs, while an RHR >1 implied a lower estimate of OSs vs. RCTs (27). In addition, subgroup analyses of OSs were performed based on individual agents (dabigatran, rivaroxaban, apixaban, and edoxaban), gender (men and women), age (>80, >85, and >90 years), and population (U.S.A, Canada, Italy, Germany, Sweden, Danish, France, Spain, Korea, etc.). To detect the robustness of the results, a sensitivity analysis was conducted by sequential elimination of each study from the pool. Meta-regression analysis was performed to determine the potential bias of effect factors on outcomes. Publication bias was evaluated by funnel plots and quantitative analysis of Begg's test and Egger's test (20). Statistics were performed using STATA software (version13, StataCorp, College Station, Texas, USA), with *P* < 0.05 indicating a statistically significant difference.

## Results

### Study Selection and Study Characteristics

**Figure 1:**
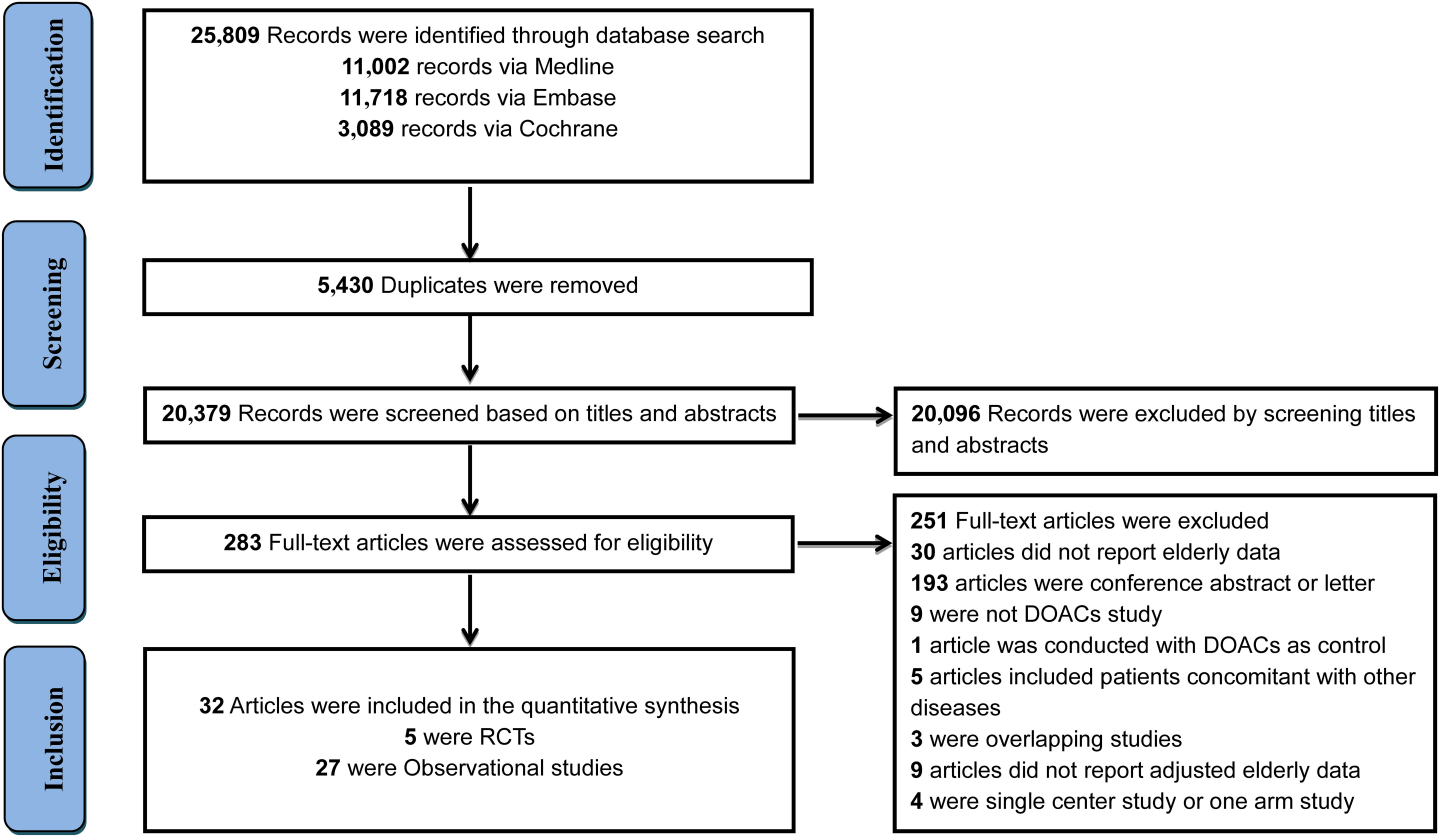
Flow diagram for the selection of eligible studies. DOACs, direct oral anticoagulants; RCTs, randomized controlled trials.

In total, 25,809 records were initially identified through a comprehensive search, with 5,430 duplicates removed and 20,096 records excluded after screening the titles and abstracts of studies. The remaining 283 full-text articles were reviewed and 251 articles were excluded for reasons presented in Figure 1 and Supplementary Table 2. Finally, 32 studies that involved 547,419 elderly participants and evaluated DOACs or VKAs were included, with 27 OSs (519,267 patients) and 5 RCTs (28,152 patients). Twenty-seven OSs were conducted in 10 countries or regions, with most studies occurring in the U.S.A (*n* = 11). Only two studies were specifically designed to investigate clinical outcomes in the elderly, the remaining studies presented interesting data from subgroup analyses. Dabigatran use was involved in 21 studies, rivaroxaban in 12, apixaban in 9, and edoxaban in one study. All OSs reported the detailed adjustment method: 14 studies used PSM, 7 applied PSA, 5 employed IPTW, and one used CA. The follow-up duration ranged widely from 60 days to 2.06 years (Supplementary Table 3). Patient and clinical characteristics, bleeding history, and concomitant drugs are summarized in Supplementary Tables 4, 5. Five RCTs reported data according to age subgroup (aged >75 or <75 years) also fulfilled inclusion criteria. The percent of patients aged >75 ranged from 31.3 to 43.3% and follow-up periods ranged from 1.8 to 2.8 years among included RCTs (Supplementary Table 6). The Rocket-AF trial (rivaroxaban) recruited subjects at the highest CHADS_2_ score of 3.5 (Supplementary Table 7).

### Risk of Bias

Of all included Oss studies, selection bias and selective reporting were reported using low-risk methods, and no high-risk bias items were found (Supplementary Table 8). The included RCTs met all bias tool items except the ROCKET AF trial, which was not blinded for participants and personnel (Supplementary Table 9). Overall, the included OSs and RCTs were of modest to high quality.

### Comparison of Benefits and Harms Between OSs and RCTs

**Figure 2:**
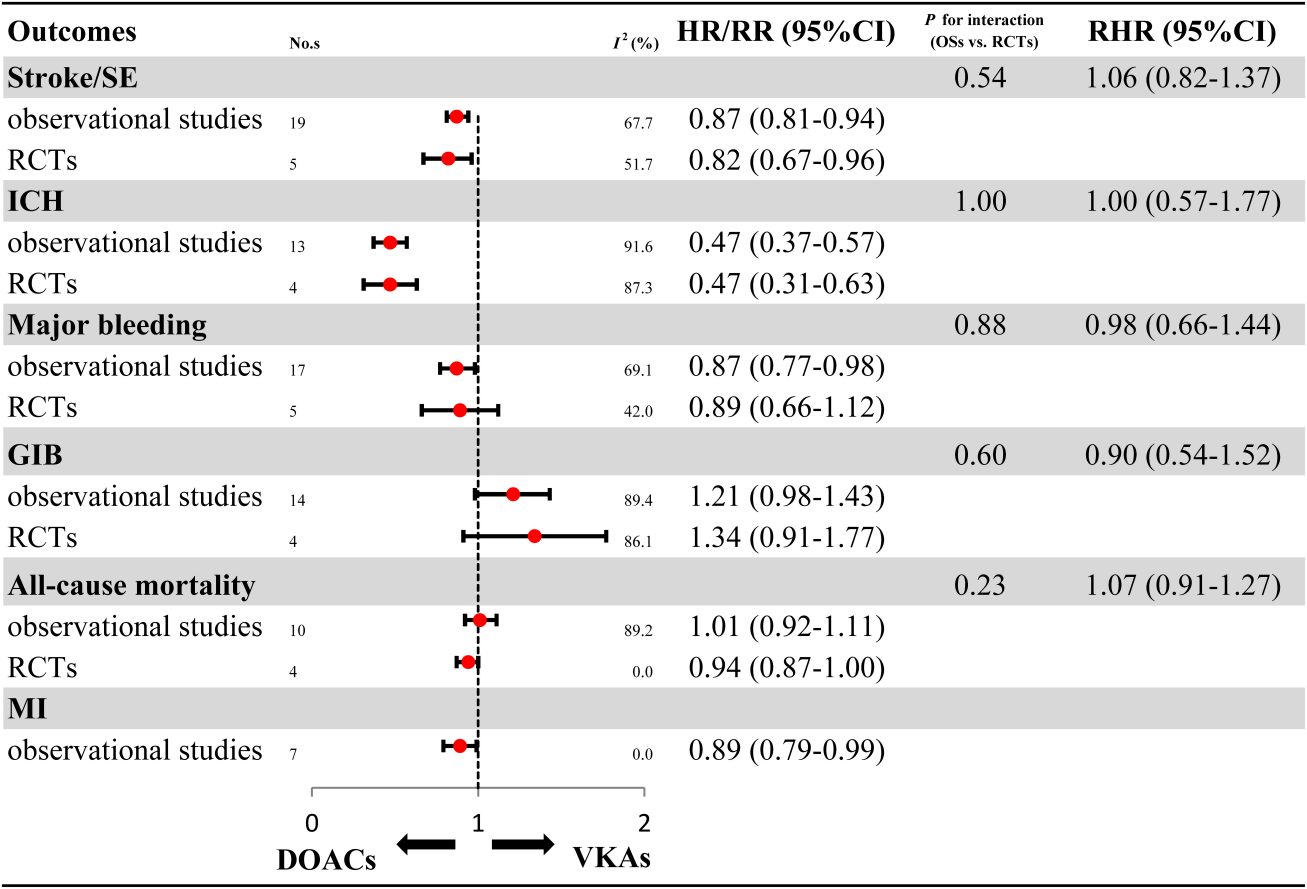
Effectiveness and safety of DOACs in OSs and RCTs. RCTs, randomized controlled trials; Oss, observational studies; RR, relative risk; HR, hazard ratio; 95%CI, 95% confidence interval; SE, systemic embolism; ICH, intracranial hemorrhage; GIB, gastrointestinal bleeding; MI, myocardial infarction; DOACs, direct oral anticoagulants; VKAs, vitamin-K antagonists; No.s, number of included studies.

Overall analyses on the benefits and harms of DOACs in elderly patients with AF are presented in Figure 2 and Supplementary Figures 1–11. For OSs, compared with VKAs, DOACs significantly reduced risks for stroke/SE (HR: 0.87, 95%CI: 0.81–0.94, *I*^2^: 67.7%), ICH (HR: 0.47, 95%CI: 0.37–0.57, *I*^2^: 69.1%), major bleeding (HR: 0.87, 95%CI: 0.77–0.98, *I*^2^: 91.6%), and MI (HR: 0.89, 95%CI: 0.79–0.99, *I*^2^: 0.0%). Meanwhile, DOACs had no significant effect on GIB (HR: 1.21, 95%CI: 0.98–1.43, *I*^2^: 89.4%) and all-cause mortality (HR: 1.01, 95%CI: 0.92–1.11, *I*^2^: 89.2%). For RCTs, in comparison to VKAs, DOACs significantly reduced risks for stroke/SE (RR: 0.82, 95%CI: 0.67–0.96, *I*^2^: 51.7%) and ICH (RR: 0.47, 95%CI: 0.31–0.63, *I*^2^: 42.0%), and had no clear effect on major bleeding (RR: 0.89, 95%CI: 0.66–1.12, *I*^2^: 87.3%), GIB (RR: 1.34, 95%CI: 0.91–1.77, *I*^2^: 86.1%), and all-cause mortality (RR: 0.94, 95%CI: 0.87–1.00, *I*^2^: 0.0%). None of the included RCTs studies reported DOACs data on MI. The comparability on treatment effect estimates did not find a significant difference between OSs and RCTs (*P*_*interaction*_: 0.54, RHR: 1.06, 95%CI: 0.82–1.37 for stroke/SE; *P*_*interaction*_: 1.00, RHR: 1.00, 95%CI: 0.57–1.77 for ICH; *P*_*interaction*_: 0.88, RHR: 0.98, 95%CI: 0.66–1.44 for major bleeding; *P*_*interaction*_: 0.60, RHR: 0.90, 95%CI: 0.54–1.52 for GIB; *P*_*interaction*_: 0.23, RHR: 1.07, 95%CI: 0.91–1.27 for all-cause mortality, respectively).

### Comparison of Benefits and Harms of Individual DOACs in OSs

**Figure 3:**
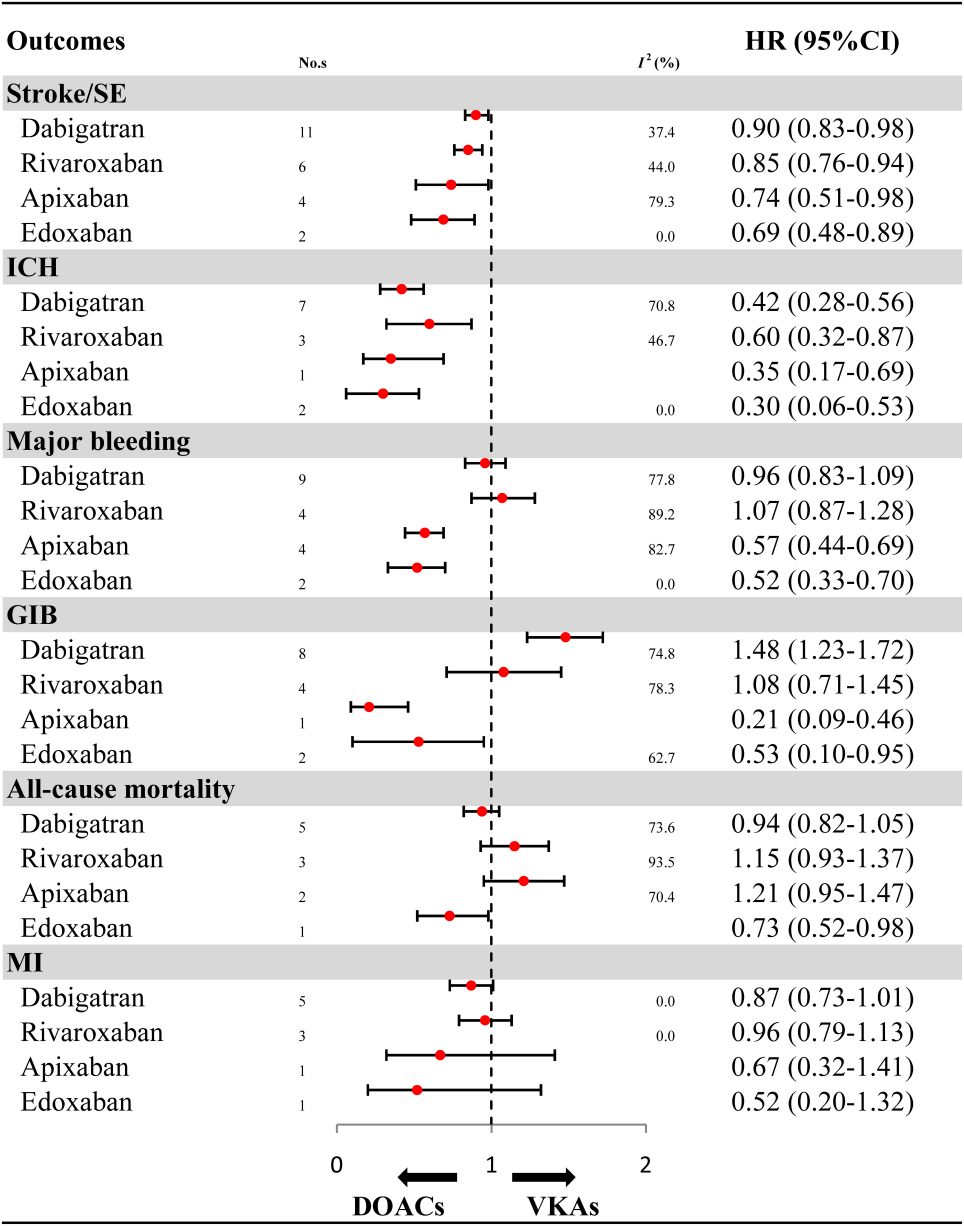
Effectiveness and safety of DOACs by individuals. HR, hazard ratio; 95%CI, 95% confidence interval; SE, systemic embolism; ICH, intracranial hemorrhage; GIB, gastrointestinal bleeding; MI, myocardial infarction; DOACs, direct oral anticoagulants; VKAs, vitamin-K antagonists; No. s, number of included studies.

For OSs, further analyses on the individual DOACs (dabigatran, rivaroxaban, apixaban, and edoxaban) are summarized in Figure 3 and Supplementary Figures 12–17. Compared with VKAs, dabigatran (HR: 0.90, 95%CI: 0.83–0.98, *I*^2^: 37.4%), rivaroxaban (HR: 0.85, 95%CI: 0.76–0.94, *I*^2^: 44.0%), apixaban (HR: 0.74, 95%CI: 0.51–0.98, *I*^2^: 79.3%), and edoxaban (HR: 0.69, 95%CI: 0.48–0.89, *I*^2^: 0.0%) were all associated with reduced risk for stroke/SE. Similarly, the decreased risk for ICH was found in dabigatran (HR: 0.42, 95%CI: 0.28–0.56, *I*^2^: 70.8%), rivaroxaban (HR: 0.60, 95%CI: 0.32–0.87, *I*^2^: 46.7%), apixaban (HR: 0.35, 95%CI: 0.17–0.69), and edoxaban (HR: 0.30, 95%CI: 0.06–0.53, *I*^2^: 0.0%). Compared with VKAs, apixaban (HR: 0.57, 95%CI: 0.44–0.69, *I*^2^: 82.7%) and edoxaban (HR: 0.52, 95%CI: 0.33–0.70, *I*^2^: 0.0%), but not dabigatran (HR: 0.96, 95%CI: 0.83–1.09, *I*^2^: 77.8%) and rivaroxaban (HR: 1.07, 95%CI: 0.87–1.28, *I*^2^: 89.2%), significantly reduced risk for major bleeding. Likewise, apixaban (HR: 0.21, 95%CI: 0.09–0.46) and edoxaban (HR: 0.53, 95%CI: 0.10–0.95, *I*^2^: 62.7%), but not rivaroxaban (HR: 1.08, 95%CI: 0.71–1.45, *I*^2^: 78.3%), significantly reduced risk for GIB when compared to VKAs. It is worth noting that dabigatran significantly increased risk for GIB (HR: 1.48, 95%CI: 1.23–1.72, *I*^2^: 74.8%). Edoxaban, but not other DOACs, significantly reduced risk for all-cause mortality (HR: 0.73, 95%CI: 0.52–0.98). No significant effect on MI was found in all DOACs (HR: 0.87, 95%CI: 0.73–1.01, *I*^2^: 0.00% for dabigatran; HR: 0.96, 95%CI: 0.79–1.13, *I*^2^: 0.00% for rivaroxaban; HR: 0.67, 95%CI: 0.32–1.41 for apixaban; HR: 0.52, 95%CI: 0.20–1.32 for edoxaban).

### Subgroup Analyses Based on Gender, Age, and Population in OSs

**Figure 4:**
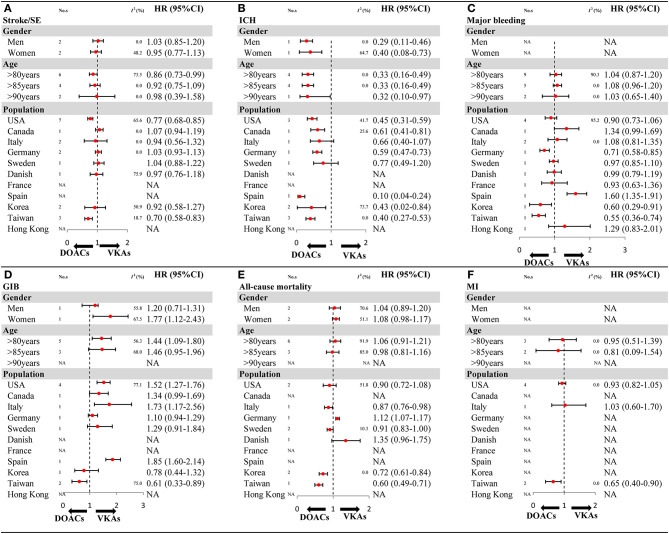
The key subgroup analyses for OSs according to gender, age, and population **(A)** stroke/SE; **(B)** ICH; **(C)** major bleeding; **(D)** GIB; **(E)** all-cause mortality; **(F)** MI. OSs, observational studies; HR, hazard ratio; 95%CI, 95% confidence interval; SE, systemic embolism; ICH, intracranial hemorrhage; GIB, gastrointestinal bleeding; MI, myocardial infarction; DOACs, direct oral anticoagulants; VKAs, vitamin-K antagonists; No. s, number of included studies.

The results of subgroup analyses in OSs should be interpreted cautiously as the included studies were limited in number for gender (1–2 studies), and some population (1–2 articles). For stroke/SE, compared with VKAs, DOACs showed a decreased risk in patients older than 80 years, and reduced risk in the USA and Taiwan population (Figure 4A and Supplementary Figures 18–22). For ICH, a similar result of reduced risk of DOACs vs. VKAs was found in all gender and age subgroups (Figure 4B and Supplementary Figures 23–25). For major bleeding, compared with VKAs, DOACs exhibited a reduced risk in populations based in Germany, Korea, and Taiwan (Figure 4C and Supplementary Figures 26–29). For GIB, in comparison to VKAs, DOACs increased in risk in women but not in men, meanwhile results indicated decreased risk in the Taiwanese population but not other populations (Figure 4D and Supplementary Figures 30–33). For all-cause mortality, DOACs demonstrated a similar risk compared to VKAs, except for populations in Korea and Taiwan, which showed a decreased risk (Figure 4E and Supplementary Figures 34–37). For MI, compared with VKAs, DOACs showed a significantly reduced risk in the Taiwan population (Figure 4F and Supplementary Figures 38–40). The comparability between primacy analysis and subgroup analysis did not find a significant difference (*P*_*interaction*_ > 0.05 for most of the subgroup analysis) (Supplementary Table 10).

### Sensitivity Analysis and Publication Bias

Sensitivity analyses involved sequentially excluding each study, confirming the robustness of primacy results (Supplementary Tables 11–16). Meta-regression analyses failed to detect potential effect modifiers to impact the outcomes (Supplementary Tables 17–22). No potential publication bias was observed by qualitative funnel plots as well as Begg's test and Egger's test (Supplementary Figures 41–46).

## Discussion

This study provides a comprehensive overview of the benefits and harms associated with DOACs in elderly AF patients. No significant difference in estimates for effectiveness and safety were found between OSs and RCTs. The pooled results from 27 OSs or 5 RCTs revealed that DOACs reduced the risk for stroke/SE, ICH, major bleeding, and MI, and had a similar risk for GIB and all-cause mortality when compared to VKAs. Notably, dabigatran increased risk for GIB by 48%.

Several previous meta-analyses have demonstrated the superior effectiveness and equivalent safety of DOACs vs. VKAs in elderly AF patients (28–31). However, the pooled results were mainly drawn from subgroup analysis of phase III clinical trials (RE-LY, ROCKET AF, ARISTOTLE, ENGAGE AF-TIMI 48, J-ROCKET AF) and failed to provide therapy choice on individual DOACs. RCTs and their meta-analyses represent the highest quality of evidence and are the basis for guidelines by healthcare organizations (32). However, RCTs are often conducted on specific populations or in specialized scenarios that differ from real clinical settings, yielding high internal validity (i.e., reliable relative treatment effect estimates) but low external validity (i.e., generalizability to real-world practice) (32). Real-world studies (RWSs), by integrating data from electronic health records (EHRs), claims databases, and disease registries have traditionally been considered methodologically weaker than RCTs (33). However, there is increased awareness that RWSs support and extend RCT findings to large patient populations in real-world clinical practice and, as such, are complementary to RCTs. Therefore, the evidence derived from RWSs and their meta-analyses may facilitate validation of conclusions drawn from RCTs and reassure decision-makers that findings can be extrapolated to real-world populations. In 2018, Bai et al. addressed this issue by merging OSs and found a reduced risk for major bleeding in AF patients aged ≥65 years (34). Nevertheless, the enrollment of patients aged 65–74 years may lead to underestimation of major bleeding risk when compared to those aged ≥75 years.

Recently, an updated meta-analysis of 20 OSs evaluated the benefits and harms of DOACs in AF patients aged ≥75 years (35). It should be noted that the results of effectiveness (composite outcomes of all strokes, or transient ischemic attack, or other thromboembolic events) and safety (composite outcomes of major bleeding, GIB, and ICH) were ambiguous, with positive results in fixed-effects models (HR: 0.95, 95%CI: 0.91–0.99 for effectiveness; HR: 0.97, 95%CI: 0.93–1.00 for safety) and negative results in random-effects models (HR: 0.93, 95%CI: 0.85–1.01 for effectiveness; HR: 0.95, 95%CI: 0.87–1.04 for safety). In addition, one study reported that the data of all-dose, reduced-dose, and standard-dose DOACs were repeatedly merged in this meta-analysis, which may lead to uncertain results (36). Considering the above limitations, the systematical reassessment of this topic is urgently required by a rigorous method.

In the present study, we used the random-effects model regardless of the presence of heterogeneity, performed the prior designed subgroup analyses, conducted sensitivity analyses, and included all available data from OSs and RCTs to comprehensively evaluate the benefits and harms of DOACs in the elderly. Our analysis demonstrated that the reduced risk for stroke/SE and ICH in OSs were in accordance with those in RCTs, thereby strengthening and replicating the conclusions. Although DOACs showed inconsistent results on major bleeding between OSs and RCTs, with reduced risk in OSs and similar risk in RCTs; this study suggests that DOACs might be safer than VKAs for the elderly. This finding may be attributed in part, to the prevalent use of reduced-dose DOACs in this fragile population, and the poor control of Time within Therapeutic Range (TTR) of VKAs in real clinical practice (37). Of note, dabigatran increased risk for GIB by 48% in elderly AF population, which may be partly explained by a decrease in the stomach function of the elderly and the direct anticoagulant effect on the gastric mucosa of dabigatran (38).

Moreover, this study has provided more detailed information than previous publications. For example, concerning gender, results indicated that the increased risk for GIB was limited to women, but not men. Women, especially those in the elderly population, have lean body weight and low creatinine clearance, thus leading to a higher serum level of DOACs, which makes them more prone to bleeding (39). In terms of age stratum, we found that there was no difference in the risk of stroke in patients aged >85 years. It is suggested that advanced age is the main risk factor for stroke and that impaired cognition limits adherence in the very elderly population (40), which may contribute to the reduced effectiveness of DOACs treatment. Despite this, DOACs may still be considered a more favorable choice when treating very elderly patients, due to the preferable trade-off between embolism and bleeding. Interestingly, DOACs showed a significantly decreased risk of stroke/SE and major bleeding in an Asian population, which could be partly attributable to the high prevalence of reduced-dose DOACs and the poor International Normalized Ratio (INR) stability among Asian people (41, 42). It is worth noting that a potential survival bias might be associated with all-cause mortality in different age stratum (aged >75, and >80). In response, we conducted meta-regression to explore the association between age and all cause mortality. The results failed to detect a potential effect of age on all-cause mortality, thus strengthening the robustness of results.

The main strength of this study was that it comprehensively assessed the benefits and harms of DOACs by comparing the results between high-quality OSs and RCTs. Several intrinsic limitations should be addressed in this study. Firstly, only two studies were specifically designed for the elderly and these had a small sample size, thus interpretation relied on subgroup data of included studies. Secondly, due to the lack of dosage information in most studies, the dosage subgroup was not performed in this study. In fact, because of the lean body weight and poor kidney function in the elderly, the reduced-dose DOACs were widely used in most populations, especially for Asian patients. Thirdly, due to the high risk of bias in OSs, we only included adjusted data to minimize confounding factors, but residual confounders may still exist in unmeasured variables. Fourthly, the number of studies for subgroup analysis was limited, inevitably leading to an insufficient estimation of sample size. Finally, the TTR of VKA users could not be obtained from included studies, and the relative superiority of DOACs might attribute to the poor control of VKAs.

In conclusion, compared with VKAs, DOACs were associated with reduced risk for stroke/SE, ICH, and major bleeding in the elderly with AF. The use of dabigatran had a 48% increase in risk for GIB. Considering the limitations of our analysis, further studies are needed to verify these findings.

## Data Availability Statement

The raw data supporting the conclusions of this article will be made available by the authors, without undue reservation.

## Author Contributions

Z-CG and JC were guarantors of the manuscript. Z-CG and N-NS contributed to the study conception and design, critical revision, and final approval of the published manuscript. YW, NW, L-CK, CZ, and J-LW contributed to data acquisition, analysis, and interpretation. All authors contributed to the article and approved the submitted version.

## Conflict of Interest

The authors declare that the research was conducted in the absence of any commercial or financial relationships that could be construed as a potential conflict of interest.
