# Supplementary material for: Direct Oral Anticoagulants vs. Vitamin-K Antagonists in the Elderly With Atrial Fibrillation: A Systematic Review Comparing Benefits and Harms Between Observational Studies and Randomized Controlled Trials
Source: Front Cardiovasc Med. 2020 Sep 10;7:132. doi: 10.3389/fcvm.2020.00132 (PMC7511536; doi:10.3389/fcvm.2020.00132)
Supplement: Supplementary file 1 [file Data_Sheet_1.PDF]

SUPPLEMENTAL FILE

Title: Direct oral anticoagulants versus vitamin-K antagonists in the elderly with atrial fibrillation: a systematic review comparing benefits and harms between observational studies and randomized controlled trials.

Contents

Tables

Supplementary Table 1. Search strategy used in July 1, 2019 6

Supplementary Table 2. Excluded studies with reasons 8

Supplementary Table 3. Characteristics of OSs 12

Supplementary Table 4. Patient demographics and clinical characteristics of OSs 16

Supplementary Table 5. Patient bleeding history and concomitant drugs of OSs 18

Supplementary Table 6. Characteristics of RCTs 20

Supplementary Table 7. Patient demographics and clinical characteristics of RCTs 21

Supplementary Table 8. Quality assessment of OSs 22

Supplementary Table 9. Quality assessment of RCTs 24

Supplementary Table 10. The comparability between primacy analysis and subgroup analysis in OSs........ 25

Supplementary Table 11. Sensitivity analysis of Stroke/SE in OSs 26

Supplementary Table 12. Sensitivity analysis of ICH in OSs 27

Supplementary Table 13. Sensitivity analysis of major bleeding in OSs 28

Supplementary Table 14. Sensitivity analysis of GIB in OSs 29
Supplementary Table 15. Sensitivity analysis of all-cause mortality in OSs 30
Supplementary Table 16. Sensitivity analysis of MI in OSs 31
Supplementary Table 17. Meta-regression analysis of Stroke/SE in OSs 32
Supplementary Table 18. Meta-regression analysis of ICH in OSs 34
Supplementary Table 19. Meta-regression analysis of major bleeding in OSs 36
Supplementary Table 20. Meta-regression analysis of GIB in OSs 38
Supplementary Table 21. Meta-regression analysis of all cause mortality in OSs 40
Supplementary Table 22. Meta-regression analysis of MI in OSs 42

Figures

Supplementary Figure 1. Stroke/SE of RCTs 44
Supplementary Figure 2. ICH of RCTs 45
Supplementary Figure 3. Major bleeding of RCTs 46
Supplementary Figure 4. GI bleeding of RCTs 47
Supplementary Figure 5. All-cause mortality of RCTs 48
Supplementary Figure 6. Stroke/SE of OSs 49
Supplementary Figure 7. ICH of OSs 50
Supplementary Figure 8. Major bleeding of OSs 51
Supplementary Figure 9. GI bleeding of OSs 52
Supplementary Figure 10. All-cause mortality of OSs 53

Supplementary Figure 11. MI of OSs ... 54
Supplementary Figure 12. Stroke/SE in rivaroxaban/dabigatran/apixaban/edoxaban (OSs) ... 55
Supplementary Figure 13. ICH in rivaroxaban/dabigatran/apixaban/edoxaban (OSs) ... 56
Supplementary Figure 14. Major bleeding in rivaroxaban/dabigatran/apixaban/edoxaban (OSs) ... 57
Supplementary Figure 15. GI bleeding in rivaroxaban/dabigatran/apixaban/edoxaban (OSs) ... 58
Supplementary Figure 16. All-cause mortality in rivaroxaban/dabigatran/apixaban/edoxaban (OSs) ... 59
Supplementary Figure 17. MI in rivaroxaban/dabigatran/apixaban/edoxaban (OSs) ... 60
Supplementary Figure 18. Stroke/SE by gender of OSs ... 61
Supplementary Figure 19. Stroke/SE by country or region of OSs ... 62
Supplementary Figure 20. Stroke/SE of age>80 (OSs) ... 63
Supplementary Figure 21. Stroke/SE of age>85 (OSs) ... 64
Supplementary Figure 22. Stroke/SE of age>90 (OSs) ... 65
Supplementary Figure 23. ICH by gender of OSs ... 66
Supplementary Figure 24. ICH by country or region of OSs ... 67
Supplementary Figure 25. ICH of age>80 (OSs) ... 68
Supplementary Figure 26. Major bleeding by country or region of OSs ... 69
Supplementary Figure 27. Major bleeding of age>80 (OSs) ... 70
Supplementary Figure 28. Major bleeding of age>85 (OSs) ... 71
Supplementary Figure 29. Major bleeding of age>90 (OSs) ... 72
Supplementary Figure 30. GI bleeding by gender of OSs ... 73

Supplementary Figure 31. GI bleeding by country or region of OSs 74
Supplementary Figure 32. GI bleeding of age>80 (OSs) 75
Supplementary Figure 33. GI bleeding of age>85 (OSs) 76
Supplementary Figure 34. All-cause mortality by gender of OSs 77
Supplementary Figure 35. All-cause mortality by country or region of OSs 78
Supplementary Figure 36. All-cause mortality of age>80 (OSs) 79
Supplementary Figure 37. All-cause mortality of age>85 (OSs) 80
Supplementary Figure 38. MI by country or region of OSs 81
Supplementary Figure 39. MI of age>80 (OSs) 82
Supplementary Figure 40. MI of age>85 (OSs) 83
Supplementary Figure 41. Funnel Plot of OSs (Stroke/SE) 84
Supplementary Figure 42. Funnel Plot of OSs (ICH) 85
Supplementary Figure 43. Funnel Plot of OSs (major bleeding) 86
Supplementary Figure 44. Funnel Plot of OSs (GI bleeding) 87
Supplementary Figure 45. Funnel Plot of OSs (all-cause mortality) 88
Supplementary Figure 46. Funnel Plot of OSs (MI) 89
References 90

Abbreviations in Supplementary File

RCT: Randomized controlled trial; OSs: observational studies; AF: atrial fibrillation; VTE: venous thromboembolism; Dabi: dabigatran; Riv: rivaroxaban; Api: apixaban; Edo: edoxaban; RR: relative risk; HR: hazard ratio; CI: confidence interval; SE: Systemic embolism; GIB: gastrointestinal bleeding; ICH: intracranial hemorrhage; MI: myocardial infarction; NCT: national clinical trial; BMI: Body Mass Index; Ccr: creatinine clearance rate; CKD: chronic kidney disease; DM: Diabetes; HF: heart failure; HBP: hypertension; NR: not reported; INR: international normalized ratio; TIA: transient ischemic attack; CA: covariate adjustment; IPTW: inverse probability of treatment weighting; DOACs: direct Oral Anticoagulants; PSA: propensity score adjustment; PSM: propensity score matching; VKA: vitamin k antagonist; ACEI: Angiotensin-converting enzyme inhibitor; ARB: Angiotensin receptor inhibitor; Antip-drugs: Antiplatelet agents; Antia-drugs: Antiarrhythmic drugs; Asp: Aspirin; Amio: Amiodarone; CCB: calcium channel blocker; Clo: Clopidogrel; Dil: Diltiazem; Dron: Dronedarone; Estr: Estrogen; Glu: Glucocorticoids; H2: H2-receptor antagonist; NSAIDS: Non-steroidal anti-inflammatory drugs; PPI: Proton pump inhibitor; SSRI: Serotonin receptor antagonist; Vera: Verapamil; Low: low risk; Moderate: moderate risk: unclear risk; High: high risk.

Supplementary Table 1. Search strategy used in July 1, 2019

Literature databases	Search items	Items found
MEDLINE	“dabigatran”[MeSH Terms] OR “dabigatran”[Title/Abstract] OR “Pradaxa”[Title/Abstract] OR “rivaroxaban”[MeSH Terms] OR “rivaroxaban”[Title/Abstract] OR “Xarelto”[Title/Abstract] OR “apixaban” [MeSH Terms] OR “apixaban”[Title/Abstract] OR “Eliquis”[Title/Abstract] OR “edoxaban”[MeSH Terms] OR “edoxaban”[Title/Abstract] OR “Savaysa”[Title/Abstract]) OR “betrixaban”[MeSH Terms] OR “betrixaban”[Title/Abstract] OR “Bevyxxa”[Title/Abstract]) OR “Non-vitamin K antagonist oral anticoagulants”[Title/Abstract] OR “NOACs”[Title/Abstract]) OR “direct oral anticoagulants”[Title/Abstract]) OR “DOACs”[Title/Abstract]) OR “novel oral anticoagulants”[Title/Abstract]) OR “new oral anticoagulants”[Title/Abstract]) OR “factor Xa inhibitors”[Title/Abstract]) OR “factor IIa inhibitors”[Title/Abstract]	11002
EMBASE	‘dabigatran’/exp OR ‘dabigatran’:ti,ab,kw OR ‘Pradaxa’:ti,ab,kw OR ‘rivaroxaban’/exp OR ‘rivaroxaban’: ti,ab,kw OR ‘Xarelto’: ti,ab,kw OR ‘apixaban’/exp OR ‘apixaban’: ti,ab,kw OR ‘Eliquis’: ti,ab,kw OR edoxaban’/exp OR ‘edoxaban’: ti,ab,kw OR ‘Savaysa’: ti,ab,kw OR ‘betrixaban’/exp OR ‘betrixaban’: ti,ab,kw OR ‘Bevyxxa’: ti,ab,kw OR ‘Non-vitamin K antagonist oral anticoagulants’: ti,ab,kw OR ‘NOACs’: ti,ab,kw OR ‘direct oral anticoagulants’: ti,ab,kw OR ‘DOACs’: ti,ab,kw OR ‘novel oral anticoagulants’: ti,ab,kw OR ‘new oral anticoagulants’: ti,ab,kw OR	11718

	‘factor Xainhibitors’: ti,ab,kw OR ‘factor IIa inhibitors’: ti,ab,kw	
COCHRANE	MeSH descriptor: [dabigatran] OR dabigatran: ti,ab,kw OR Pradaxa:ti,ab,kw OR MeSH descriptor: [rivaroxaban] OR rivaroxaban: ti,ab,kw OR Xarelto: ti,ab,kw OR MeSH descriptor: [apixaban] OR apixaban: ti,ab,kw OR Eliquis: ti,ab,kw OR MeSH descriptor: [edoxaban] OR edoxaban: ti,ab,kw OR Savaysa: ti,ab,kw OR MeSH descriptor: [betrixaban] OR betrixaban: ti,ab,kw OR Bevyxxa: ti,ab,kw OR Non-vitamin K antagonist oral anticoagulants: ti,ab,kw OR NOACs: ti,ab,kw OR direct oral anticoagulants: ti,ab,kw OR DOACs: ti,ab,kw OR novel oral anticoagulants: ti,ab,kw OR new oral anticoagulants: ti,ab,kw OR factor Xa inhibitors: ti,ab,kw OR factor IIa inhibitors: ti,ab,kw	3089
Overall		25809
Duplication		5430

Supplementary Table 2. Excluded studies with reasons

**Study**	**Drugs**	**Reason for exclusion**
Yoshimura 2018 (Yoshimura et al., 2018)	DOACs	Not reported adjusted data
Yavuz 2016 (Yavuz et al., 2016)	Dabigatran	Not reported elderly data
Yamashita 2012 (Yamashita et al., 2012)	Edoxaban	Not reported elderly data
Weitz 2010 (Weitz et al., 2010)	Edoxaban	Not reported elderly data
Weir 2017 (Weir et al., 2017)	Rivaroxaban	Not reported elderly data
van Rein 2019 (van Rein et al., 2019)	DOACs	No reported elderly data
Vinogradova 2018 (Vinogradova et al., 2018)	DOACs	Not reported elderly data
Villines 2019 (Villines et al., 2019)	DOACs	Not reported elderly data
Staerk 2018 (Staerk et al., 2018)	DOACs	Not reported elderly data
Stolk 2017 (Stolk et al., 2017)	DOACs	Not reported elderly data
Steinberg 2018 (Steinberg et al., 2018)	DOACs	Not reported elderly data
Staerk 2015 (Staerk et al., 2015)	Antithrombotic agents	Not DOACs study
Sorensen 2013 (Sorensen et al., 2013)	Dabigatran	Not reported elderly data
Song 2017 (Song et al., 2017)	Dabigatran	Not reported elderly data
Siontis 2018 (Siontis et al., 2018)	Rivaroxaban	Patients concomitant with dialysis

Shah 2018 (Shah et al., 2018)	DOACs	Patients concomitant with cancer
Schafer 2018 (Schafer et al., 2018)	Apixaban	Single center study
Palamaner 2017 (Palamaner Subash Shantha et al., 2017)	DOACs	Not reported elderly data
Okumura 2016 (Okumura et al., 2016)	Rivaroxaban	Not reported elderly data
Ogawa 2011 (Ogawa et al., 2011)	Apixaban	Only reported adjusted data
Nielsen 2017 (Nielsen et al., 2017)	DOACs	Not reported adjusted data
Moustafa 2018 (Moustafa et al., 2018)	DOACs	Not reported elderly data
Meng 2019 (Meng et al., 2019)	Dabigatran/ Rivaroxaban	Not reported elderly data
Martinez 2018 (Martinez et al., 2018)	DOACs	Not reported elderly data
Lip 2016 (Lip et al., 2016)	DOACs	Overlapping period with Lip 2018(Lip et al., 2018)
Li 2017(Li et al., 2017)	Dabigatran and rivaroxaban	Single center study
Leschke 2017 (Leschke et al., 2017)	Rivaroxaban	One arm study
Larsen 2016 (Larsen et al., 2016)	Apixaban	Not reported elderly data
Lamsam 2018 (Lamsam et al., 2018)	DOACs	Not reported elderly data
Lamberts 2017 (Lamberts et al., 2017)	DOACs	Not reported elderly data
Lailiberte 2014 (Laliberte et al., 2014)	DOACs	Not reported adjusteddata

Lai 2017 (Lai et al., 2017)	Dabigatran and rivaroxaban	DOACs as control
Kwon 2016 (Kwong et al., 2017)	Rivaroxaban	One reported adjusted data
Korenstra 2016 (Korenstra et al., 2016)	Dabigatran	Single center study
Kohsaka 2018 (Kohsaka et al., 2018)	Apixaban	Not reported elderly data
Korestune 2019 (Koretsune et al., 2019)	DOACs	Not reported elderly data
Jung 2019 (Jung et al., 2019)	DOACs	Patients concomitant with hypertrophic cardiomyopathy
Hsu 2018 (Hsu et al., 2018)	Dabigatran and rivaroxaban	Patients concomitant with diabetes
Ho 2015 (Ho et al., 2015)	Dabigatran	Not reported adjusted data
Harel 2016 (Harel et al., 2016)	DOACs	Patients concomitant with CKD
Gieling 2017 (Gieling et al., 2017)	DOACs	Not reported elderly data
Ellis2016 (Ellis et al., 2016)	Dabigatran and rivaroxaban	Not reported elderly data
Deitelzweig 2017 (Deitelzweig et al., 2017)	Apixaban	Not reported elderly data
Connolly 2013 (Connolly et al., 2013)	Betrixaban	Not reported elderly data
Coleman 2018 (Coleman et al., 2018)	Apixaban	Not reported adjusted data
Coleman 2016 (Coleman et al., 2016)	Rivaroxaban and apixaban	Not reported elderly data
Chan 2016 (Chan et al., 2016)	Dabigatran	Overlapping period with Chan 2019(Chan et al., 2019)
Chan 2015 (Chan et al., 2015)	Dabigatran and rivaroxaban	Overlapping period with Chan 2019(Chan et al., 2019)

Cappato 2015 (Cappato et al., 2015)	Rivaroxaban	Not reported elderly data
Becattini 2017 (Becattini et al., 2017)	DOACs	Not reported elderly data
Arihiro 2016 (Arihiro et al., 2016)	DOACs	Not reported adjusted data
Andersson 2018 (Andersson et al., 2018)	DOACs	DOACs as control and not reported elderly data
Amin 2018 (Amin et al., 2018)	DOACs	Not reported elderly data
Abraham 2013 (Abraham et al., 2013)	Antithrombotic treatment	Not DOACsstudy
Abe 2015 (Abe et al., 2015)	Dabigatran	Not reported adjusted GIB data
Yamashita 2017 (Yamashita et al., 2017)	DOACs	Not reported elderly data

GIB: gastrointestinal bleeding; DOACs: direct oral anticoagulants; CKD: chronic kidney disease.

Supplementary Table 3. Characteristics of OSs

Study	Country or region/data source/inclusion period	Interventions/ Numbers	Controls/ Numbers	Adjusted method	Follow-up	Age stratification
Lindsay 2014 (Lindsay et al., 2014)	USA/Truven Health Marketscan Research Databases/2009.1.1-2012.12.31	Dabigatran/287	Warfarin/1219	PSA	At least 6months	> 77 y
Abraham 2015 (Abraham et al., 2015)	USA/Optum Labs Data Warehouse/2010.11.1-2013.9.30	Dabigatran; Rivaroxaban/3645	Warfarin/3677	PSM	NR	≥ 76y
Graham 2014 (Graham et al., 2014)	USA/Beneficiary Base and Chronic Conditions segments/2010.10.19-2012.12.31	Dabigatran/39208	Warfarin/39445	PSM	At least 6 months	≥ 75y
Hernandez 2015 (Hernandez et al., 2015)	USA/Centers for Medicare and Medicaid Services (CMS)/ 2010. 10. 1-2011.10.31	Dabigatran/1302	Warfarin/8102	IPTW	More than 60 days	≥ 75y
Lauffenburger 2015 (Lauffenburger et al., 2015)	USA/ Truven Health MarketScan Commercial Claims and Encounters and Medicare supplement database/2009-2012	Dabigatran/2853	Warfarin/8297	IPTW	358days	≥ 75y
Seeger 2015 (Seeger et al., 2015)	USA/MarketScan, Truven and Clinformatics, Optum/2010.10-2012.12	Dabigatran/5307	Warfarin/4991	PSM	2 years	≥ 75y
Maura 2015 (Maura et al., 2015)	French medico-administrative databases (SNIIRAM-PMSI)/2012.7.20-2012.11.30	Dabigatran/4573	Warfarin/8685	PSM	3 months	≥ 75y
Avgil-Tsadok 2016 (Avgil-Tsadok et al., 2016)	Canada/Provincial hospital discharge database/1999.1.1-2013.3.31	Dabigatran/9548	Warfarin/32930	PSA	NR	≥ 75y

Adeboyeje 2017 (Adeboyeje et al., 2017)	USA/HealthCore Integrated Research Environment/2009.11.1-2016.1.31	Dabigatran; Rivaroxaban; Apixaban /5498	Warfarin/9278	IPTW	NR	≥ 75y
Lau 2017 (Lau et al., 2017)	Hong Kong hospital /2010-2014	Dabigatran/2580	Warfarin/2580	PSM	425days	≥ 75y
Bengtson 2017 (Bengtson et al., 2017)	USA/MarketScan/2009.1.1-2012.12.31	Dabigatran/32918	Warfarin/10116 7	PSA	15months	≥ 75y
Cha 2017 (Cha et al., 2017)	Korea/National Health Insurance Service database/2014.1-2015.12	Dabigatran; Rivaroxaban; Apixaban /4045	Warfarin/9828	PSM	1.51 year	≥ 75y
Friberg 2017 (Friberg and Oldgren, 2017)	Sweden/National Swedish Patient Register, Dispensed Drug Register, Cause of Death Register and the socioeconomic longitudinal integration database /2011.12.1-2014.12.31	Dabigatran; Rivaroxaban; Apixaban/18638	Warfarin/49418	PSA	NR	≥ 75y
Go 2017 (Go et al., 2017)	USA/National U.S. Food and Drug Administration Sentinel network/2010.11.1-2014.5.31	Dabigatran/25289	Warfarin/25289	PSM	123days	≥ 75y
Nielsen 2017 (Nielsen et al., 2017)	Denmark/the Danish national prescription registry, the Danish civil registration system, the Danish national patient register	Dabigatran; Rivaroxaban; Apixaban /29323	Warfarin/29912	IPTW	1 year	≥ 75y
Norby 2017 (Norby et al., 2017)	USA/MarketScan/2010.1.1-2014.12.31	Rivaroxaban/22373	Warfarin/35760	PSA	1 year	≥ 75y
Chao 2018 (Chao et al., 2018)	Taiwan/National Health Insurance Research Database/2012-2015	DOACs/978	Warfarin/768	PSM	2.06±2.15 years	≥ 90y
Forslund 2018 (Forslund et al., 2018)	Sweden/administrative health data register of the Stockholm Region/2012.1-2015.12	Dabigatran; Rivaroxaban; Apixaban /2207	Warfarin/4445	IPTW	365 days	≥80y

Lai 2018 (Lai et al., 2018)	Taiwan/Taiwan National Health Insurance Database/2012.6.1-2015.5.31	Dabigatran; Rivaroxaban/2387	Warfarin/2387	PSM	6.6months	≥85y
Lee 2018 (Lee et al., 2018)	Korea/National Health Insurance Service database/2014.01-2016.12	Edoxaban/1467	Warfarin/4641	PSM	1 year	≥ 75y
Lip 2018 (Lip et al., 2018)	USA/Centers for Medicare and Medicaid Services Medicare data and MarketScan, PharMetrics, Optum, Humana/2013.1.1-2015.9.30	Dabigatran; Rivaroxaban; Apixaban /83291	Warfarin/33906	PSM	1 year	≥ 75y
Ujeyl 2018 (Ujeyl et al., 2018)	Germany/health insurance fund AOK/2012.1.1-2013.12.31	Dabigatran; Rivaroxaban; Apixaban /52570	Phenprocoumon/ 52570	PSM	249-305days	≥ 75y
Zoppellaro 2018 (Zoppellaro et al., 2018)	Italy/linked claims data in the Veneto Region using the drug prescriptions archive, the regional inpatients register, the database of residents registered in the regional health system and the archive of co-payment exemptions/2013.7-2015.12	Dabigatran; Rivaroxaban; Apixaban/2882	VKA/12254	PSA	At least 3 months	≥ 75y
Giustozzi 2019 (Giustozzi et al., 2019)	Italian multiple registry of AF center/2013.8-	DOAC/245	VKA/301	PSM	404	≥ 90y
Hohmann 2019 (Hohmann et al., 2019)	Germany/the Institute for Applied Health Research (InGef) research database/2013.1.1-2016.6.30	Apixaban; Dabigatran; Rivaroxaban/42562	Phenprocoumon/ 27939	PSA	856 days	≥ 75y
Patti 2019 (Patti et al., 2019)	multiple countries/PREFER in AF and PREFER in AF PROLONGATION/2014.6-2016.6	DOAC/1556	VKA/2269	CA	12 months	≥ 75y
Chan 2019 (Chan et al., 2019)	Taiwan National Health Insurance Research Database (NHIRD)/2012.6.01-2017.12.31	DOAC/34961	VKA/980	PSM	16 months	≥ 75y

OSs: observational studies; AF: atrial fibrillation; CA: covariate adjustment; IPTW: inverse probability of treatment weighting; DOACs: direct oral anticoagulants; PSA: propensity score adjustment; PSM: propensity score matching; VKA: vitamin k antagonist.

Supplementary Table 4. Patient demographics and clinical characteristics of OSs

Study	Total number	Mean age (y)	Female (%)	HF (%)	HBP (%)	DM	Stroke/ TIA (%)	MI (%)	Renal disease (%)	Liver disease (%)	Anemia (%)	Cancer (%)	CHADS$_2$ (mean)	CHADS2-VASc (mean)	HAS-BLED (mean)	HAS-BLED >3 (%)
Lindsay 2014	1260	75.6	45.5	91	NR	34.7	39.4	11.5	20	8	NR	NR	3.1	4.55	3	NR
Abraham 2015 (Dabi)	4131	64.6	37.2	19.3	NR	28.6	NR	9.4	6.7	NR	NR	NR	NR	NR	NR	22.9
Abraham 2015 (Riva)	3191	60.4	56.1	4.1	NR	19.7	NR	2.1	4.3	NR	NR	NR	NR	NR	NR	20.3
Graham 2014	78653	NR	51	18	87	33	10	2	18	NR	NR	NR	NR	NR	NR	41
Hernandez 2015	9404	75.1	57.9	51.1	87.6	43.9	22.5	8.6	32.9	NR	NR	NR	NR	NR	NR	NR
Lauffenburger 2015	11150	NR	40.1	25.5	72	30.5	NR	3.9	10.8	NR	17.3	NR	NR	NR	NR	NR
Seeger 2015	10298	68	37.8	17.8	96.3	20.6	NR	4.1	10	2.8	NR	10.2	1.1	1.5	1	NR
Maura 2015	13258	74	46	23	NR	19	9	NR	21	NR	NR	NR	NR	3.2	2.3	NR
Avgil-Tsadok 2016 (Dabi)	42478	NR	54.9	29.7	77.5	24.7	11.6	17.7	23.1	4.5	NR	11	NR	3.6	2.5	46
Lau 2017	5160	73.8	42.3	20.4	52.9	24.3	32.1	3.9	5.3	NR	NR	NR	2	2.2	NR	NR
Adeboyeje2017 (Dabi)	3433	70	40.9	27.8	59.8	28.4	NR	NR	10.1	4.7	NR	NR	NR	NR	2.1	NR
Adeboyeje2017 (Riva)	3326	70	40.9	27.8	59.8	28.4	NR	NR	10.1	4.7	NR	NR	NR	NR	2.1	NR
Adeboyeje2017 (Api)	1483	70	40.9	27.8	59.8	28.4	NR	NR	10.1	4.7	NR	NR	NR	NR	2.1	NR
Bengtson2017	56688	68.5	36.2	24.3	75.2	28.6	20.6	7.6	7.6	4.8	NR	1.6	2	NR	NR	NR
Cha 2017 (Riva)	2029	70.5	47.3	44.3	75.7	23.8	NR	4.3	NR	NR	NR	NR	NR	3.6	NR	NR
Cha 2017 (Dabi)	1215	69.3	42	45	76.8	26.5	NR	4.3	NR	NR	NR	NR	NR	3.51	NR	NR
Cha 2017 (Api)	801	70.3	45.6	43	76.9	23.6	NR	5.3	NR	NR	NR	NR	NR	3.57	NR	NR
Friberg2017	44597	73.4	45.6	19.5	61.1	16.1	19.5	10.8	2.5	1.4	8.8	8.6	NR	3.2	NR	NR
Go 2017	3296	68.5	36.1	38.6	81.6	30.1	8.1	4.9	11.6	0.3	5.9	1.2	NR	NR	NR	NR
Hernandez2017 (Dabi)	4921	NR	41	60	94.9	47	NR	8.8	NR	NR	NR	NR	NR	NR	NR	NR
Hernandez2017 (Riva)	4810	NR	42	60	94.9	47	NR	8.8	NR	NR	NR	NR	NR	NR	NR	NR
Nielsen 2017(Api)	9315	83.9	60.6	20.3	63.5	17.3	NR	NR	9.5	NR	NR	22.2	NR	4.3	2.8	NR
Nielsen 2017(Dabi)	14607	79.9	60.6	15.5	64	14.9	NR	NR	3.9	NR	NR	18.3	NR	3.8	2.7	NR

Nielsen 2017(Riva)	5401	77.9	60.6	18.9	58.1	16.5	NR	NR	9.1	NR	NR	20	NR	3.6	2.5	NR
Norby2017	58133	69.3	38.7	23.1	66	25.7	15.5	7.1	7.6	3.6	NR	1.6	NR	3	NR	NR
Chao 2018	1746	92.3	55.4	48.8	42.6	34.6	40.4	NR	14.6	1.8	NR	17.8	NR	5.2	NR	NR
Forslund 2018	6652	73.5	44	24.6	68.9	18.6	20.8	NR	6.5	1.7	14.7	22.1	NR	3.55	NR	NR
Lai 2018	4774	88.6	52	27.8	51.3	15.9	14.5	1.4	NR	NR	1.1	NR	2.2	3.8	NR	NR
Lee 2018	6108	70.5	44.5	23.8	68.4	20.3	NR	2.2	NR	NR	NR	NR	1.67	3.23	NR	NR
Lip 2018 (Api)	29891	74.3	45.9	28.5	NR	34.8	12.1	NR	23	NR	NR	NR	NR	3.7	3	61.4
Lip 2018 (Dabi)	10735	71.9	41.2	24.5	NR	35	10.1	NR	16.2	NR	NR	NR	NR	3.4	2.7	52.3
Lip 2018 (Riva)	42665	74.4	44.9	27.8	NR	35.8	11.7	NR	20.4	NR	NR	NR	NR	3.7	2.9	60.3
Ujeyl2018 (Dabi)	27438	75.5	54	30.2	82.3	37.3	15	4	17.2	0.3	7.6	14.8	NR	NR	NR	5
Ujeyl2018 (Riva)	71338	75.5	54	30.2	82.3	37.3	15	4	17.2	0.3	7.6	14.8	NR	NR	NR	5
Ujeyl2018 (Api)	6364	75.5	54	30.2	82.3	37.3	15	4	17.2	0.3	7.6	14.8	NR	NR	NR	5
Zoppellaro2018	15136	NR	37.4	15	81	17.2	26.7	2.7	4	1.1	NR	9.8	NR	4.3	2.7	NR
Giustozzi 2019	546	92	63	36	85	17	28	NR	7	4	NR	NR	2.86	4.93	2.59	9.8
Hohmann 2019	37816	81.5	54.3	45.4	76.6	35.4	17.8	NR	NR	NR	NR	23.3	2.8	4.7	3	NR
Patti 2019	3825	60.4	48	31	81		19.5	NR	23	1.3	NR	NR	NR	4.4	2.32	NR

OSs: observational studies; AF: atrial fibrillation; DOACs: direct oral anticoagulants; VKA: vitamin k antagonist; Dabi: dabigatran; Riv: rivaroxaban; Api: apixaban; Edo: edoxaban;BMI: Body Mass Index; Ccr: creatinine clearance rate; CKD: chronic kidney disease; DM: Diabetes; HF: heart failure; HBP: hypertension; NR: not reported; TIA: transient ischemic attack; NR: not reported.

Supplementary Table 5. Patient bleeding history and concomitant drugs of OSs

Study	Prior bleeding	Prior GI bleeding	ACEI /ARB	Beta-bl ocker	Dil	Vera	CCB	Am io	Dr on	Dig oxin	Antia- drugs	Stat in	Antip- drugs	Asp	Clo	NSA IDS	PPI	H2	SSRI	Glu	Estr
Lindsay 2014	NR	12.6	32	79	62.8	NR	45.8	NR	NR	31	NR	NR	14.5	NR	NR	NR	NR	NR	NR	NR	NR
Abraham 2015 (Dabi)	NR	0.8	0.4	NR	NR	NR	NR	NR	NR	NR	NR	NR	NR	17.5	NR	NR	22.1	22.5	NR	16.1	17.8
Abraham 2015 (Riva)	NR	0.8	0.5	NR	NR	NR	NR	NR	NR	NR	NR	NR	NR	4.4	NR	NR	50	22.2	NR	22.5	23.6
Graham 2014	4	NR	NR	59	70		2	42	10	5	17	25	57	17	NR	NR	15	26	5	13	NR
Hernandez 2015	9.5	NR	NR	NR	NR	NR	NR	NR	NR	NR	NR	NR	NR	8	NR	NR	8.6	NR	NR	NR	NR
Lauffenburger 2015	12.6	NR	57.2	67.5	NR	NR	42	NR	NR	16.5	NR	54.5	13.2	NR	NR	NR	NR	NR	NR	NR	NR
Seeger 2015	NR	2.1	58	73	NR	NR	42	NR	NR	NR	NR	51.8	13.1	NR	13.1	21.1	22.8	22.4	NR	NR	NR
Maura 2015	3	NR	NR	NR	NR	NR	NR	NR	NR	NR	NR	NR	53	NR	NR	19	NR	NR	NR	NR	NR
Avgil-Tsadok 2016 (Dabi)	9.3	NR	NR	19.3	40.2	12.1	1.5	NR	7	NR	16.9	2.1	21.2	19.3	17.1	2.2	0.5	NR	NR	NR	NR
Lau 2017	NR	NR	45.9	61.4	NR	NR	NR	10.5	0.8	NR	NR	50.5	NR	70.2	6.4	5.2	35	58	2.3	NR	NR
Adeboyeje2017 (Dabi)	14.9	NR	NR	NR	NR	NR	NR	17.5	NR	NR	NR	NR	NR	48.8	46.5	9.5	24.6	NR	NR	NR	NR
Adeboyeje2017 (Riva)	14.9	NR	NR	NR	NR	NR	NR	18.9	NR	NR	NR	NR	NR	56.5	53.1	9.7	28.7	NR	NR	NR	NR
Adeboyeje2017 (Api)	14.9	NR	NR	NR	NR	NR	NR	18.1	NR	NR	NR	NR	NR	53.7	50.8	9.9	23	NR	NR	NR	NR
Bengtson2017	11.2	NR	7.6	59.5	71.1	NR	NR	41.7	NR	NR	14.9	29.4	54.3	16.1	2.1	14	NR	NR	NR	NR	NR
Cha 2017 (Riva)	NR	NR	NR	NR	NR	NR	NR	NR	NR	NR	NR	NR	NR	NR	NR	NR	NR	NR	NR	NR	NR
Cha 2017 (Dabi)	NR	NR	NR	NR	NR	NR	NR	NR	NR	NR	NR	NR	NR	NR	NR	NR	NR	NR	NR	NR	NR
Cha 2017 (Api)	NR	NR	NR	NR	NR	NR	NR	NR	NR	NR	NR	NR	NR	NR	NR	NR	NR	NR	NR	NR	NR
Friberg 2017	10.2	3.4	3.4	NR	NR	NR	NR	NR	NR	NR	NR	NR	NR	NR	NR	NR	NR	NR	NR	NR	NR
Go 2017	1	NR	1.1	60.1	71.5	NR	NR	40.9	NR	NR	NR	34.5	53.2	13.2	0.9	NR	21.1	25.2	NR	NR	NR
Hernandez2017 (Dabi)	12.7	NR	NR	NR	NR	NR	NR	NR	NR	NR	NR	NR	7.1	10.7	NR	12.5	11.8	NR	NR	NR	NR
Hernandez2017 (Riva)	12.7	NR	NR	NR	NR	NR	NR	NR	NR	NR	NR	NR	7.1	12.5	NR	12.5	12.7	NR	NR	NR	NR
Nielsen 2017 (Api)	17.3	NR	NR	60	NR	3	33.8	4.3	NR	NR	NR	NR	NR	48.2	NR	NR	NR	NR	NR	NR	NR
Nielsen 2017 (Dabi)	14.3	NR	NR	62.1	NR	5.1	35.6	3.5	NR	NR	NR	NR	NR	50.3	NR	NR	NR	NR	NR	NR	NR
Nielsen 2017 (Riva)	15	NR	NR	50.5	NR	2.7	30.5	3.4	NR	NR	NR	NR	NR	44.4	NR	NR	NR	NR	NR	NR	NR
Norby2017	6.8	4.4	4.4	50.2	63.9	NR	NR	36	NR	NR	11.4	20.2	46.6	11.3	1.8	9.5	NR	NR	NR	NR	NR
Chao 2018	8.6	NR	NR	NR	NR	NR	NR	NR	NR	NR	NR	NR	NR	NR	NR	NR	NR	NR	NR	NR	NR

Forslund 2018	7.6	NR	NR	NR	NR	NR	NR	NR	NR	NR	NR	NR	4.83	NR	5.5	NR	NR	NR	NR	NR	NR
Lai 2018	NR	NR	NR	59.5	41.7	21.3	2.7	39.7	15.7	2.2	20.3	NR	17	54.9	44.7	10.2	52.9	12	32	NR	NR
Lee 2018	NR	NR	NR	NR	NR	NR	NR	NR	NR	NR	NR	NR	NR	NR	NR	NR	NR	NR	NR	NR	NR
Lip 2018 (Api)	NR	NR	NR	NR	NR	NR	NR	NR	NR	NR	NR	NR	NR	NR	NR	NR	NR	NR	NR	NR	NR
Lip 2018 (Dabi)	NR	NR	NR	NR	NR	NR	NR	NR	NR	NR	NR	NR	NR	NR	NR	NR	NR	NR	NR	NR	NR
Lip 2018 (Riva)	NR	NR	NR	NR	NR	NR	NR	NR	NR	NR	NR	NR	NR	NR	NR	NR	NR	NR	NR	NR	NR
Ujeyl2018 (Dabi)	8.3	NR	NR	NR	NR	NR	NR	NR	NR	NR	NR	NR	NR	16	NR	NR	17.2	33.6	1.2	5.3	NR
Ujeyl2018 (Riva)	8.3	NR	NR	NR	NR	NR	NR	NR	NR	NR	NR	NR	NR	16	NR	NR	17.2	33.6	1.2	5.3	NR
Ujeyl2018 (Api)	8.3	NR	NR	NR	NR	NR	NR	NR	NR	NR	NR	NR	NR	16	NR	NR	17.2	33.6	1.2	5.3	NR
Zoppellaro2018	4.2	NR	NR	NR	NR	NR	NR	NR	NR	NR	NR	NR	NR	49.9	NR	NR	17.5	NR	NR	NR	NR
Giustozzi 2019	18.3	NR	NR	NR	NR	NR	NR	NR	NR	NR	NR	NR	NR	50.9	NR	NR	18.5	NR	NR	NR	NR
Hohmann 2019	9.1	NR	77.7	81	36.7	NR	NR	4.7	NR	NR	NR	NR	28.3	NR	NR	NR	47.1	NR	NR	NR	NR
Patti 2019	NR	NR	NR	NR	NR	NR	NR	NR	NR	NR	NR	NR	13	NR	NR	NR	NR	NR	NR	NR	NR

OSs: observational studies; AF: atrial fibrillation; DOACs: direct oral anticoagulants; VKA: vitamin k antagonist; Dabi: dabigatran; Riv: rivaroxaban; Api: apixaban; Edo: edoxaban; ACEI: Angiotensin-converting enzyme inhibitor; ARB: Angiotensin receptor inhibitor; Antip-drugs: Antiplatelet agents; Antia-drugs: Antiarrhythmic drugs; Asp: Aspirin; Amio: Amiodarone; CCB: calcium channel blocker; Clo: Clopidogrel; Dil: Diltiazem; Dron: Dronedarone; Estr: Estrogen; Glu: Glucocorticoids; H2: H2-receptor antagonist; NSAIDS: Non-steroidal anti-inflammatory drugs; PPI: Proton pump inhibitor; SSRI: Serotonin receptor antagonist; Vera: Verapamil; NR: not reported

Supplementary Table 6. Characteristics of RCTs

Study	Indication	NCT	Interventions	Numbers (>75years)	Controls	Numbers (>75years)	Follow-up
RE-LY 2009 (Connolly et al., 2009)	AF	NCT00262600	Dabigatran 110mg or 150mg twice	4815	Warfarin	2423	2years
ROCKET AF 2011 (Patel et al., 2011)	AF	NCT00403767	Rivaroxaban 20mg once	3073	Warfarin	3077	2.5years
ARISTOTLE 2011 (Granger et al., 2011)	AF	NCT00412984	Apixaban 5mg twice	2850	Warfarin	2828	1.8years
J-ROCKET AF 2012 (Hori et al., 2012)	AF	NCT00494871	Rivaroxaban 15mg once	251	Warfarin	245	30months
ENGAGE AF-TIMI 48 2013 (Giugliano et al., 2013)	AF	NCT00781391	Edoxaban 30mg or 60mg once	2838	Warfarin	2805	2.8years

AF: atrial fibrillation; NCT: national clinical trial; RCT: Randomized controlled trial

Supplementary Table 7. Patient demographics and clinical characteristics of RCTs

Study	Total number	Mean age (y)	Female (%)	Weight (kg)	BMI (kg/m^2)	HF (%)	HBP (%)	DM (%)	Stroke/ TIA (%)	MI (%)	Cancer (%)	Ccr (mL /min)	Ccr>80mL /min (%)	$CHADS_2$ score	Antiplatelet drugs
RE-LY 2009	18113	71.5	36.4	NR	NR	32	78.9	23.3	20	16.6	NR	NR	NR	2.1	40.1
ROCKET AF 2011	14236	73	39.7	NR	28.2	62.4	90.6	40	54.7	17.3	NR	67	NR	3.5	36.7
ARISTOTLE 2011	18140	70	35.3	82	NR	35.5	87.4	25	19.4	14.2	NR	NR	NR	2.1	NR
J-ROCKET AF 2012	1278	71.1	19.4	NR	NR	40.8	79.5	38	63.6	7.7	NR	NR	26.4	3.3	36.4
ENGAGE AF-TIMI 48 2013	21026	72	38.1	NR	NR	57.4	93.6	36.1	28.3	NR	NR	NR	19.3	2.8	29.3

BMI: Body Mass Index; Ccr: creatinine clearance rate; DM: Diabetes; HF: heart failure; HBP: hypertension; NR: not reported; TIA: transient ischemic attack

Supplementary Table 8. Quality assessment of OSs

Study	Selection bias	Bias due to residual confounding	Bias due to time-varying covariates/information censoring	Bias due to selective reporting of study outcomes
Lindsay 2014	Low	Moderate	Moderate	Low
Graham 2014	Low	Low	Low	Low
Hernandez 2015	Low	Low	Low	Low
Abraham 2015	Low	Low	Low	Low
Seeger 2015	Low	Low	Moderate	Low
Maura 2015	Low	Low	Low	Low
Lauffenburger 2015	Low	Low	Moderate	Low
Avgil-Tsadok 2016	Low	Low	Low	Low
Lau 2017	Low	Low	Moderate	Low
Go 2017	Low	Low	Moderate	Low
Nielsen 2017	Low	Low	Moderate	Low
Bengtson 2017	Low	Moderate	Moderate	Low
Norby 2017	Low	Low	Moderate	Low
Cha 2017	Low	Moderate	Moderate	Low
Friberg 2017	Low	Moderate	Moderate	Low
Adeboyeje 2017	Low	Moderate	Moderate	Low
Forslund 2018	Low	Low	Moderate	Low

Chao 2018	Low	Moderate	Moderate	Low
Ujeyl 2018	Low	Moderate	Moderate	Low
Lip 2018	Low	Low	Moderate	Low
Zoppellaro 2018	Low	Moderate	Low	Low
Lai 2018	Low	Moderate	Moderate	Low
Lee 2018	Low	Low	Moderate	Low
Hohmann 2019	Low	Moderate	Moderate	Low
Patti 2019	Low	Moderate	Moderate	Low
Giustozzi 2019	Low	Moderate	Moderate	Low
Chan 2019	Low	Moderate	Moderate	Low

OSs: observational studies; Low: low risk; Moderate: moderate risk: unclear risk; High: high risk.

Supplementary Table 9. Quality assessment of RCTs

Study	Random sequence generation	Allocation concealment	Blinding of participants and personnel	Blinding of outcome assessment	Incomplete outcome data	Selective reporting	Other bias
RE-LY 2009	Low	Low	High	Low	Low	Low	Low
ROCKET AF 2011	Low	Low	Low	Low	Low	Unclear	Low
ARISTOTLE 2011	Low	Low	Low	Low	Low	Unclear	Low
J-ROCKET AF 2012	Unclear	Unclear	Unclear	Unclear	Low	Low	Low
ENGAGE AF-TIMI 48 2013	Low	Low	Low	Low	Low	Low	Low

AF: atrial fibrillation; RCT: Randomized controlled trial; Low: low risk; Moderate: moderate risk: unclear risk; High: high risk.

Supplementary Table 10. The comparability between primacy analysis and subgroup analysis in OSs

**A** Stroke/SE	No.s	*P* for interaction (Overall vs. Sub)
**Gender**		
Men	2	0.09
Women	2	0.41
**Age**		
>80years	6	0.89
>85years	4	0.59
>90years	2	0.72
**Population**		
USA	7	0.07
Canada	1	0.01
Italy	2	0.72
Germany	2	0.01
Sweden	1	0.07
Danish	1	0.37
France	NA	
Spain	NA	
Korea	2	0.78
Taiwan	3	0.02
Hong Kong	NA	

**B** ICH	No.s	*P* for interaction (Overall vs. Sub)
**Gender**		
Men	1	0.08
Women	1	0.69
**Age**		
>80years	4	0.16
>85years	4	0.16
>90years	1	0.51
**Population**		
USA	3	0.82
Canada	1	0.22
Italy	1	0.29
Germany	1	0.15
Sweden	1	0.11
Danish	NA	
France	NA	
Spain	1	0.00
Korea	2	0.85
Taiwan	3	0.40
Hong Kong	NA	

**C** Major bleeding	No.s	*P* for interaction (Overall vs. Sub)
**Gender**		
Men	NA	
Women	NA	
**Age**		
>80years	9	0.42
>85years	5	0.01
>90years	2	0.42
**Population**		
USA	4	0.76
Canada	1	0.01
Italy	2	0.16
Germany	1	0.07
Sweden	1	0.23
Danish	1	0.30
France	1	0.76
Spain	1	0.00
Korea	1	0.11
Taiwan	1	0.00
Hong Kong	1	0.17

**D** GI bleeding	No.s	*P* for interaction (Overall vs. Sub)
**Gender**		
Men	1	0.96
Women	1	0.11
**Age**		
>80years	5	0.28
>85years	3	0.38
>90years	NA	
**Population**		
USA	4	0.07
Canada	1	0.54
Italy	1	0.16
Germany	1	0.45
Sweden	1	0.76
Danish	NA	
France	NA	
Spain	1	0.00
Korea	1	0.09
Taiwan	2	0.00
Hong Kong	NA	

**E** All-cause mortality	No.s	*P* for interaction (Overall vs. Sub)
**Gender**		
Men	2	0.75
Women	2	0.31
**Age**		
>80years	6	0.58
>85years	3	0.77
>90years	NA	
**Population**		
USA	2	0.29
Canada	NA	
Italy	1	0.06
Germany	1	0.05
Sweden	2	0.12
Danish	1	0.10
France	NA	
Spain	NA	
Korea	2	0.00
Taiwan	1	0.00
Hong Kong	NA	

**F** MI	No.s	*P* for interaction (Overall vs. Sub)
**Gender**		
Men	NA	
Women	NA	
**Age**		
>80years	3	0.79
>85years	2	0.83
>90years	NA	
**Population**		
USA	4	0.61
Canada	NA	
Italy	1	0.62
Germany	NA	
Sweden	NA	
Danish	NA	
France	NA	
Spain	NA	
Korea	NA	
Taiwan	2	0.08
Hong Kong	NA	

Supplementary Table 11. Sensitivity analysis of Stroke/SE in OSs

**Study omitted**	**HR (95%CI)**
Hernandez 2015	0.85(0.76-0.95)
Maura 2015	0.90(0.78-1.02)
Seeger 2015	0.92(0.79-1.04)
Lauffenburger 2015	0.91(0.78-1.04)
Lau 2017	0.89(0.78-1.01)
Go 2017	0.88(0.77-0.99)
Nielsen 2017	0.90(0.78-1.02)
Adeboyeje 2017	0.91(0.79-1.02)
Friberg 2017	0.90(0.78-1.02)
Forslund 2018	0.89(0.77-1.01)
Chao 2018	0.90(0.78-1.02)
Lip 2018	0.91(0.79-1.02)
Zoppellaro 2018	0.89(0.78-1.01)
Lee 2018	0.92(0.80-1.03)
Hohmann 2019	0.92(0.80-1.04)
Patti 2019	0.93(0.81-1.04)
Giustozzi 2019	0.90 (0.78-1.00)
Chan 2019	0.93(0.82-1.04)

Supplementary Table 12. Sensitivity analysis of ICH in OSs

**Study omitted**	**HR (95%CI)**
Graham 2014	0.47(0.33-0.62)
Hernandez 2015	0.48(0.39-0.58)
Seeger 2015	0.47(0.33-0.61)
Avgil-Tsadok 2016	0.44(0.31-0.58)
Go 2017	0.44(0.31-0.58)
Norby 2017	0.45(0.32-0.58)
Cha 2017	0.44(0.31-0.58)
Forslund 2018	0.43(0.30-0.57)
Chao 2018	0.46(0.33-0.60)
Zoppellaro 2018	0.44(0.31-0.58)
Lai 2018	0.46(0.33-0.60)
Lee 2018	0.47(0.34-0.61)
Hohmann 2019	0.44(0.31-0.57)
Chan 2019	0.46(0.32-0.61)

OSs: observational studies; ICH: intracranial hemorrhage

Supplementary Table 13. Sensitivity analysis of major bleeding in OSs

**Study omitted**	**HR (95%CI)**
Hernandez 2015	0.85(0.76-0.95)
Maura 2015	0.90(0.78-1.02)
Seeger 2015	0.92(0.79-1.04)
Lauffenburger 2015	0.91(0.78-1.04)
Lau 2017	0.89(0.78-1.01)
Go 2017	0.88(0.77-0.99)
Nielsen 2017	0.90(0.78-1.02)
Adeboyeje 2017	0.91(0.79-1.02)
Friberg 2017	0.90(0.78-1.02)
Forslund 2018	0.89(0.77-1.01)
Chao 2018	0.90(0.78-1.02)
Lip 2018	0.91(0.79-1.02)
Zoppellaro 2018	0.89(0.78-1.01)
Lee 2018	0.92(0.80-1.03)
Hohmann 2019	0.92(0.80-1.04)
Patti 2019	0.93(0.81-1.04)
Giustozzi 2019	0.90(0.78-1.00)
Chan 2019	0.93(0.82-1.04)

Supplementary Table 14. Sensitivity analysis of GIB in OSs

**Study omitted**	**HR (95%CI)**
Graham 2014	1.16(0.92-1.40)
Hernandez 2015	1.11(0.91-1.31)
Abrraham 2015	1.18(0.94-1.41)
Seeger 2015	1.21(0.95-1.47)
Avgil-Tsadok 2016	1.17(0.93-1.42)
Go 2017	1.16(0.92-1.40)
Bengtson 2017	1.16(0.93-1.40)
Norby 2017	1.17(0.93-1.41)
Forslund 2018	1.18(0.93-1.42)
Zoppellaro 2018	1.15(0.92-1.39)
Lai 2018	1.21(0.96-1.45)
Lee 2018	1.21(0.97-1.45)
Hohmann 2019	1.20(0.93-1.47)
Patti 2019	1.23(1.01-1.47)
Chan 2019	1.24(1.03-1.45)

OSs: observational studies; GIB: gastrointestinal bleeding

Supplementary Table 15. Sensitivity analysis of all-cause mortality in OSs

**Study omitted**	**HR (95%CI)**
Linsay 2014	0.89(0.74-1.03)
Graham 2014	0.90(0.74-1.05)
Nielsen 2017	0.86(0.72-1.01)
Friberg 2017	0.90(0.73-1.07)
Forslund 2018	0.88(0.73-1.04)
Ujeyl 2018	0.85(0.74-0.96)
Zoppellaro 2018	0.90(0.74-1.06)
Lai 2018	0.93(0.80-1.05)
Lee 2018	0.91(0.76-1.04)

OSs: observational studies

Supplementary Table 16. Sensitivity analysis of MI in OSs

**Study omitted**	**HR (95%CI)**
Lauffenburger 2015	0.92(0.76-1.08)
Go 2017	0.92 (0.81-1.04)
Bengtson 2017	0.92(0.78-1.07)
Norby 2017	0.89 (0.72-1.06)
Zoppellaro 2018	0.87 (0.72-1.02)
Lai 2018	0.92(0.79-1.06)
Chan 2019	0.99(0.91-1.07)

OSs: observational studies; MI: myocardial infarction

Supplementary Table 17. Meta-regression analysis of Stroke/SE in OSs

**Variable**	**P value for OSs**
Mean age (y)	0.842
Female (%)	0.670
Obesity (%)	0.764
HF (%)	0.958
HBP (%)	0.666
DM	0.438
Stroke/TIA (%)	0.427
MI (%)	0.621
Renal disease (%)	0.883
Liver disease (%)	0.932
Anemia (%)	0.757
Cancer (%)	0739
$CHADS_2$ (mean)	0.773
CHADS2-VASc (mean)	0.963
HAS-BLED (mean)	0.852
HAS-BLED>3 (%)	0.769
Prior bleeding	0.634
Prior GI bleeding	0.768

ACEI/ARB	0.689
Beta-blocker	0.615
Dil	0.503
CCB	0.995
Amio	0.735
Dron	0.588
Digoxin	0.610
Antia-drugs	0.913
Statin	0.888
Antip-drugs	0.460
Asp	0.537
Clo	0.869
NSAIDS	0.588
PPI	0.494
H2	0.383
SSRI	0.606

OSs: observational studies; SE: systemic embolism

Supplementary Table 18. Meta-regression analysis of ICH in OSs

**Variable**	**P value for OSs**
Mean age (y)	0.572
Female (%)	0.490
Obesity(%)	0.654
HF (%)	0.664
HBP (%)	0.749
DM	0.583
Stroke/TIA (%)	0.778
MI (%)	0.604
Renal disease (%)	0.759
Liver disease (%)	0.717
Anemia (%)	0.636
Cancer (%)	0.434
$CHADS_2$ (mean)	0.507
CHADS2-VASc (mean)	0.515
HAS-BLED (mean)	0.586
HAS-BLED>3 (%)	0.507
Prior bleeding	0.509
Prior GI bleeding	0.765

ACEI/ARB	0.503
Beta-blocker	0.769
Dil	0.456
CCB	0.882
Amio	0.394
Dron	0.622
Digoxin	0.578
Antia-drugs	0.833
Statin	0.667
Antip-drugs	0.863
Asp	0.983
Clo	0.992
NSAIDS	0.894
PPI	0.609
H2	0.617
SSRI	0.348

OSs: observational studies; ICH: intracranial hemorrhage

Supplementary Table 19. Meta-regression analysis of major bleeding in OSs

**Variable**	**P value for OSs**
Mean age (y)	0.839
Female (%)	0.775
Obesity(%)	0.653
HF (%)	0.882
HBP (%)	0.770
DM	0.967
Stroke/TIA (%)	0.983
MI (%)	0.883
Renal disease (%)	0.866
Liver disease (%)	0.685
Anemia (%)	0.723
Cancer (%)	0.952
$CHADS_2$ (mean)	0.841
CHADS2-VASc (mean)	0.624
HAS-BLED (mean)	0.766
HAS-BLED>3 (%)	0.487
Prior bleeding	0.812
Prior GI bleeding	0.892

ACEI/ARB	0.876
Beta-blocker	0.587
Dil	0.752
CCB	0.789
Amio	0.785
Dron	0.649
Digoxin	0.843
Antia-drugs	0.923
Statin	0.599
Antip-drugs	0.569
Asp	0.755
Clo	0.612
NSAIDS	0.519
PPI	0.634
H2	0.664
SSRI	0.524

OSs: observational studies

Supplementary Table 20. Meta-regression analysis of GIB in OSs

**Variable**	**P value for OSs**
Mean age (y)	0.575
Female (%)	0.678
Obesity (%)	0.653
HF (%)	0.923
HBP (%)	0.497
DM	0.386
Stroke/TIA (%)	0.626
MI (%)	0.700
Renal disease (%)	0.459
Liver disease (%)	0.734
Anemia (%)	0.948
Cancer (%)	0.514
$CHADS_2$ (mean)	0.815
CHADS2-VASc (mean)	0.966
HAS-BLED (mean)	0.688
HAS-BLED>3 (%)	0.723
Prior bleeding	0.571
Prior GI bleeding	0.564

ACEI/ARB	0.432
Beta-blocker	0.532
Dil	0.525
CCB	0.730
Amio	0.434
Dron	0.817
Digoxin	0.549
Antia-drugs	0.966
Statin	0.803
Antip-drugs	0.803
Asp	0.844
Clo	0.995
NSAIDS	0.940
PPI	0.933
H2	0.862
SSRI	0.873

OSs: observational studies; GIB: gastrointestinal bleeding

Supplementary Table 21. Meta-regression analysis of all cause mortality in OSs

**Variable**	**P value for OSs**
Mean age (y)	0.800
Female (%)	0.403
Obesity (%)	0.562
HF (%)	0.942
HBP (%)	0.392
DM	0.264
Stroke/TIA (%)	0.467
MI (%)	0.998
Renal disease (%)	0.507
Liver disease (%)	0.302
Anemia (%)	0.910
Cancer (%)	0.924
$CHADS_2$ (mean)	0.858
CHADS2-VASc (mean)	0.682
HAS-BLED (mean)	0.540
HAS-BLED>3 (%)	0.854
Prior bleeding	0.249
Prior GI bleeding	0.742

ACEI/ARB	0.652
Beta-blocker	0.614
Dil	0.858
CCB	0.620
Amio	0.847
Dron	0.724
Digoxin	0.645
Antia-drugs	0.903
Statin	0.723
Antip-drugs	0.889
Asp	0.379
Clo	0.835
NSAIDS	0.652
PPI	0.593
H2	0.534
SSRI	0.578

Supplementary Table 22. Meta-regression analysis of MI in OSs

**Variable**	**P value for OSs**
Mean age (y)	0.417
Female (%)	0.331
Obesity (%)	0.825
HF (%)	0.686
HBP (%)	0.998
DM	0.547
Stroke/TIA (%)	0.446
MI (%)	0.717
Renal disease (%)	0.485
Liver disease (%)	0.481
Anemia (%)	0.637
Cancer (%)	0.807
$CHADS_2$ (mean)	0.939
CHADS2-VASc (mean)	0.553
HAS-BLED (mean)	0.437
HAS-BLED>3 (%)	0.823
Prior bleeding	0.864
Prior GI bleeding	0.750

ACEI/ARB	0.624
Beta-blocker	0.813
Dil	0.860
CCB	0.560
Amio	0.664
Dron	0.531
Digoxin	0.428
Antia-drugs	0.545
Statin	0.870
Antip-drugs	0.989
Asp	0.742
Clo	0.448
NSAIDS	0.451
PPI	0.622
H2	0.619
SSRI	0.427

OSs: observational studies; MI: myocardial infarction

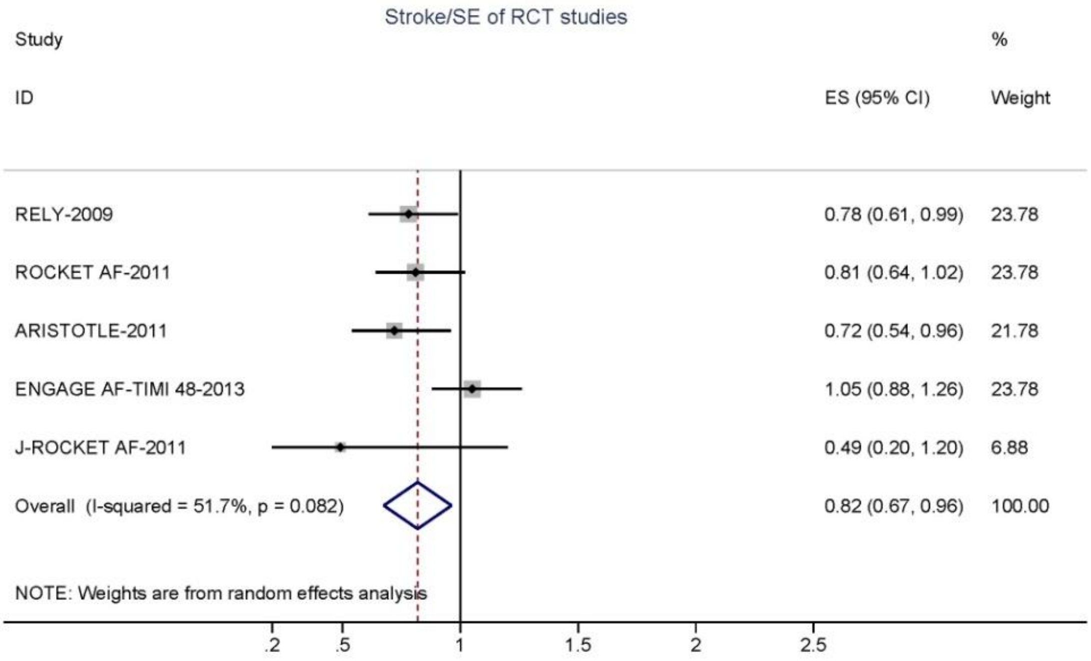

Supplementary Figure 1. Stroke/SE of RCTs

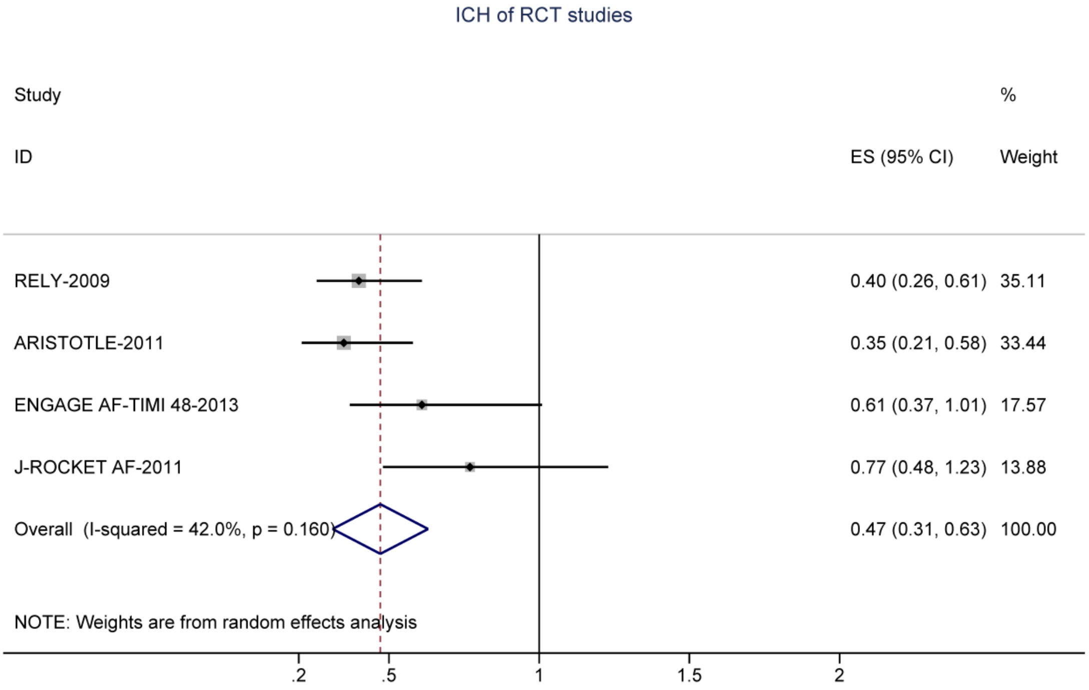

Supplementary Figure 2. ICH of RCTs

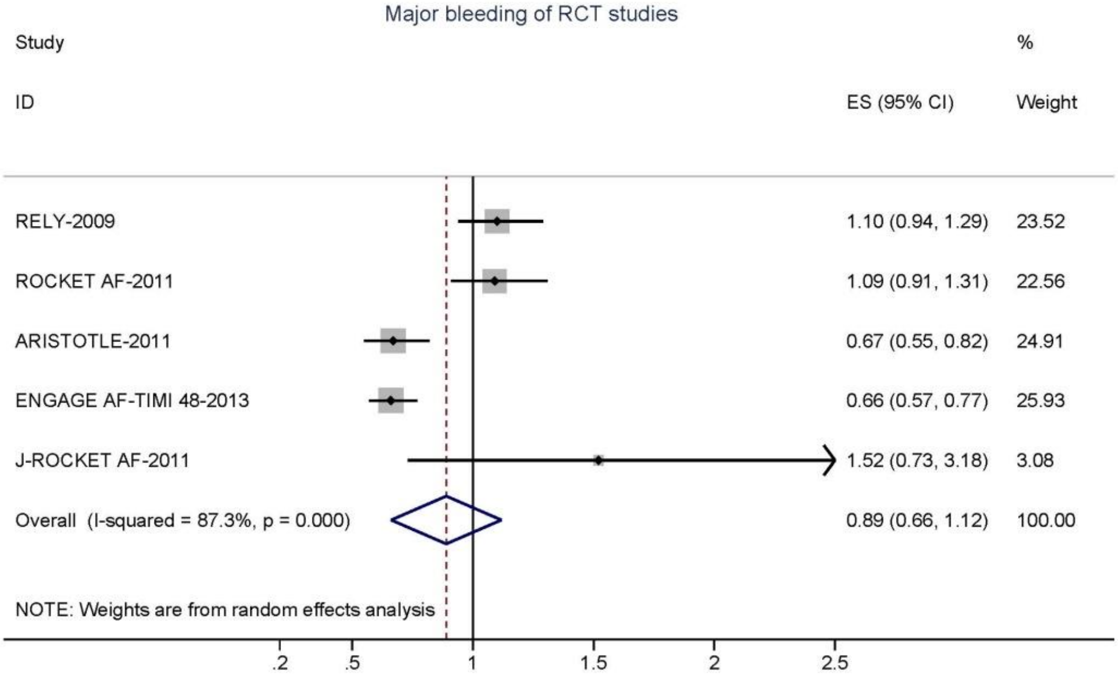

Supplementary Figure 3. Major bleeding of RCTs

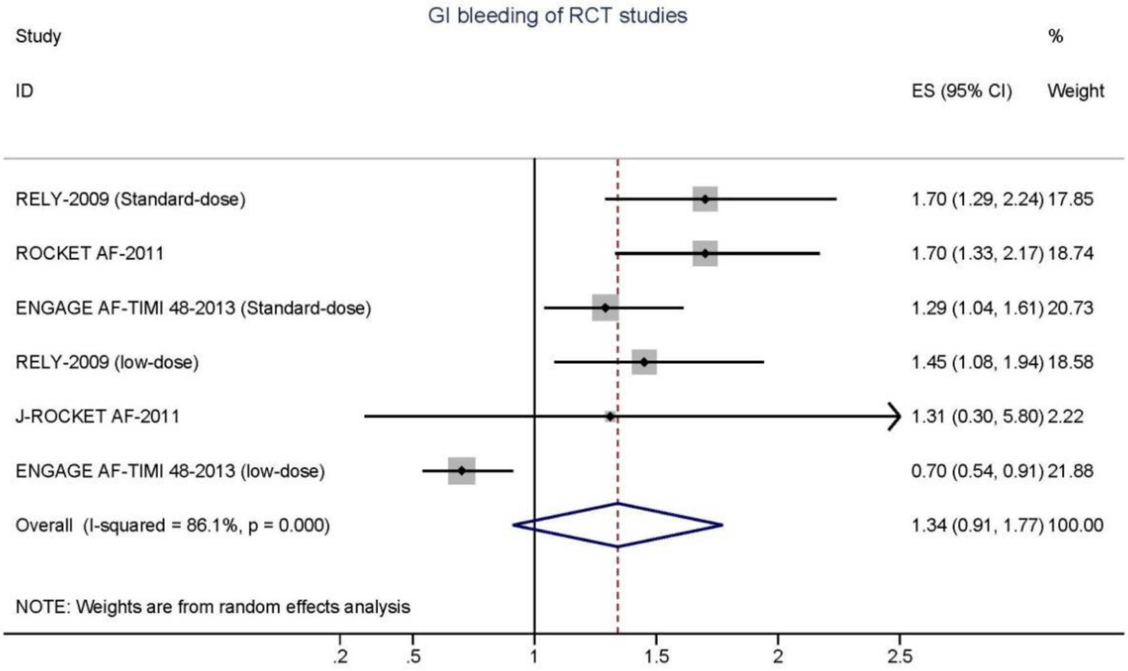

Supplementary Figure 4. GI bleeding of RCTs

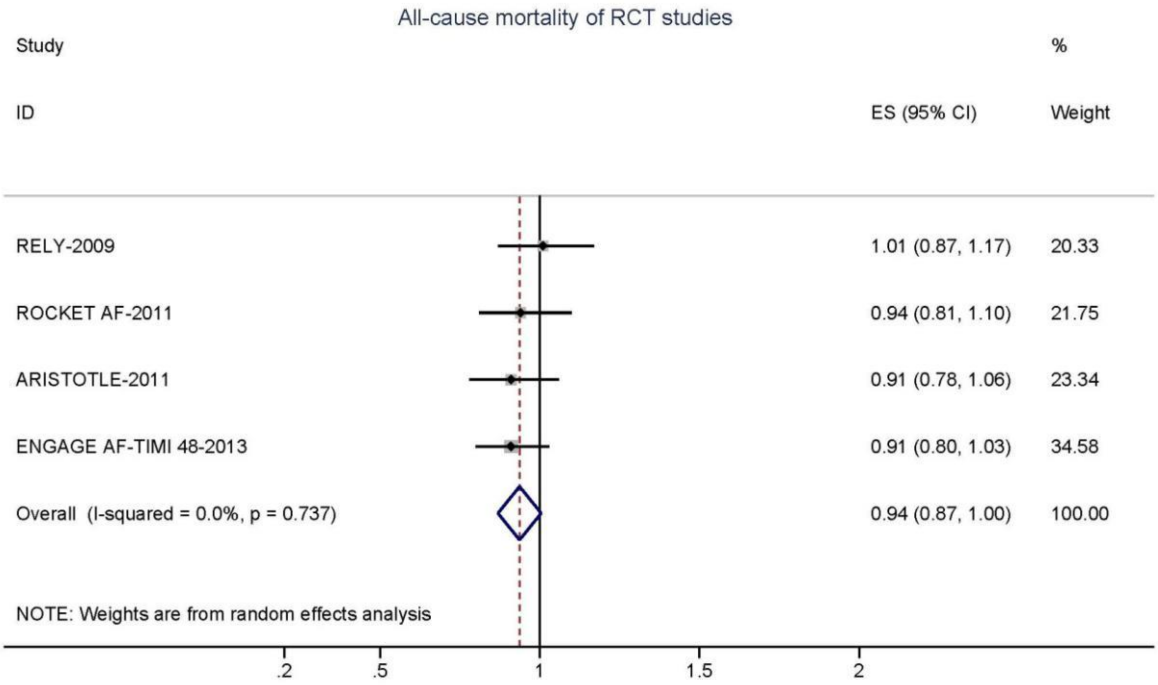

Supplementary Figure 5. All-cause mortality of RCTs

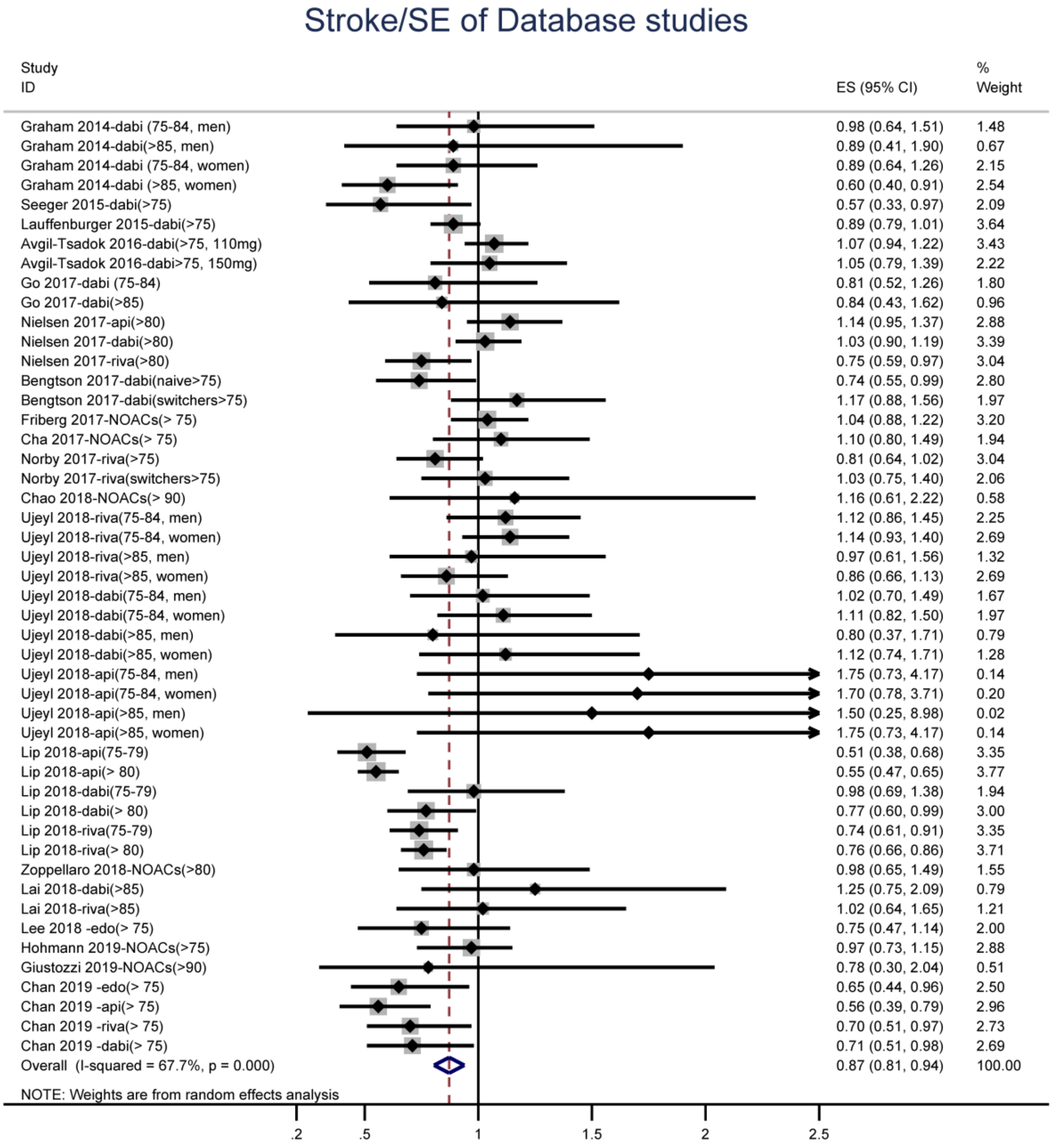

Supplementary Figure 6. Stroke/SE of OSs

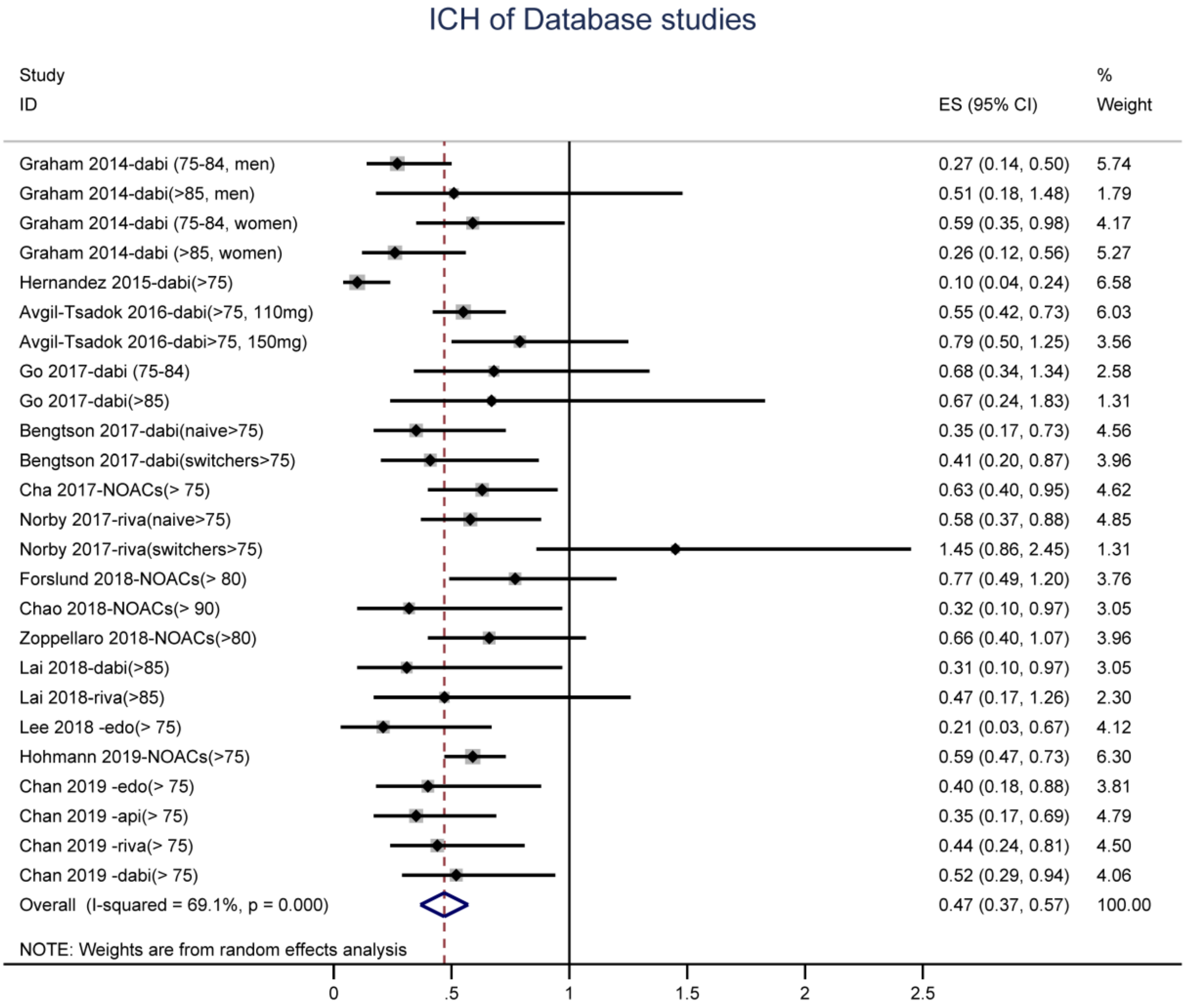

Supplementary Figure 7. ICH of OSs

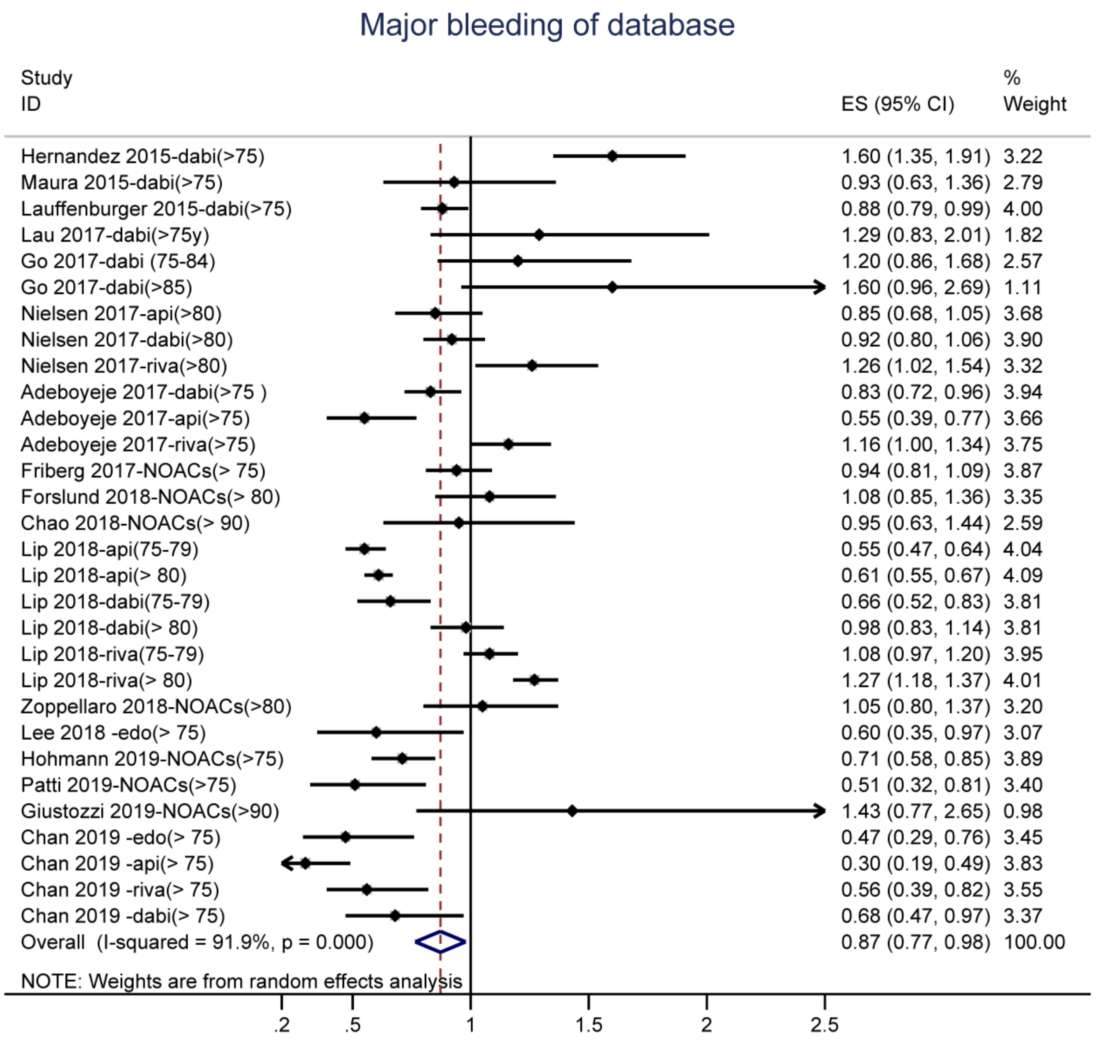

Supplementary Figure 8. Major bleeding of OSs

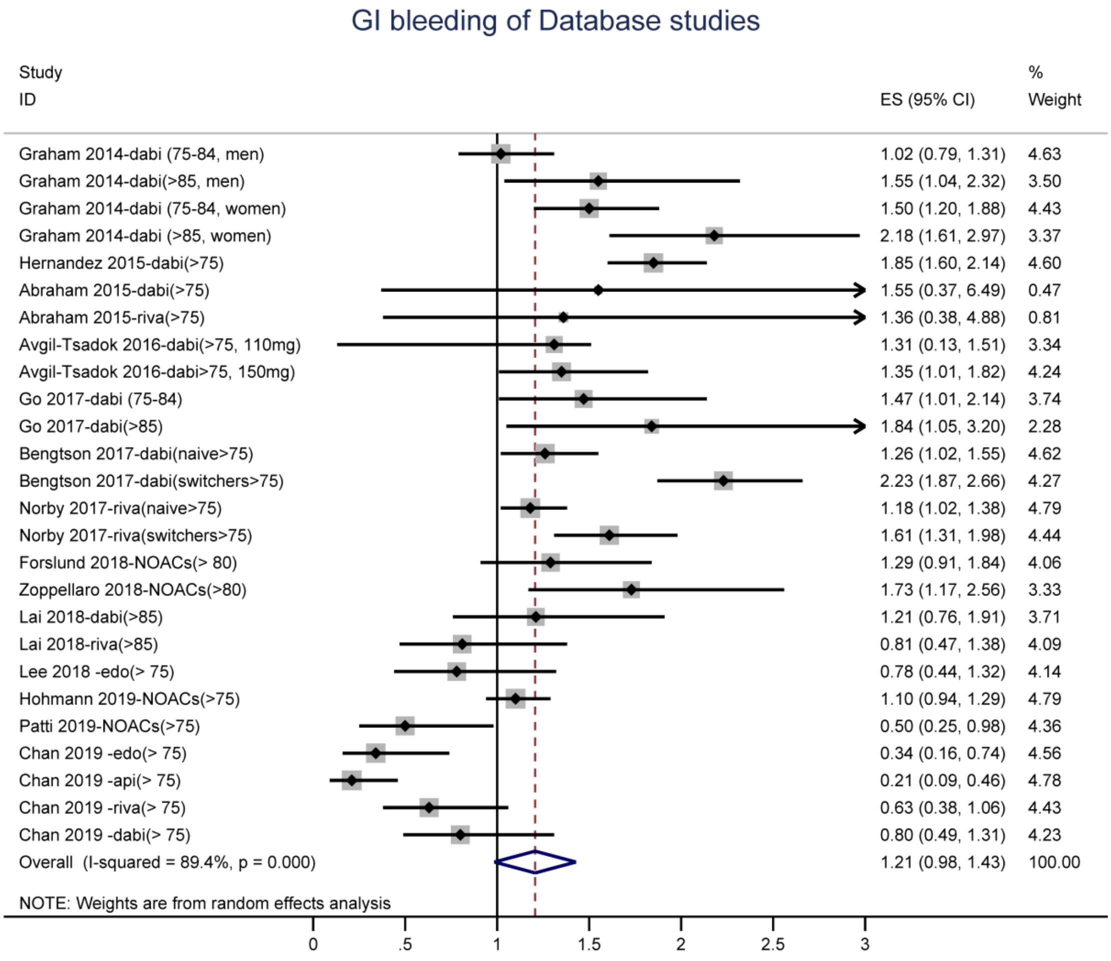

Supplementary Figure 9. GI bleeding of OSs

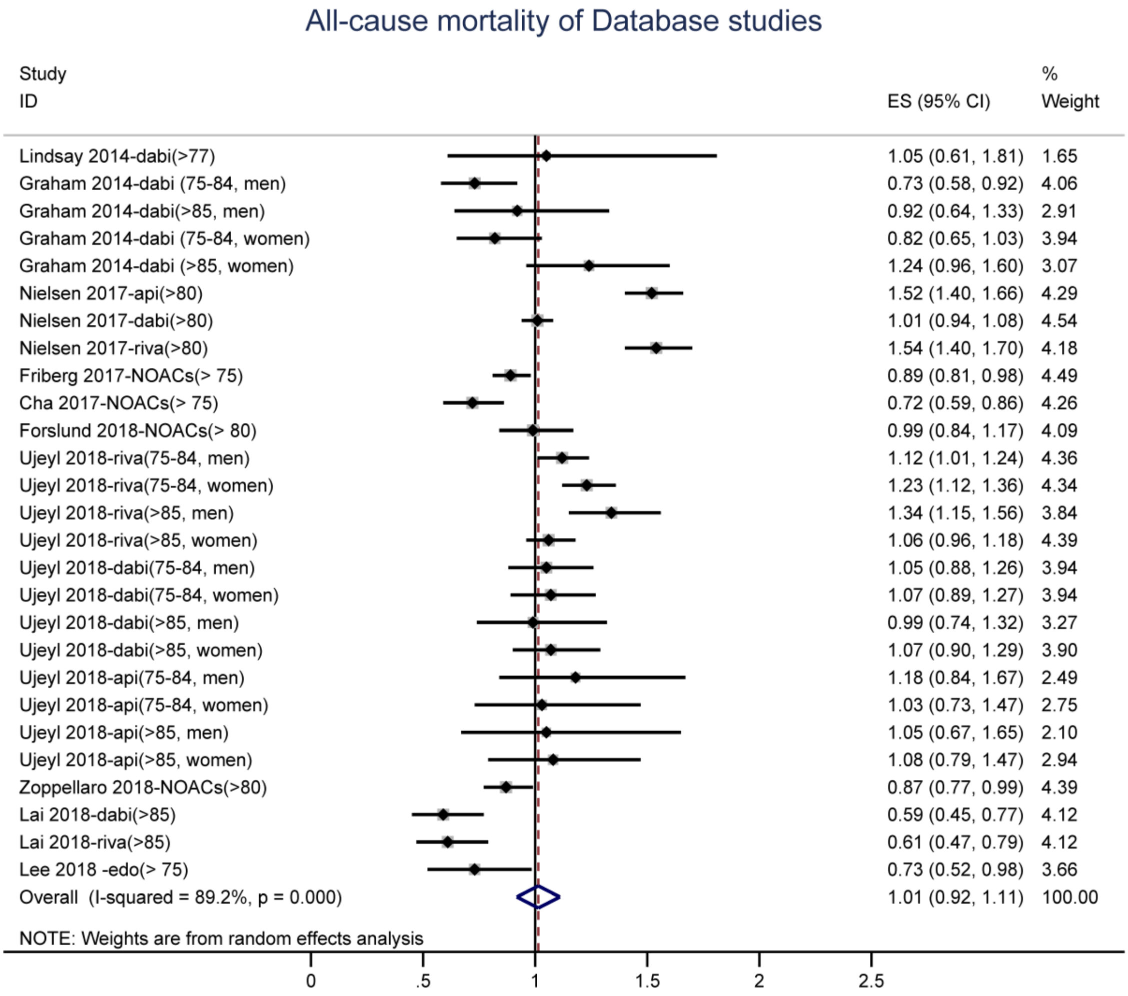

Supplementary Figure 10. All-cause mortality of OSs

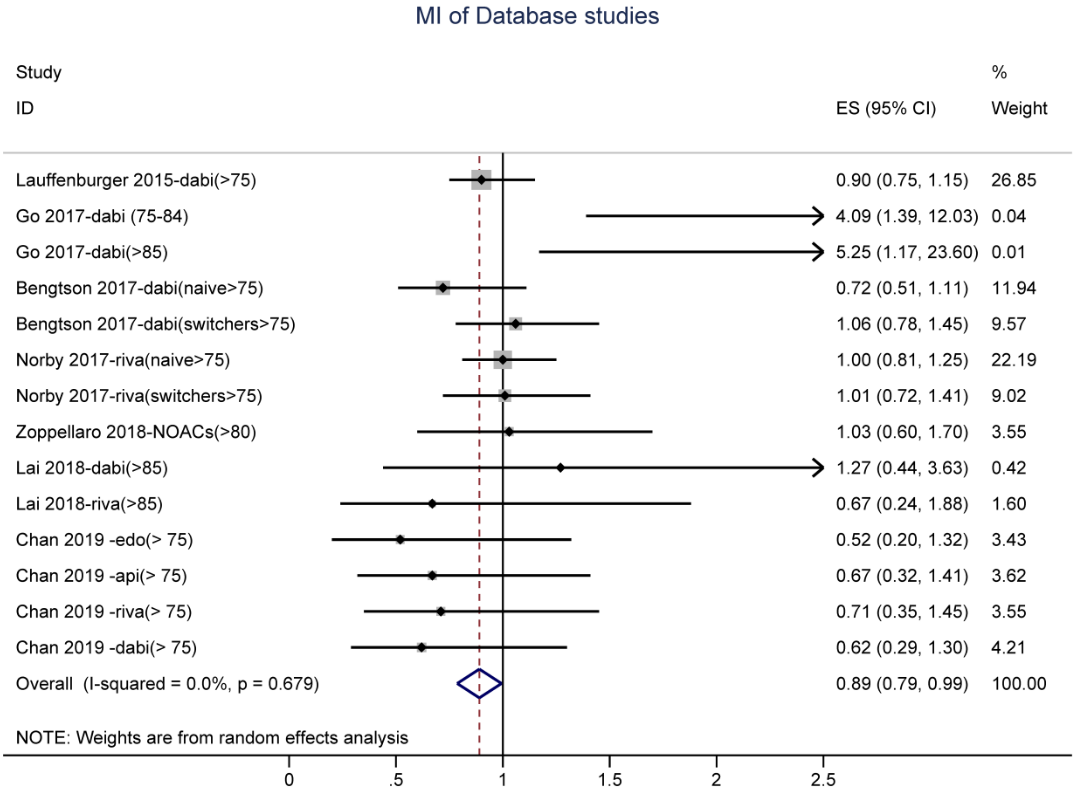

Supplementary Figure 11. MI of OSs

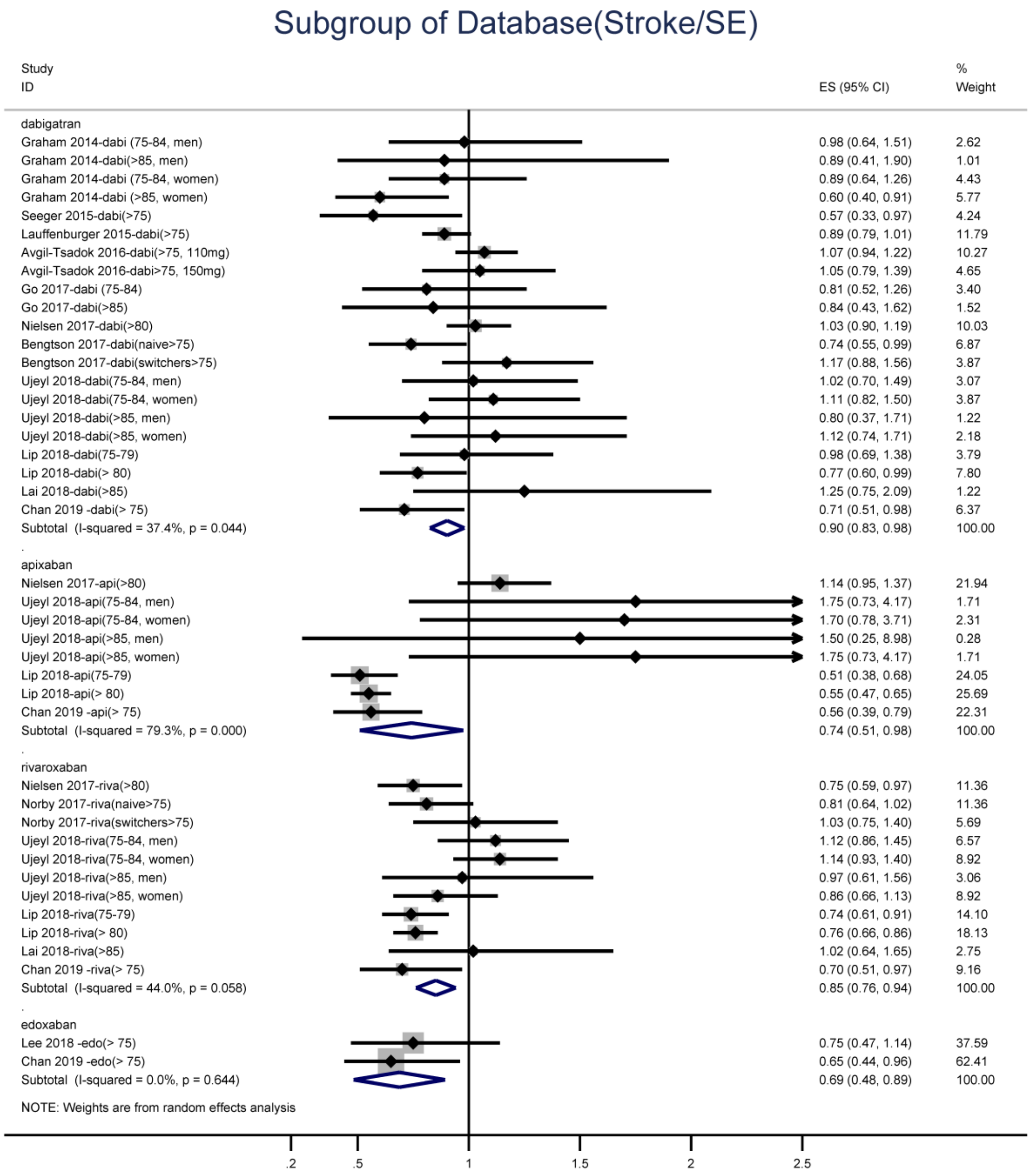

Supplementary Figure 12. Stroke/SE in rivaroxaban/dabigatran/apixaban/edoxaban (OSs)

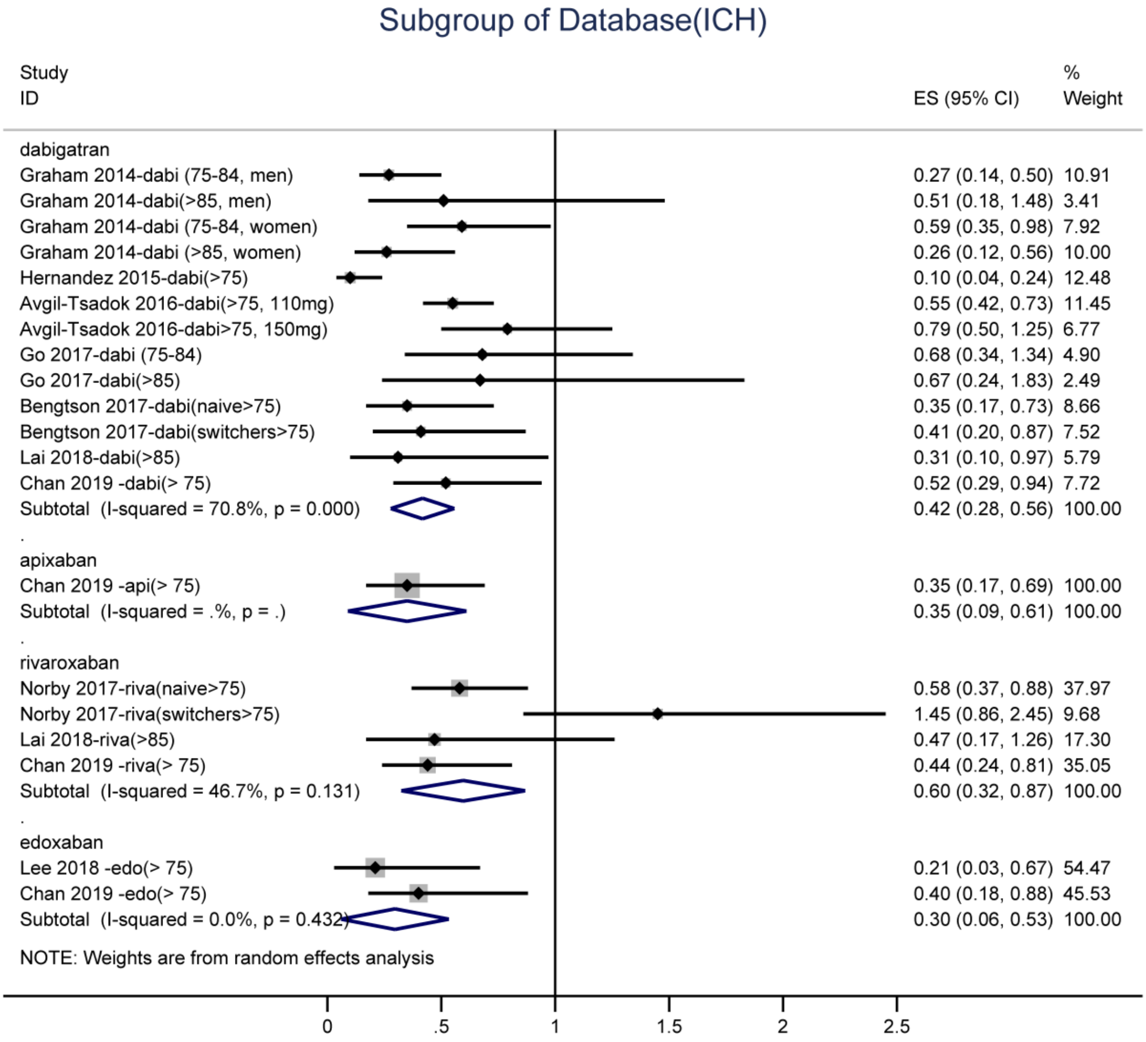

Supplementary Figure 13. ICH in rivaroxaban/dabigatran/apixaban/edoxaban (OSs)

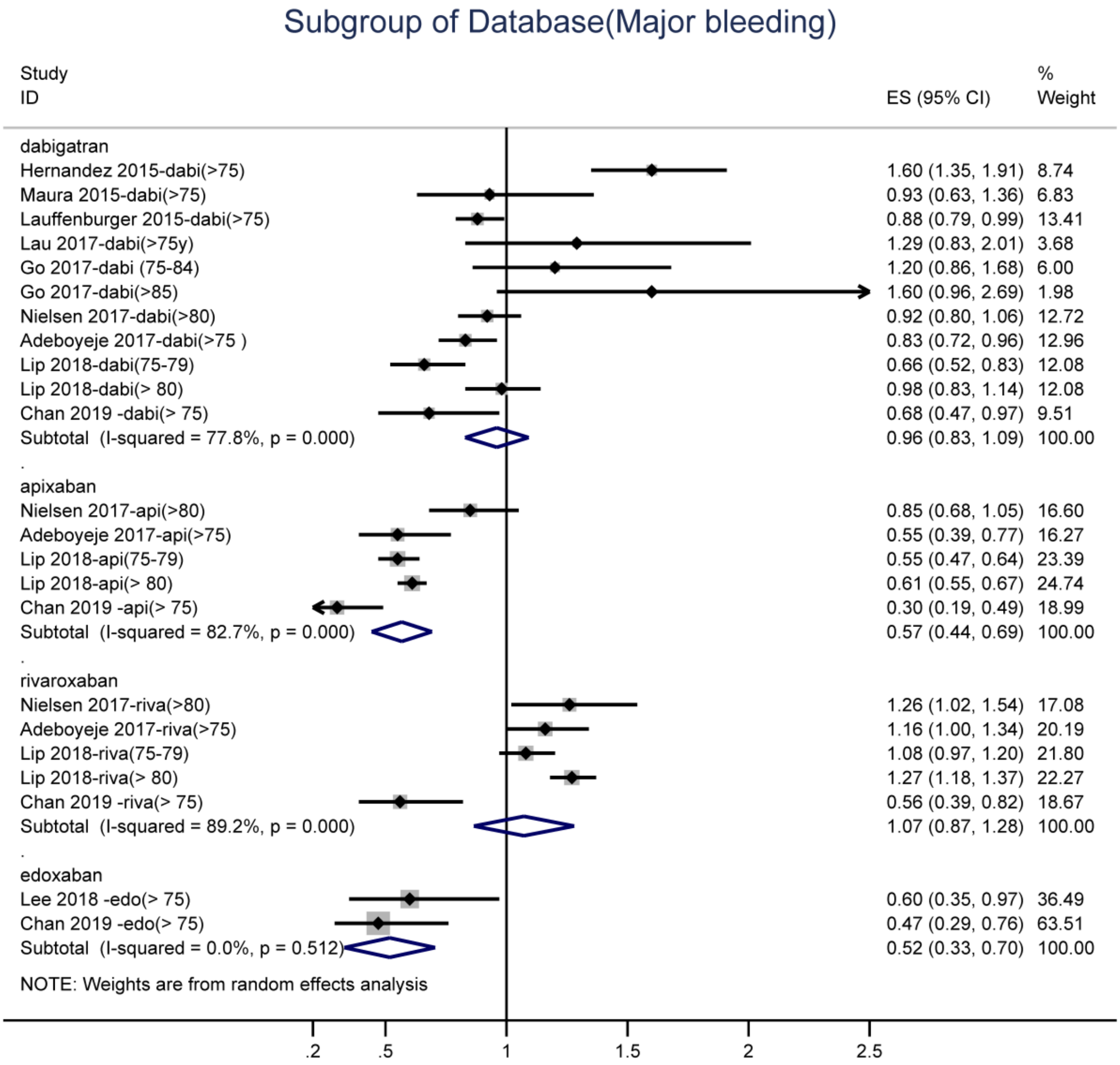

Supplementary Figure 14. Major bleeding in rivaroxaban/dabigatran/apixaban/edoxaban (OSs)

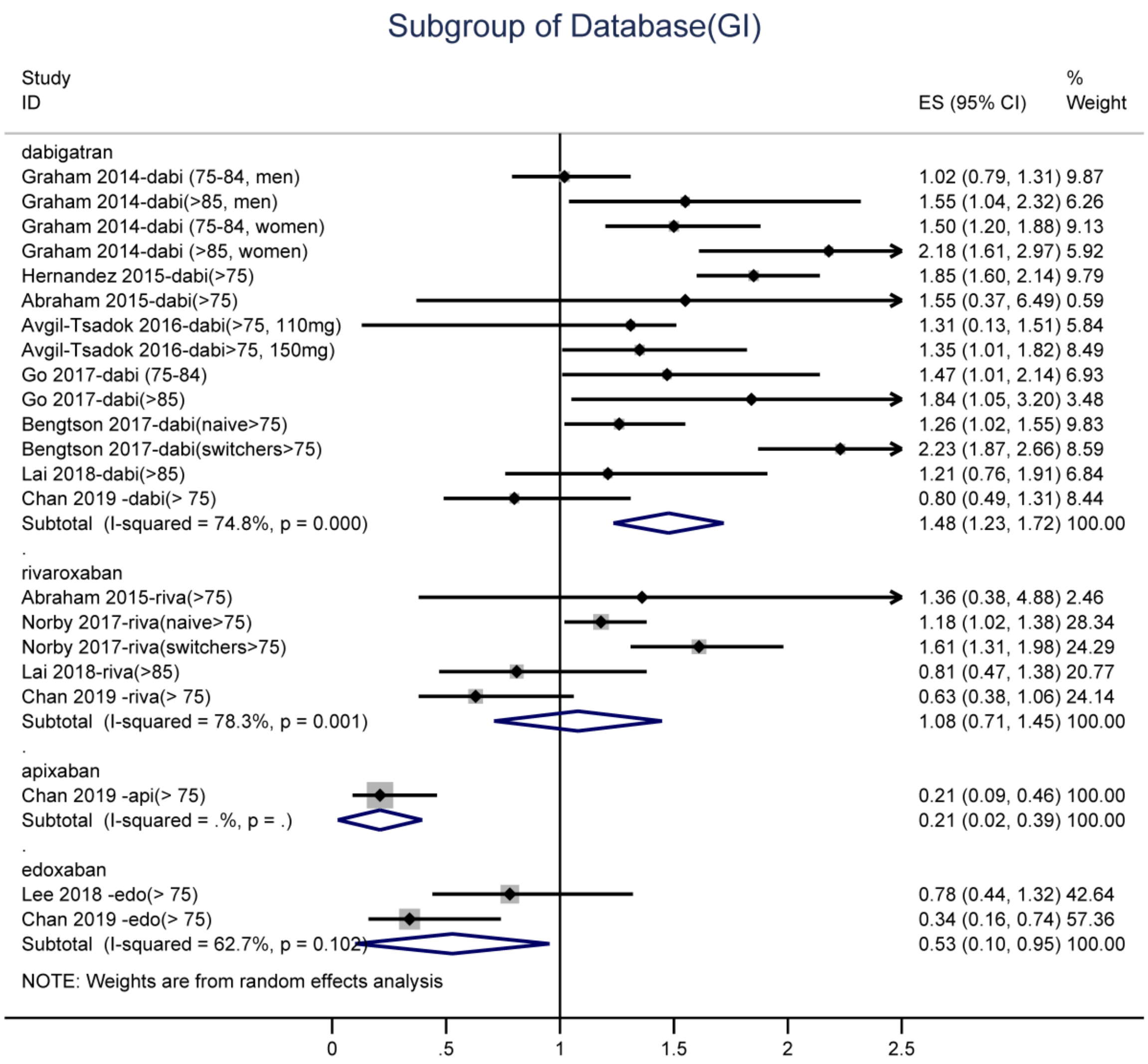

Supplementary Figure 15. GI bleeding in rivaroxaban/dabigatran/apixaban/edoxaban (OSs)

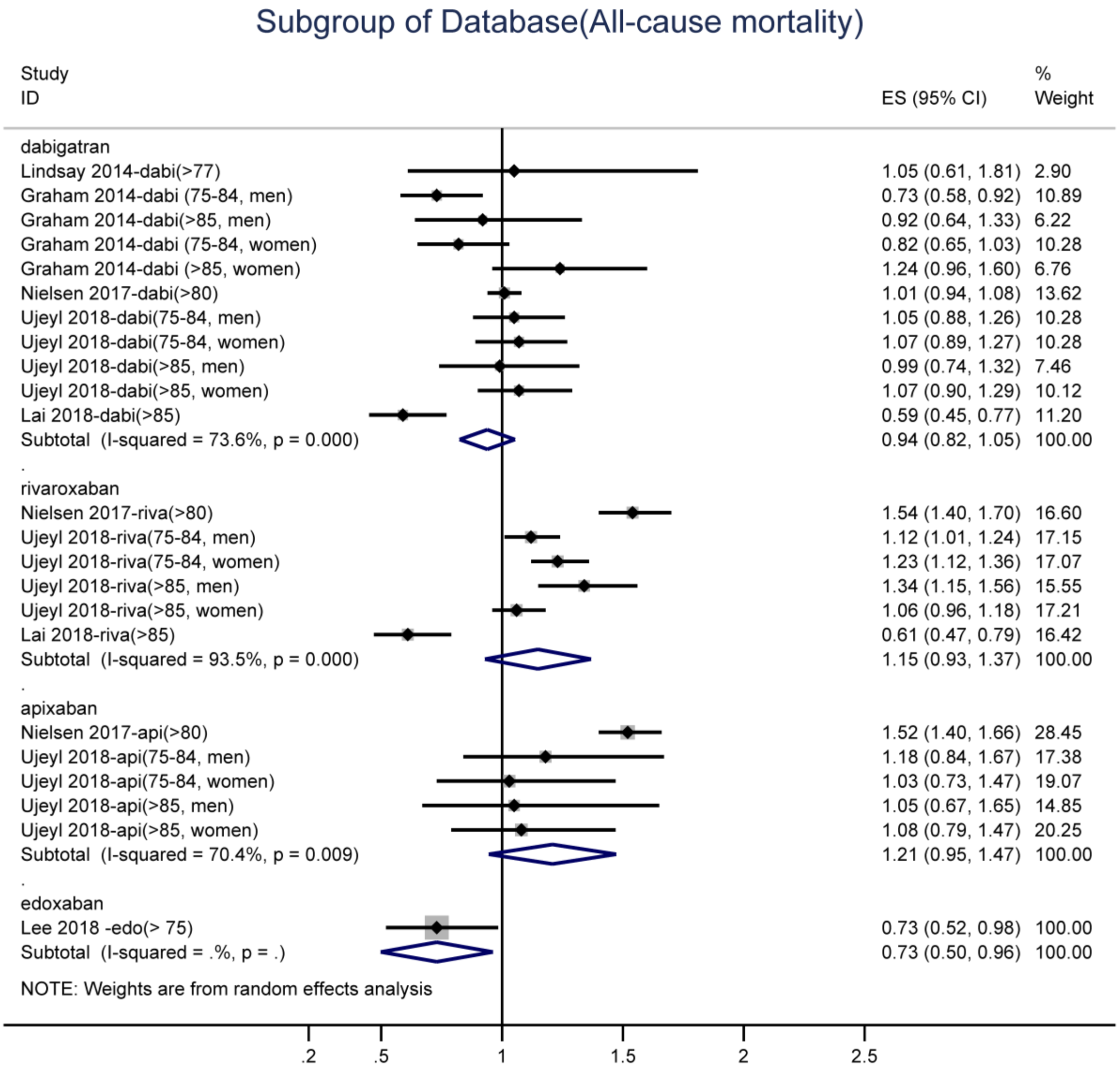

Supplementary Figure 16. All-cause mortality in rivaroxaban/dabigatran/apixaban/edoxaban (OSs)

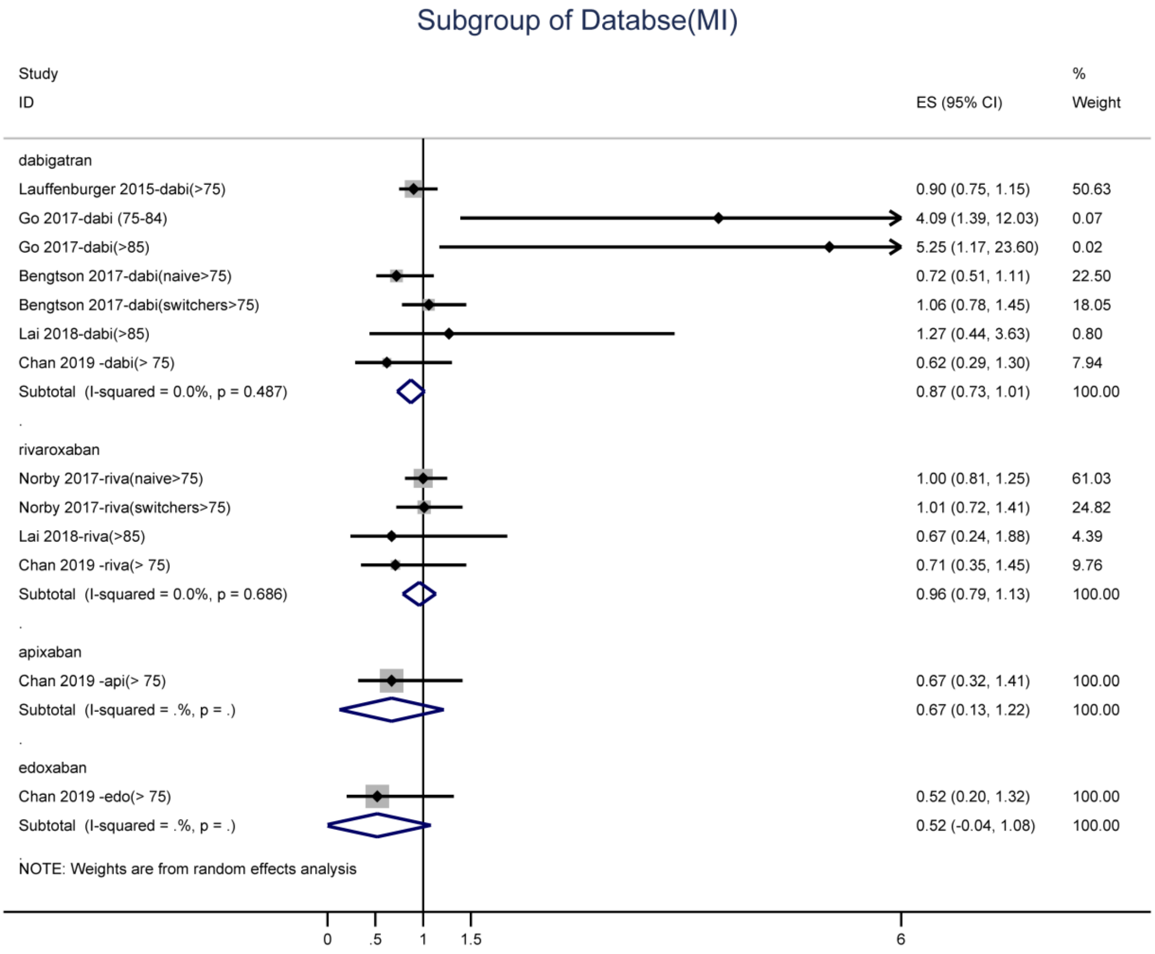

Supplementary Figure 17. MI in rivaroxaban/dabigatran/apixaban/edoxaban (OSs)

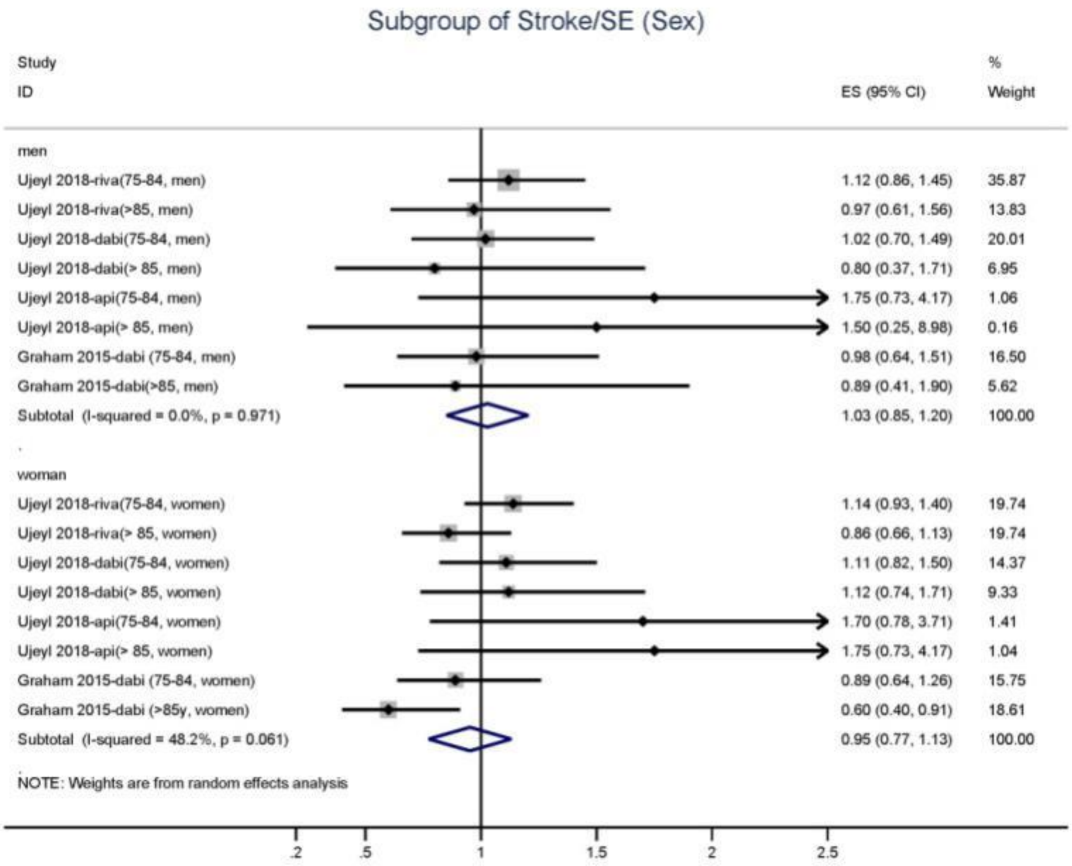

Supplementary Figure 18. Stroke/SE by gender of OSs

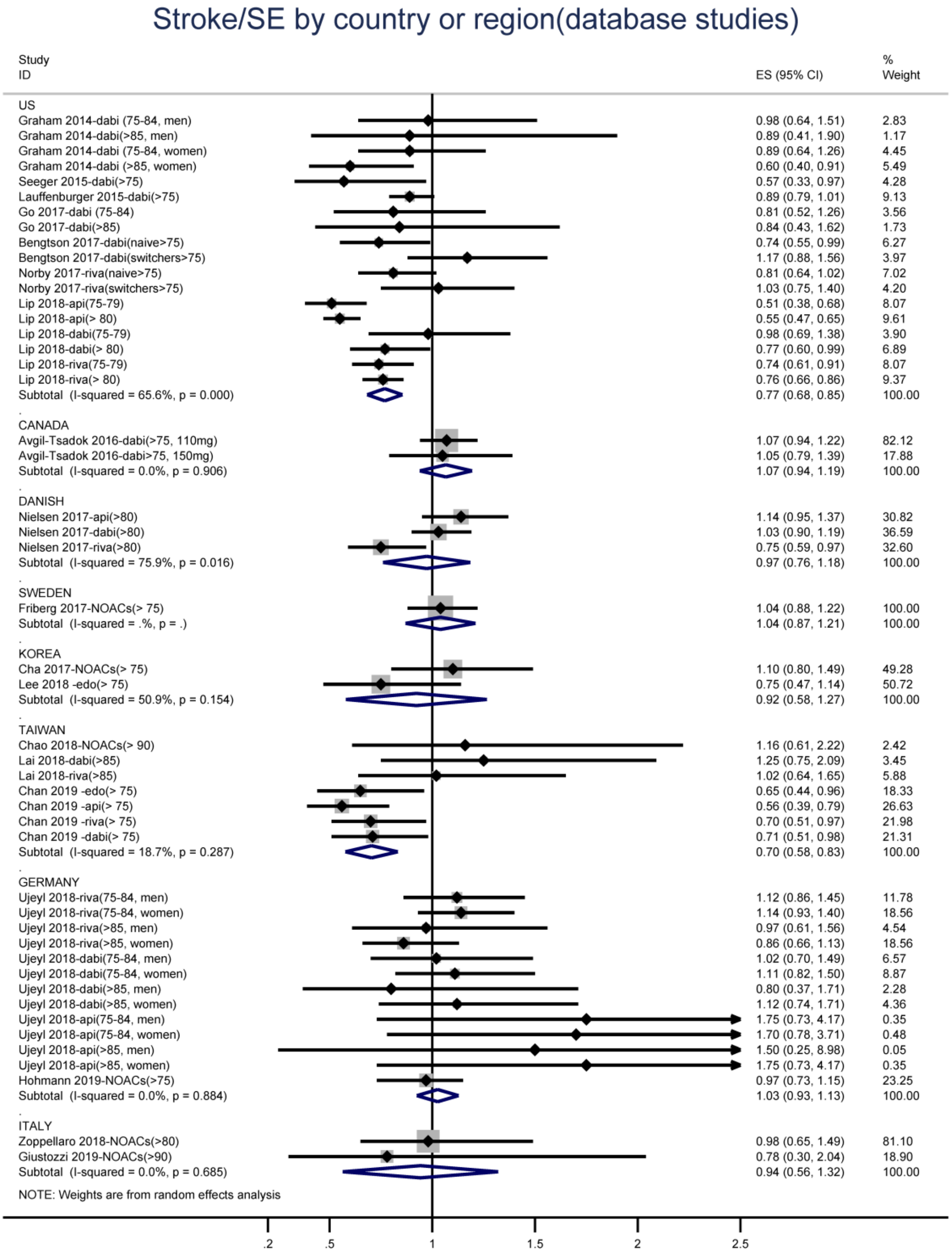

Supplementary Figure 19. Stroke/SE by country or region of OSs

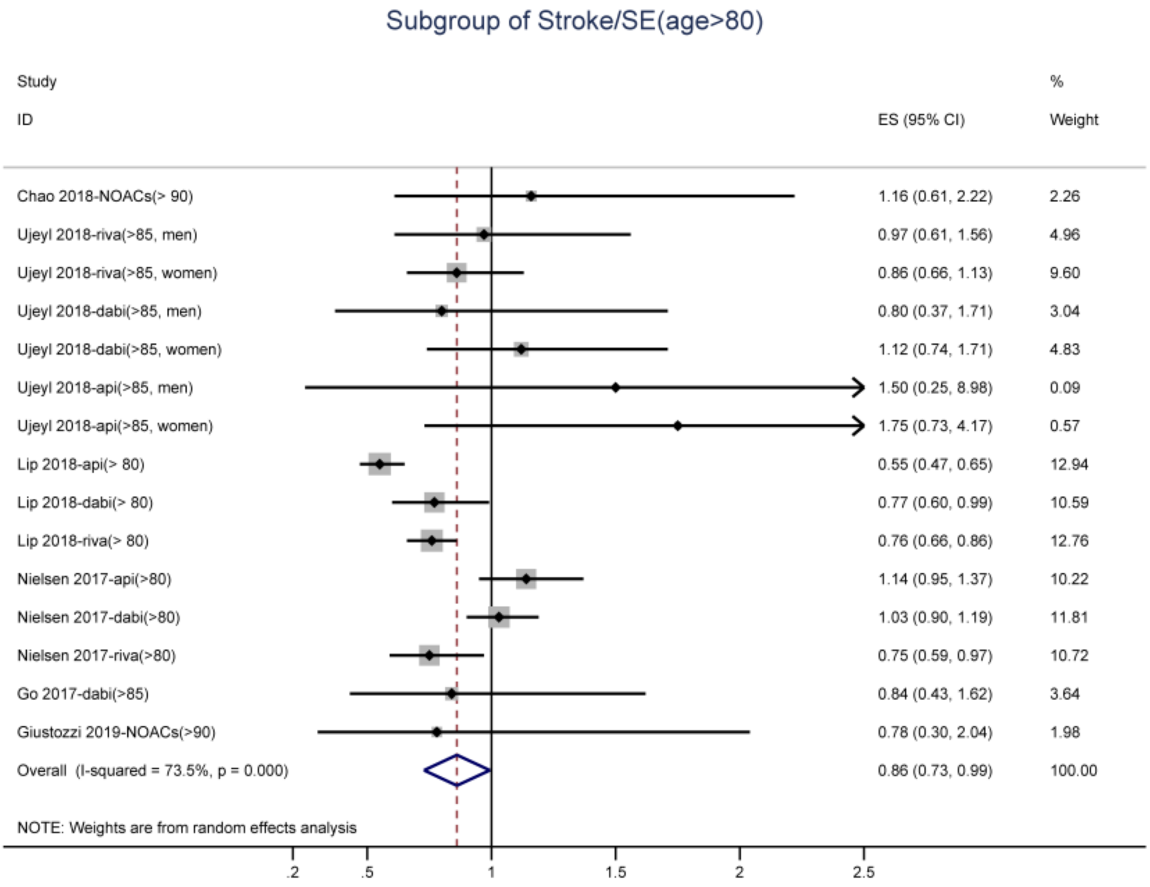

Supplementary Figure 20. Stroke/SE of age>80 (OSs)

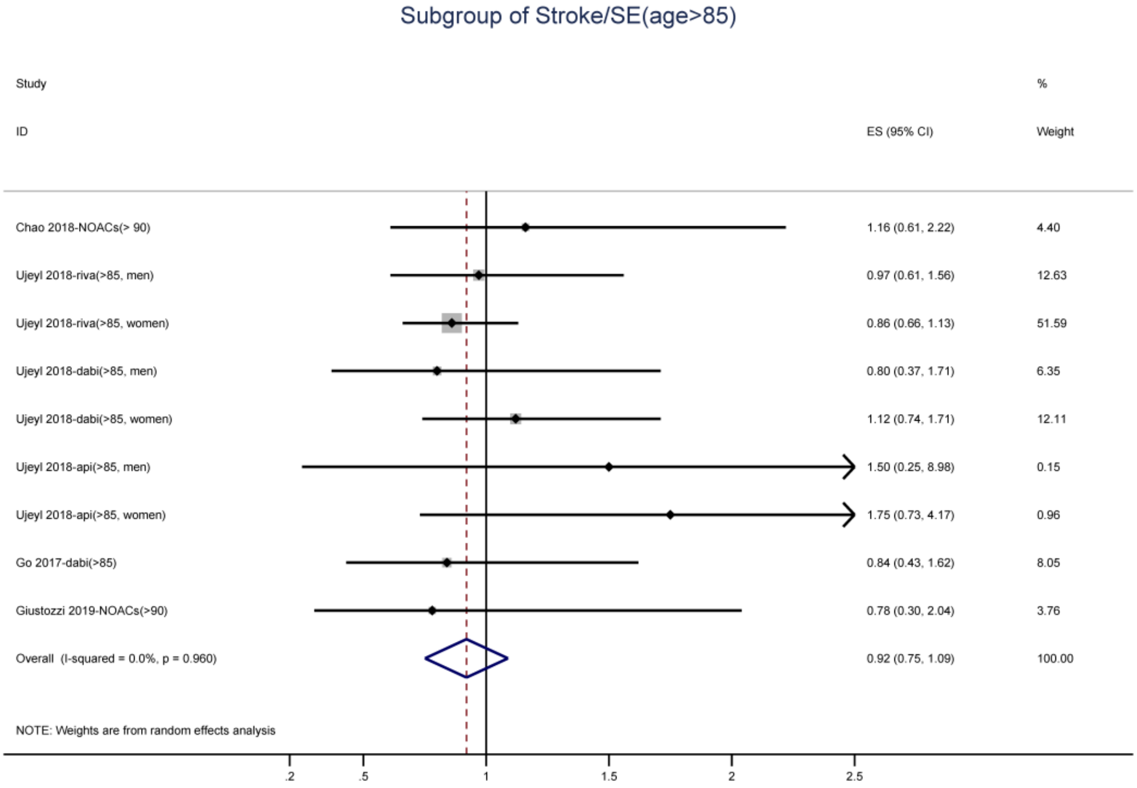

Supplementary Figure 21. Stroke/SE of age>85 (OSs)

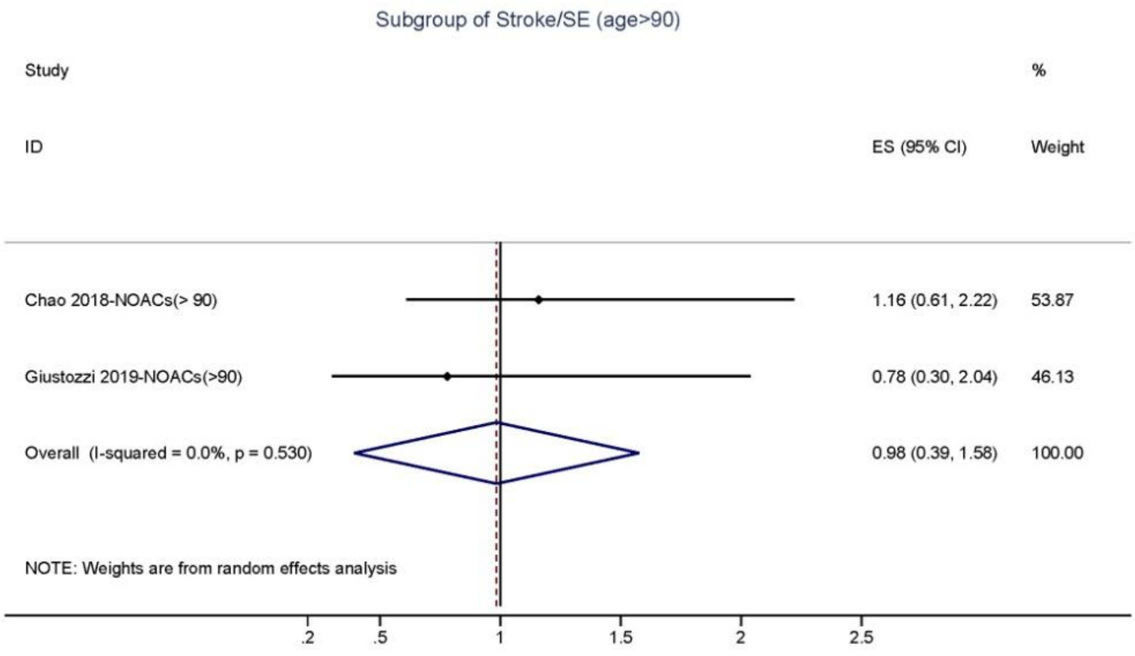

Supplementary Figure 22. Stroke/SE of age>90 (OSs)

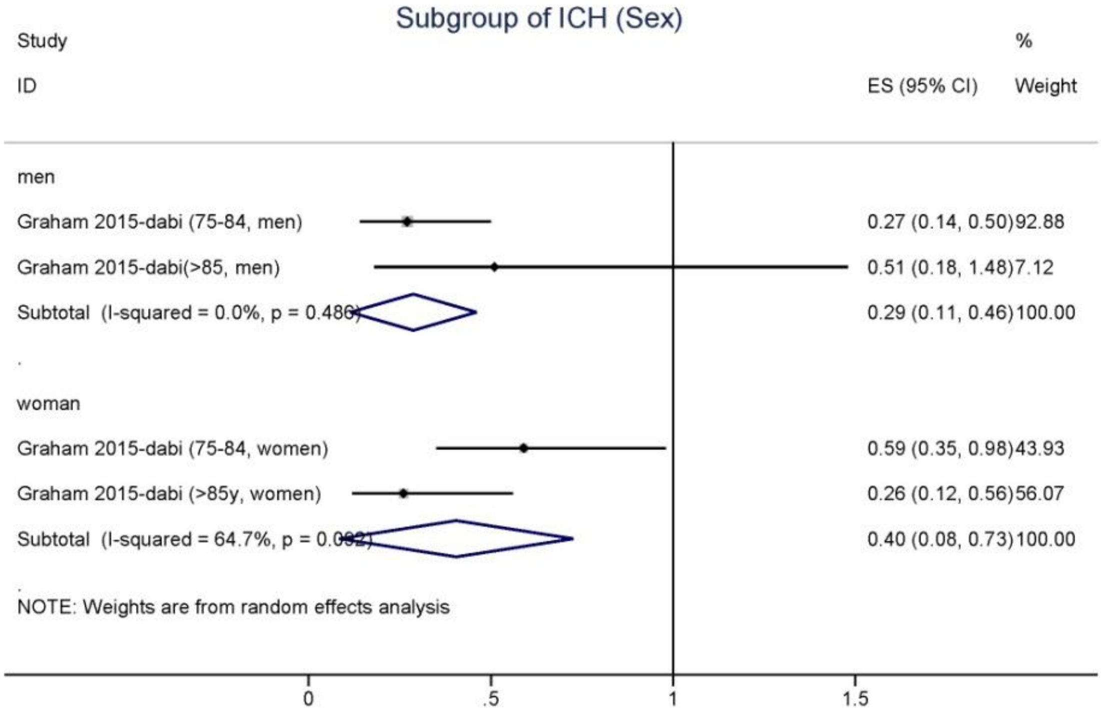

Supplementary Figure 23. ICH by gender of OSs

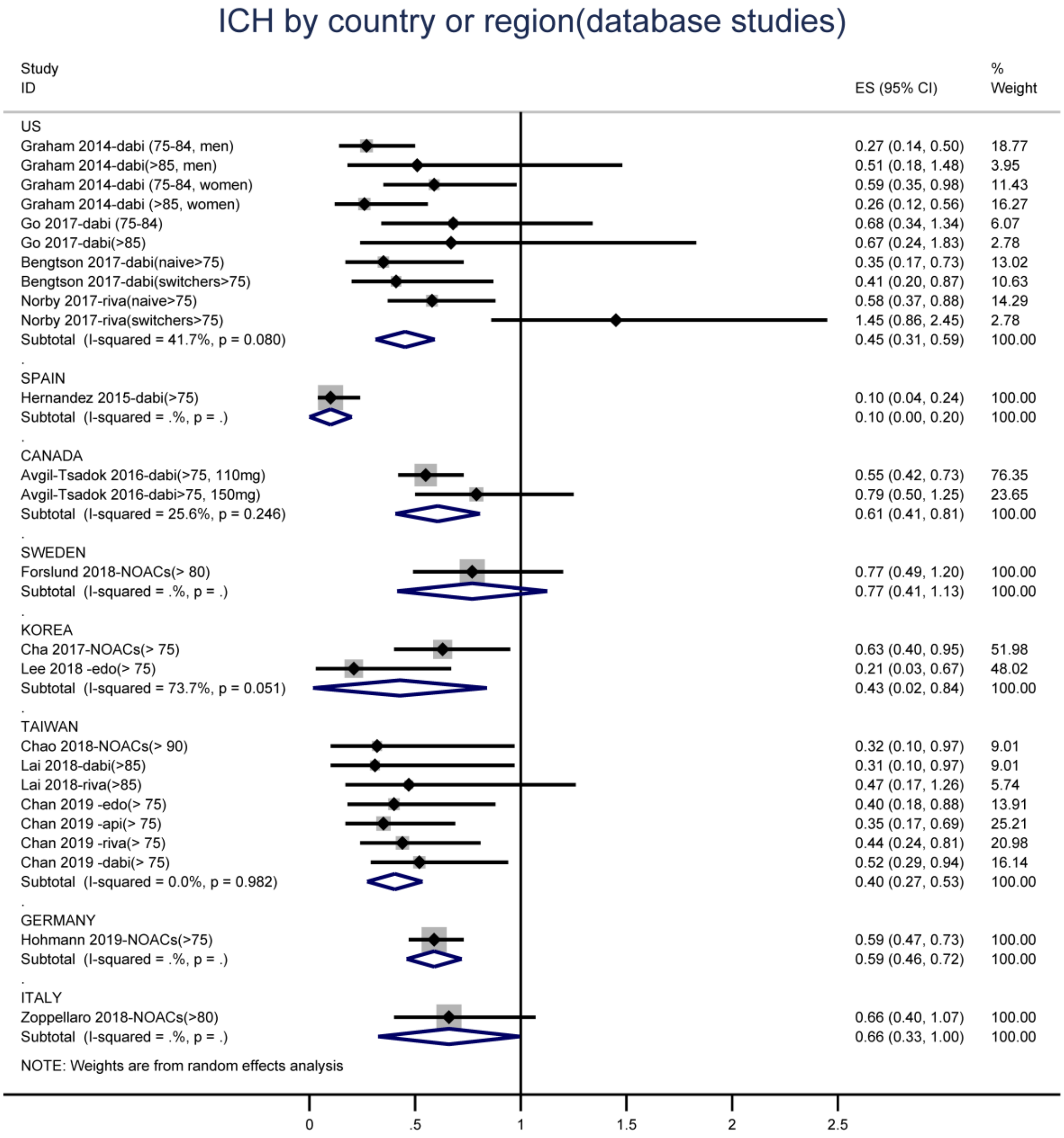

Supplementary Figure 24. ICH by country or region of OSs

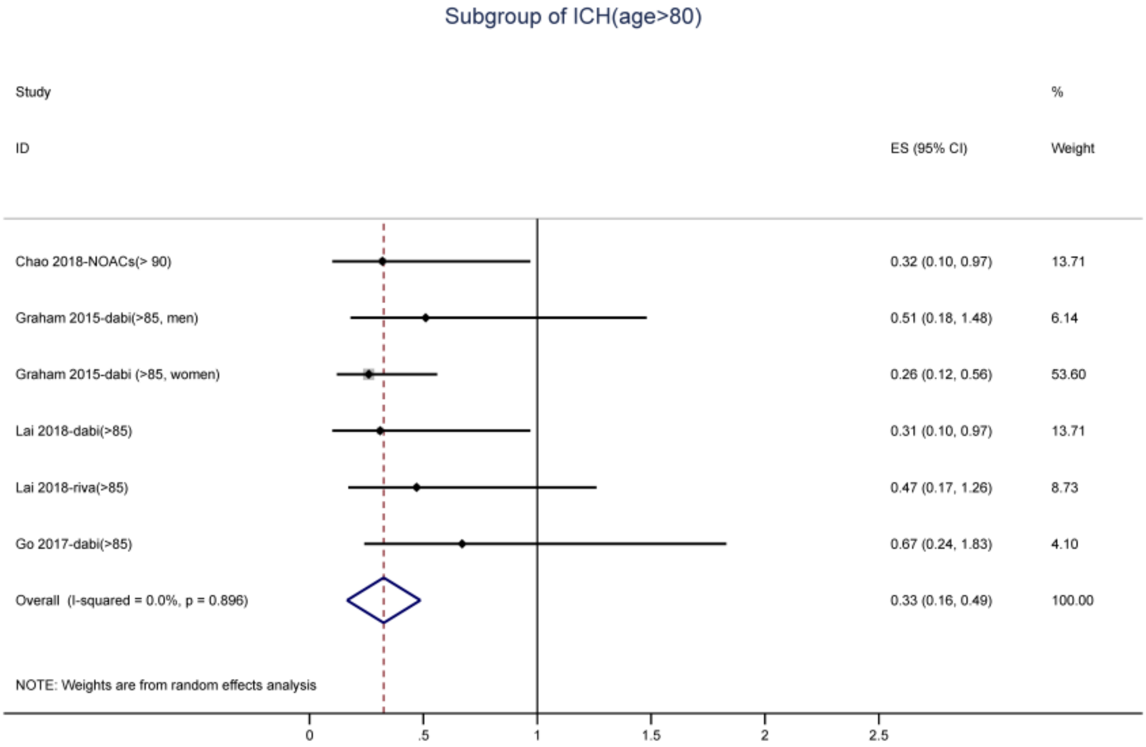

Supplementary Figure 25. ICH of age>80 (OSs)

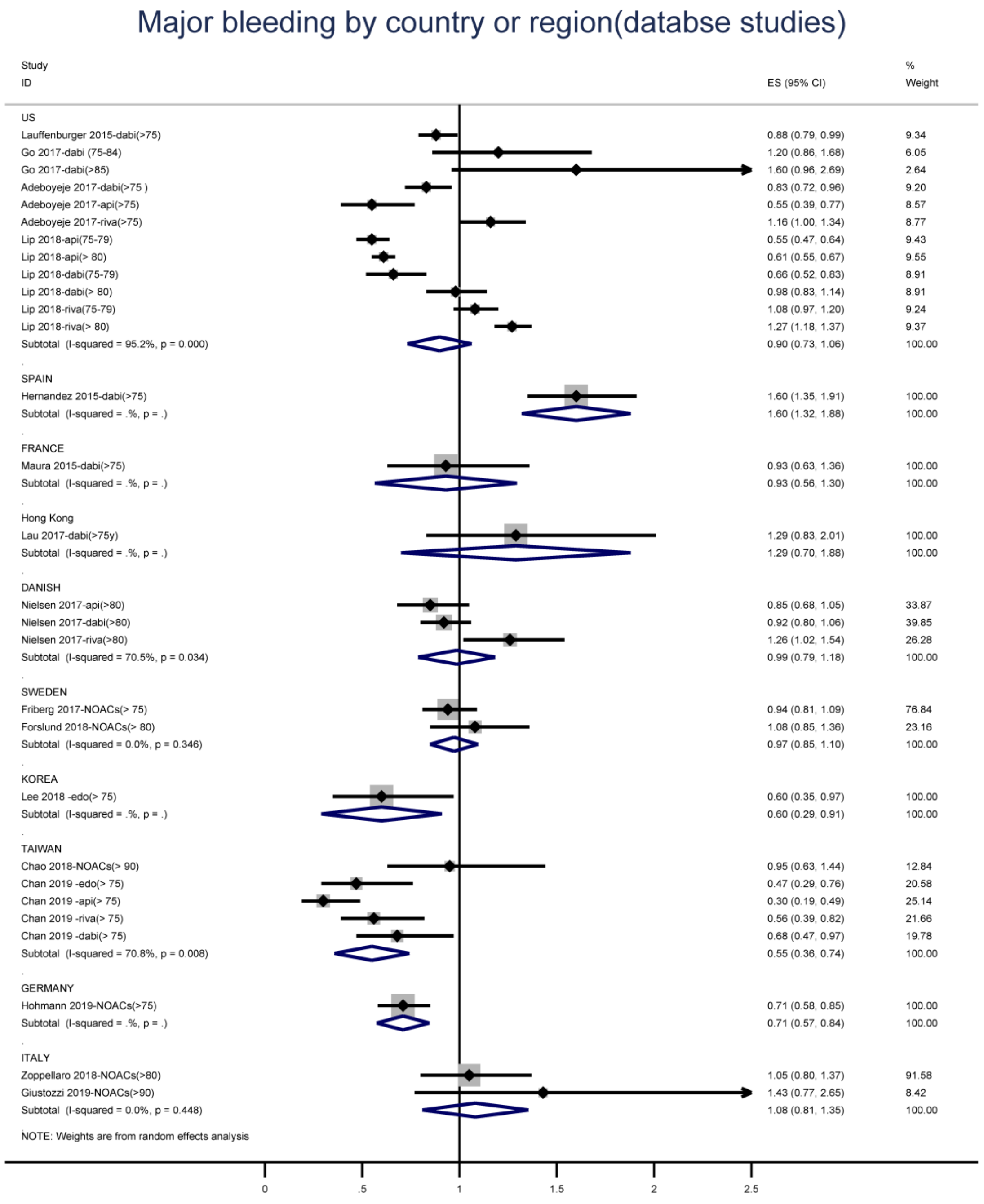

Supplementary Figure 26. Major bleeding by country or region of OSs

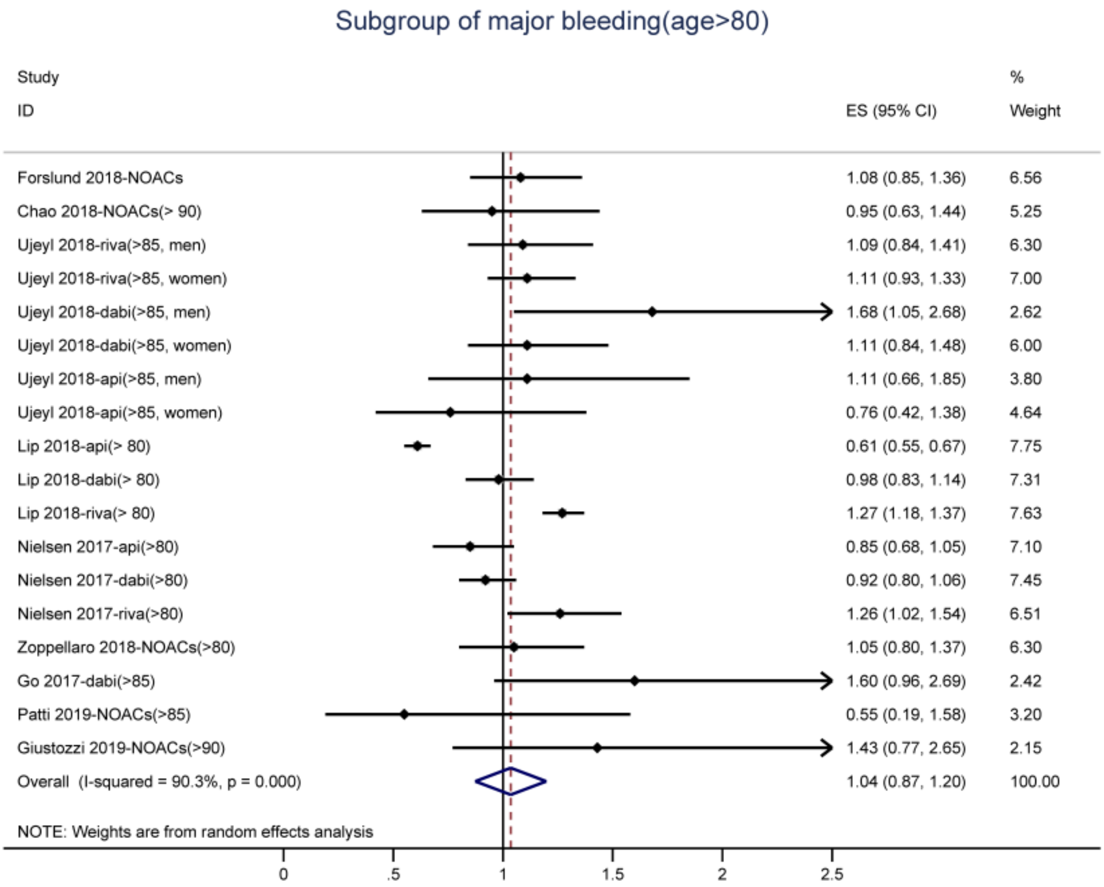

Supplementary Figure 27. Major bleeding of age>80 (OSs)

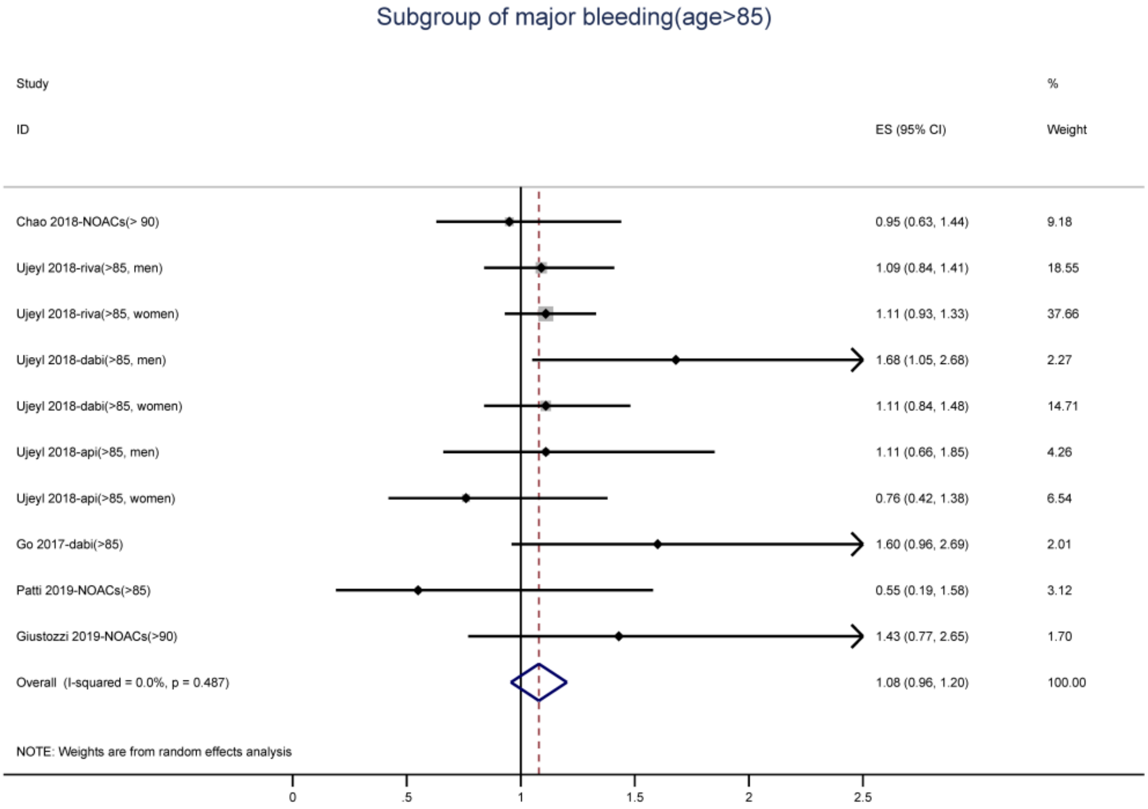

Supplementary Figure 28. Major bleeding of age>85 (OSs)

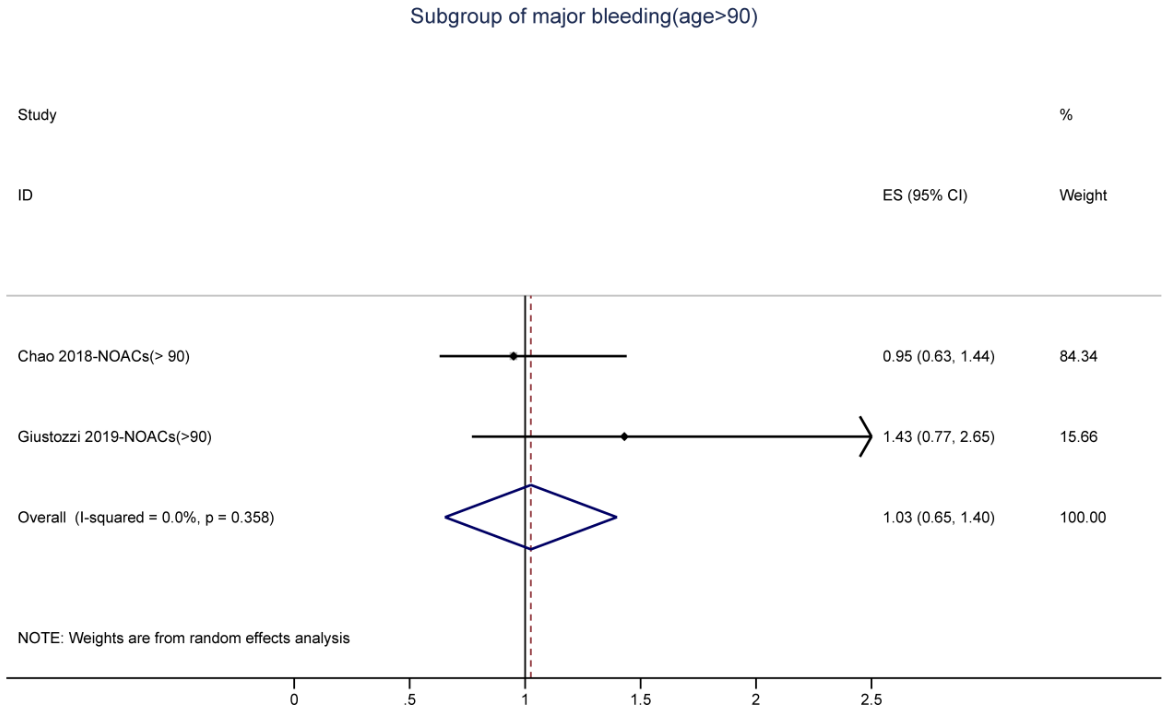

Supplementary Figure 29. Major bleeding of age>90 (OSs)

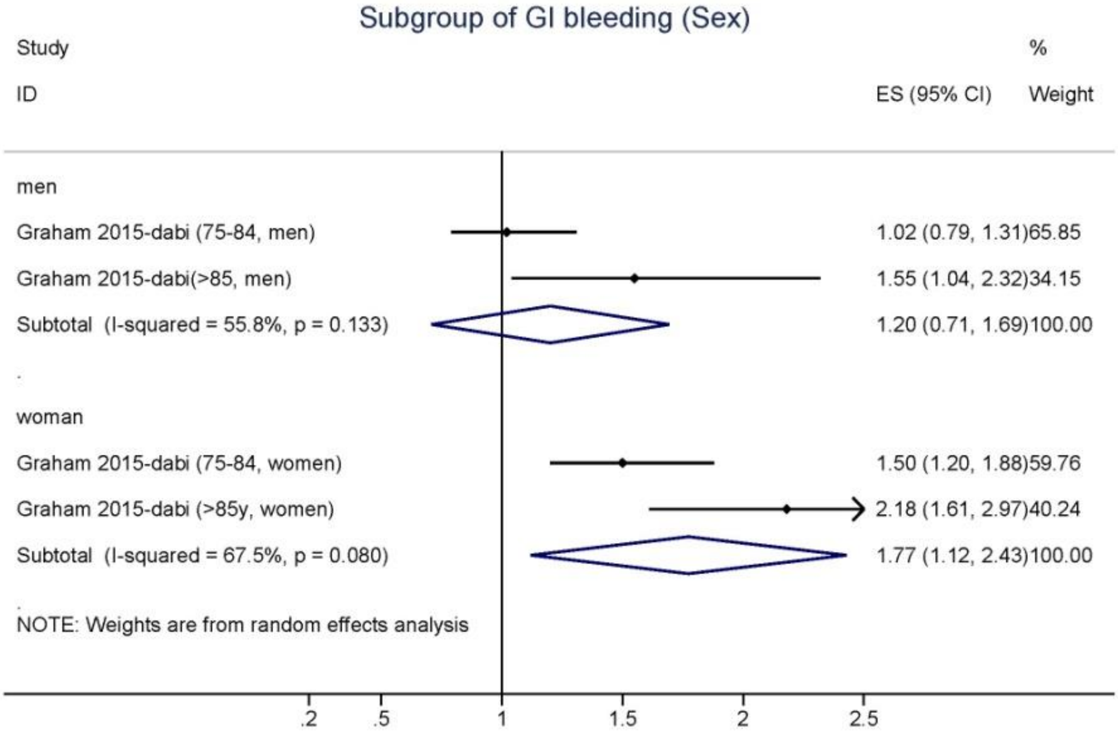

Supplementary Figure 30. GI bleeding by gender of OSs

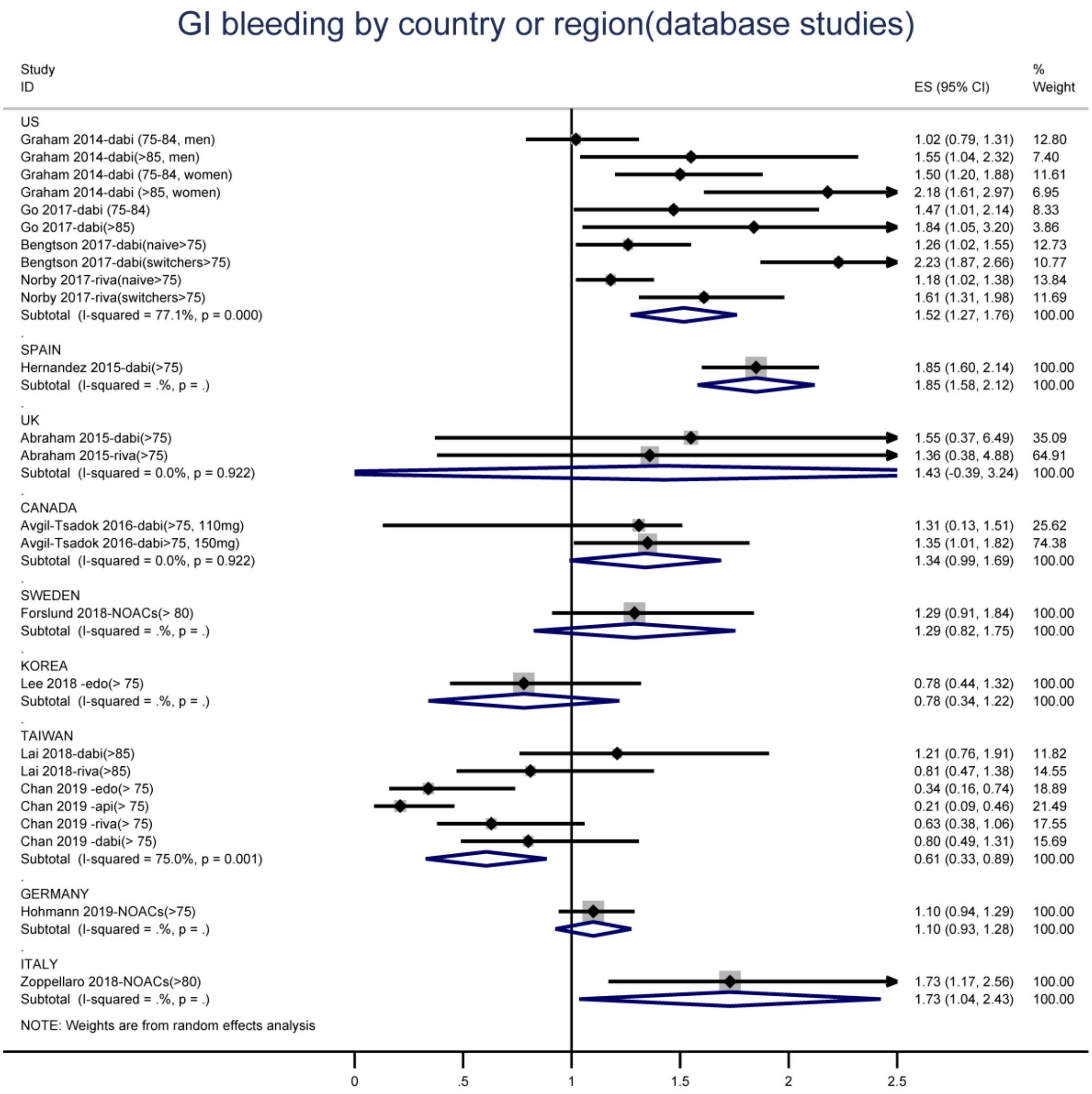

Supplementary Figure 31. GI bleeding by country or region of OSs

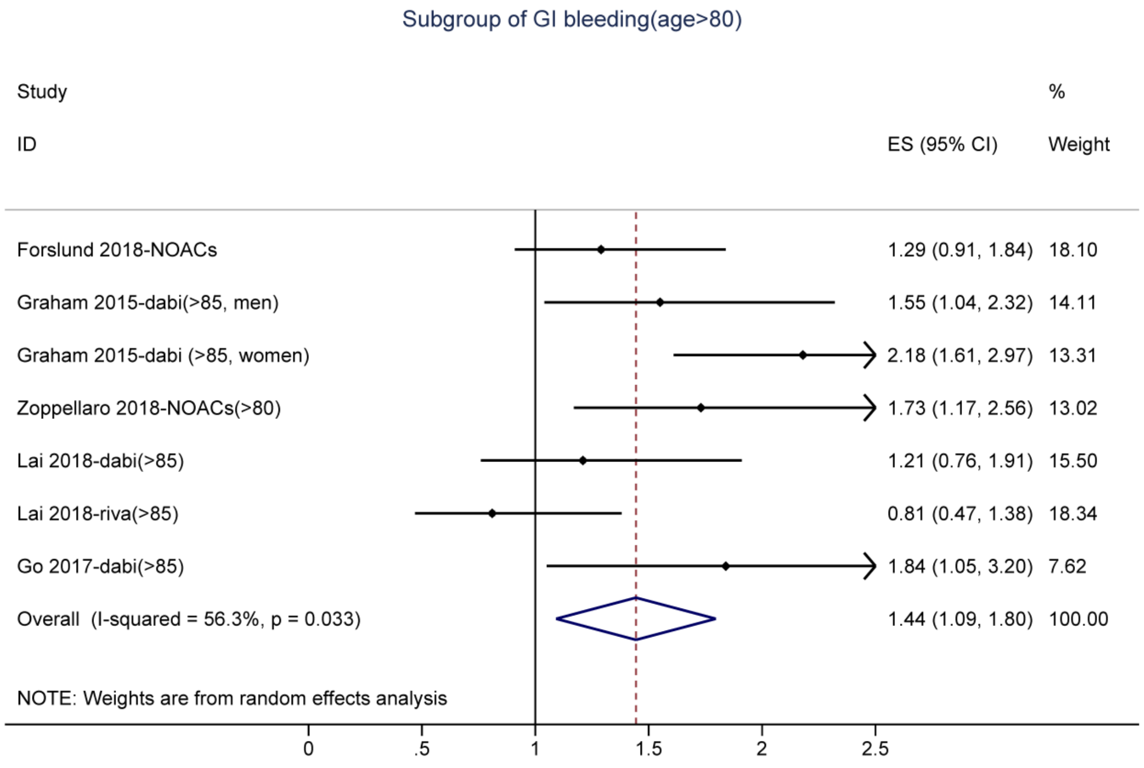

Supplementary Figure 32. GI bleeding of age>80 (OSs)

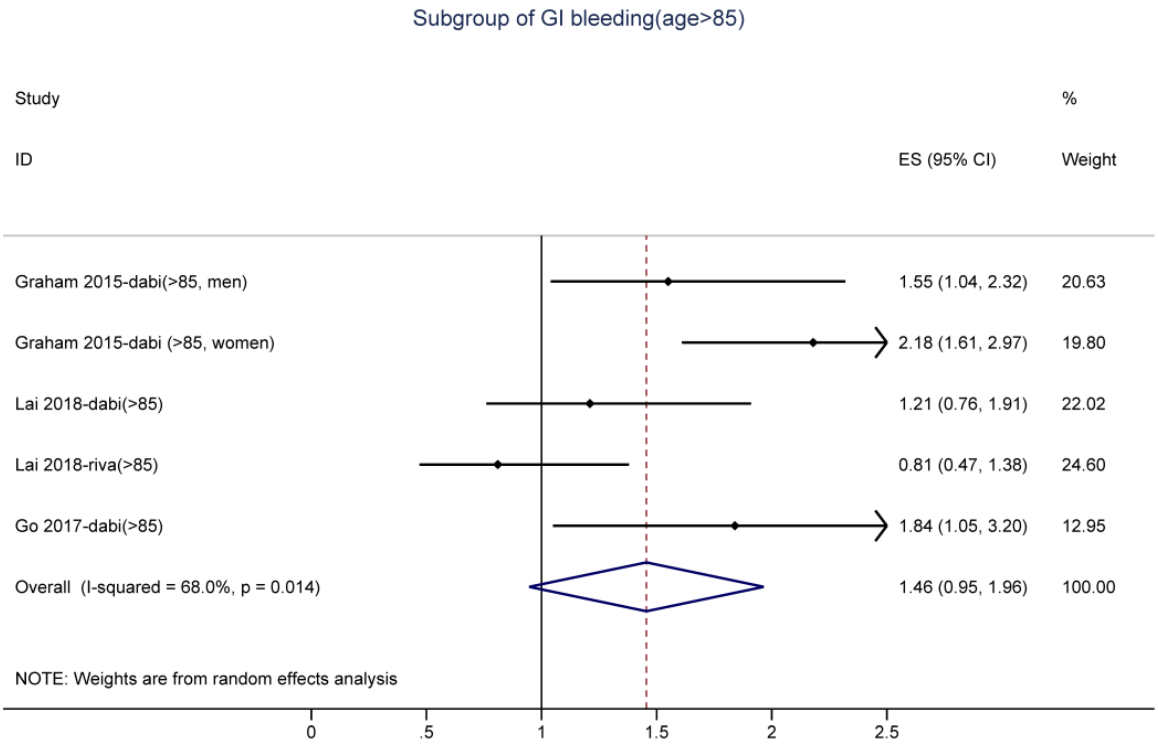

Supplementary Figure 33. GI bleeding of age>85 (OSs)

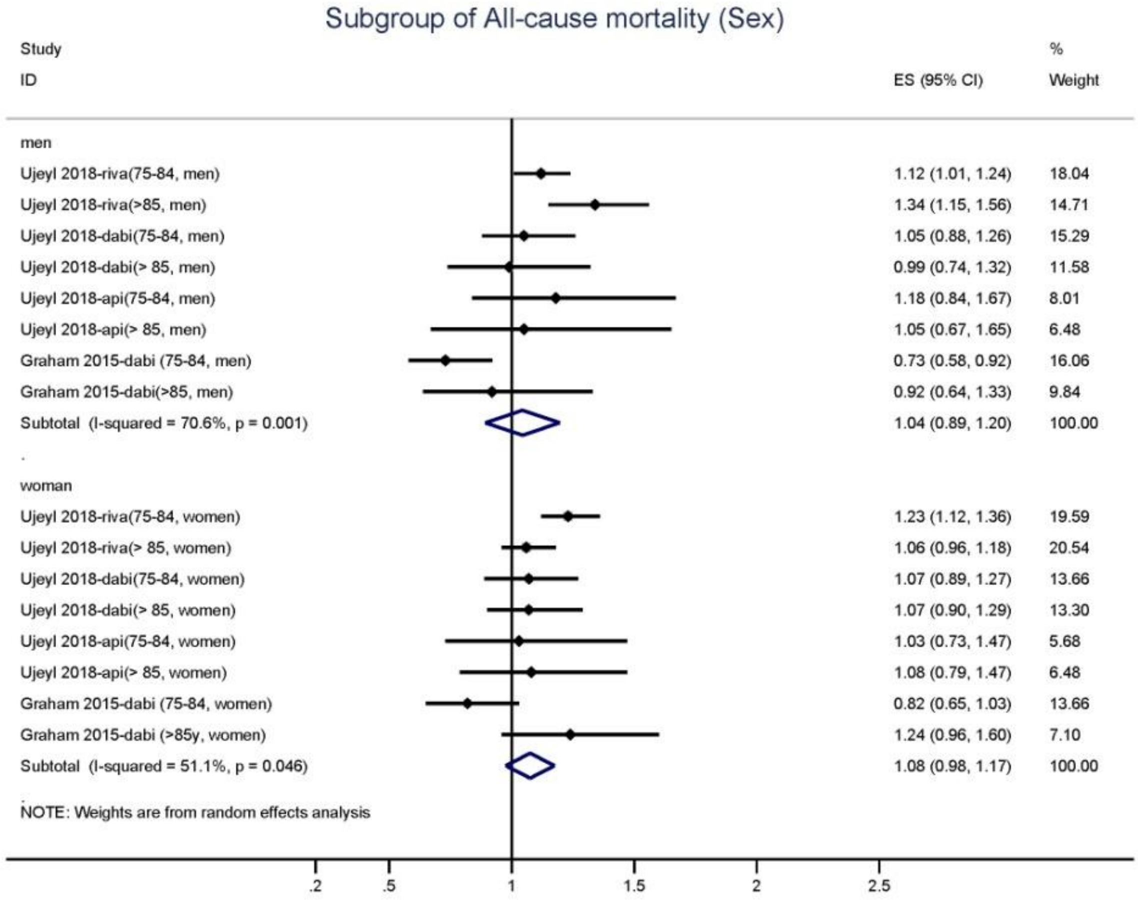

Supplementary Figure 34. All-cause mortality by gender of OSs

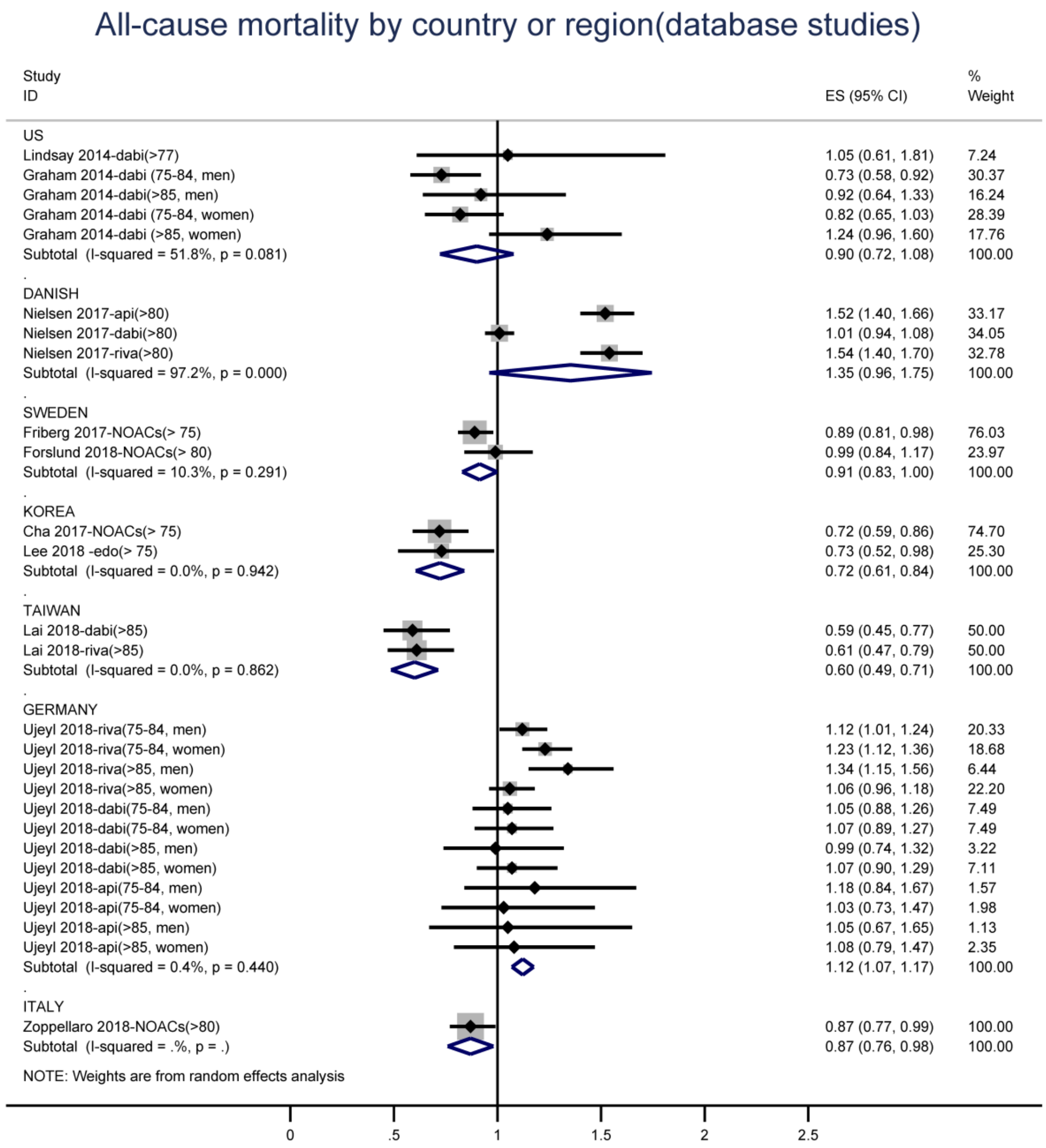

Supplementary Figure 35. All-cause mortality by country or region of OSs

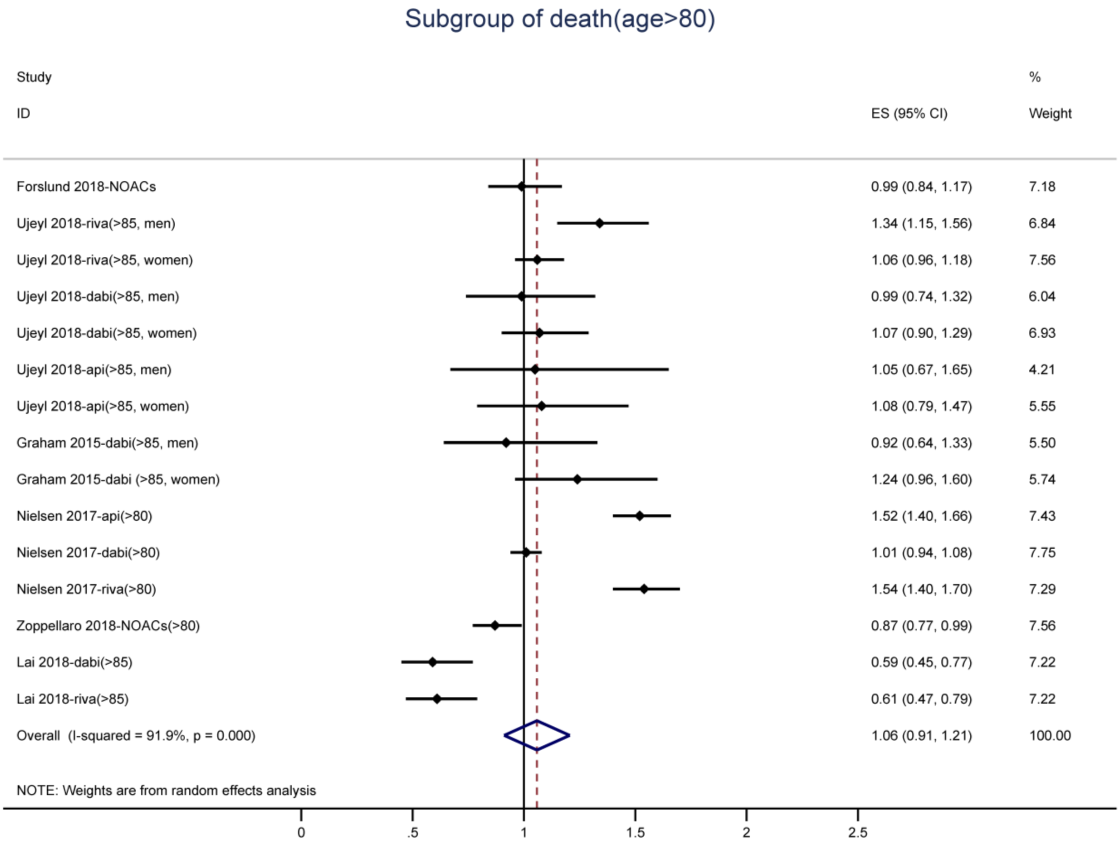

Supplementary Figure 36. All-cause mortality of age>80 (OSs)

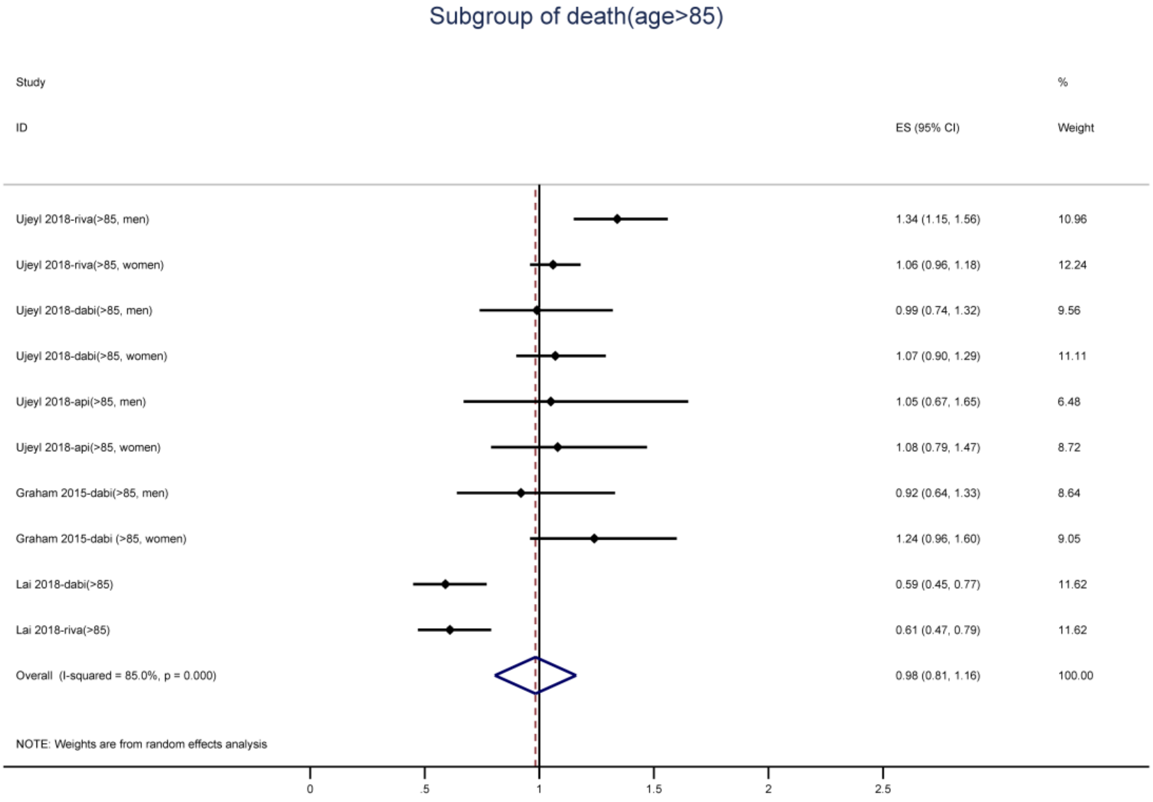

Supplementary Figure 37. All-cause mortality of age>85 (OSs)

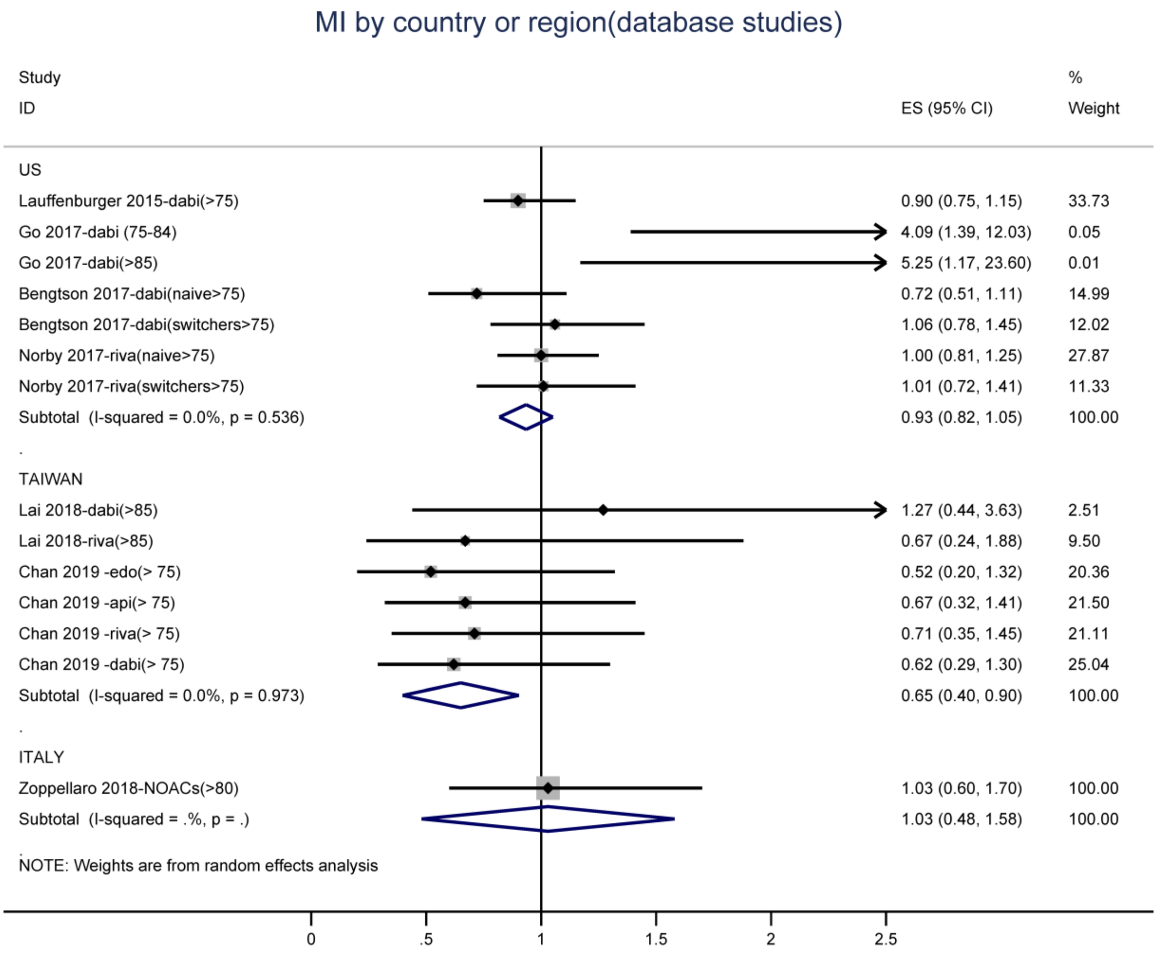

Supplementary Figure 38. MI by country or region of OSs

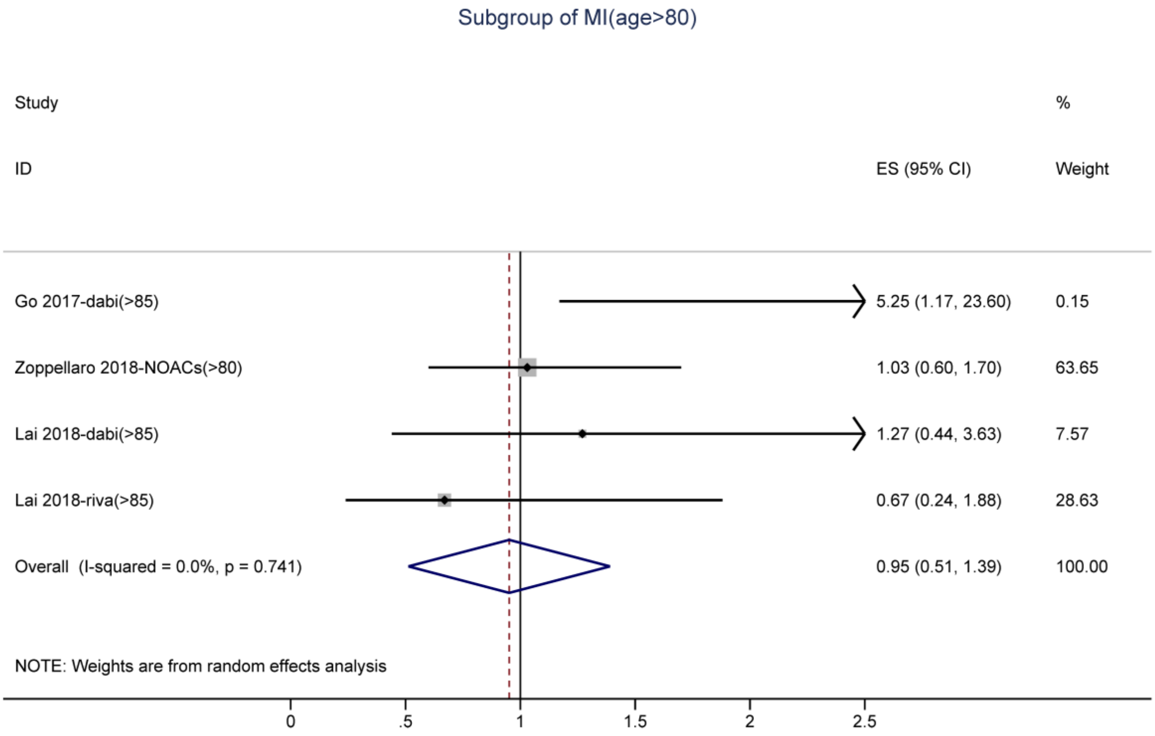

Supplementary Figure 39. MI of age>80 (OSs)

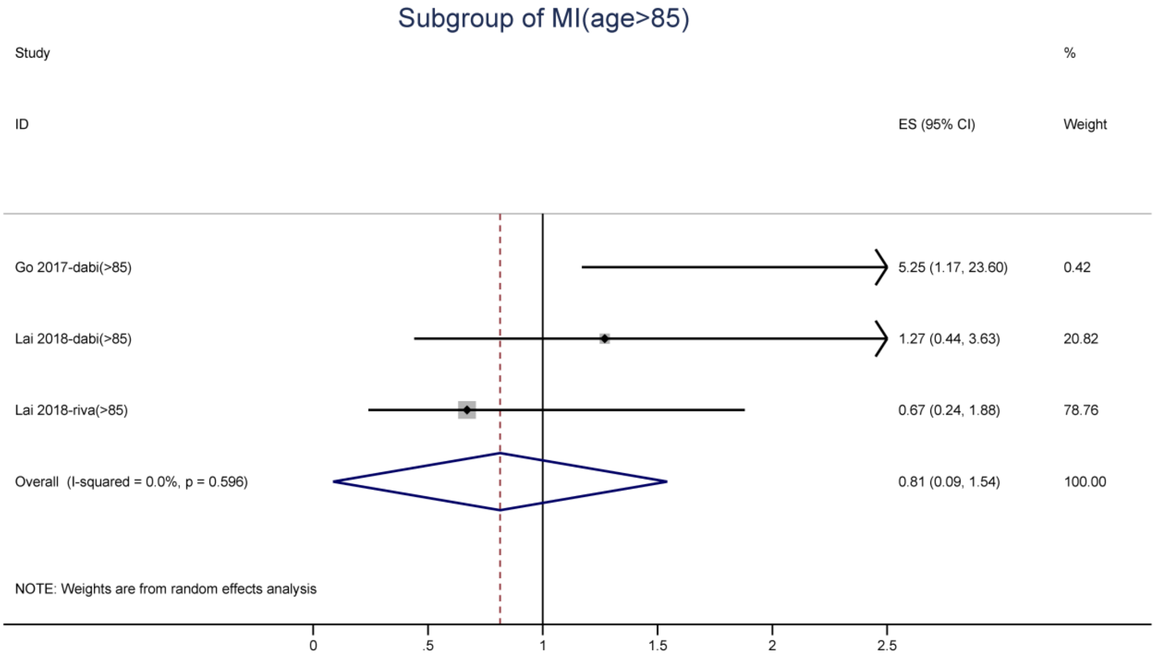

Supplementary Figure 40. MI of age>85 (OSs)

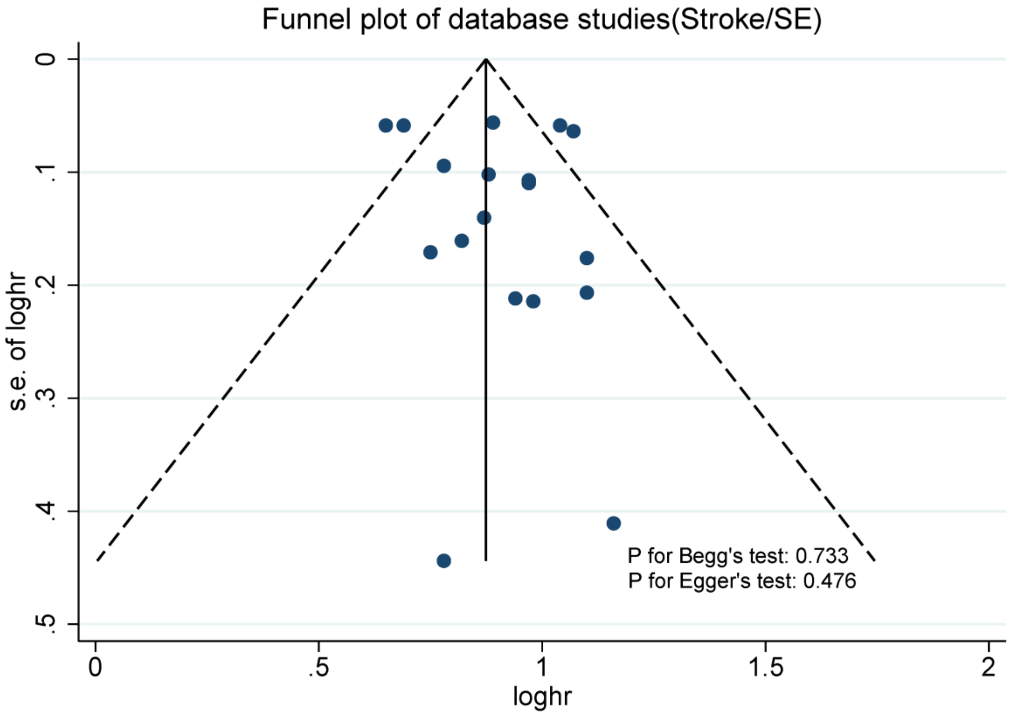

Supplementary Figure 41. Funnel Plot of OSs (Stroke/SE)

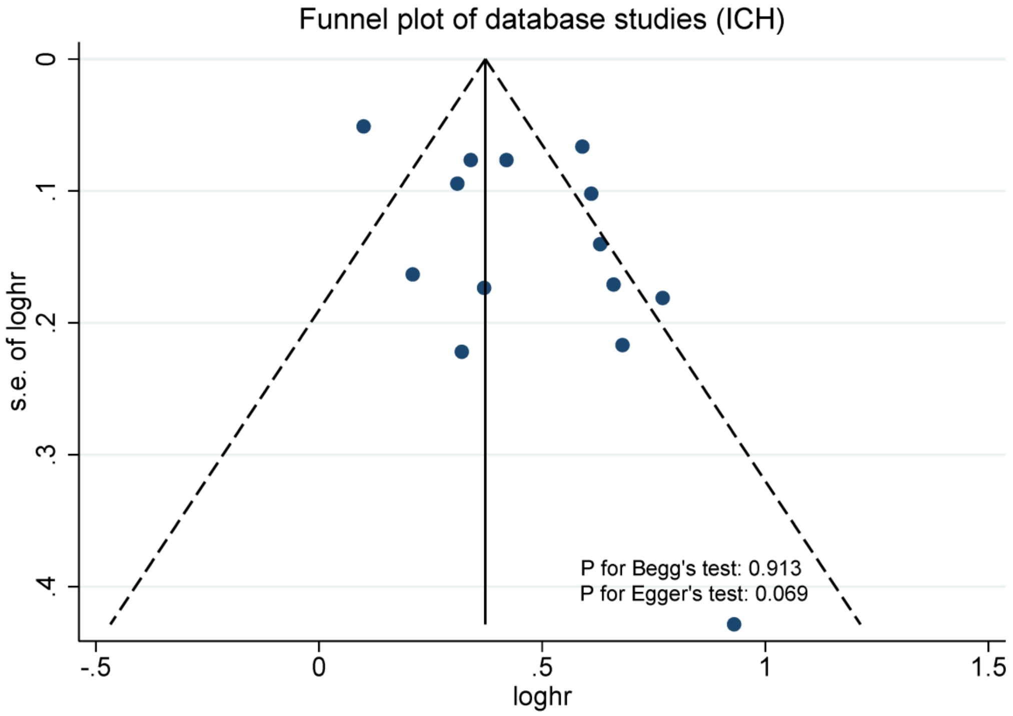

Supplementary Figure 42. Funnel Plot of OSs (ICH)

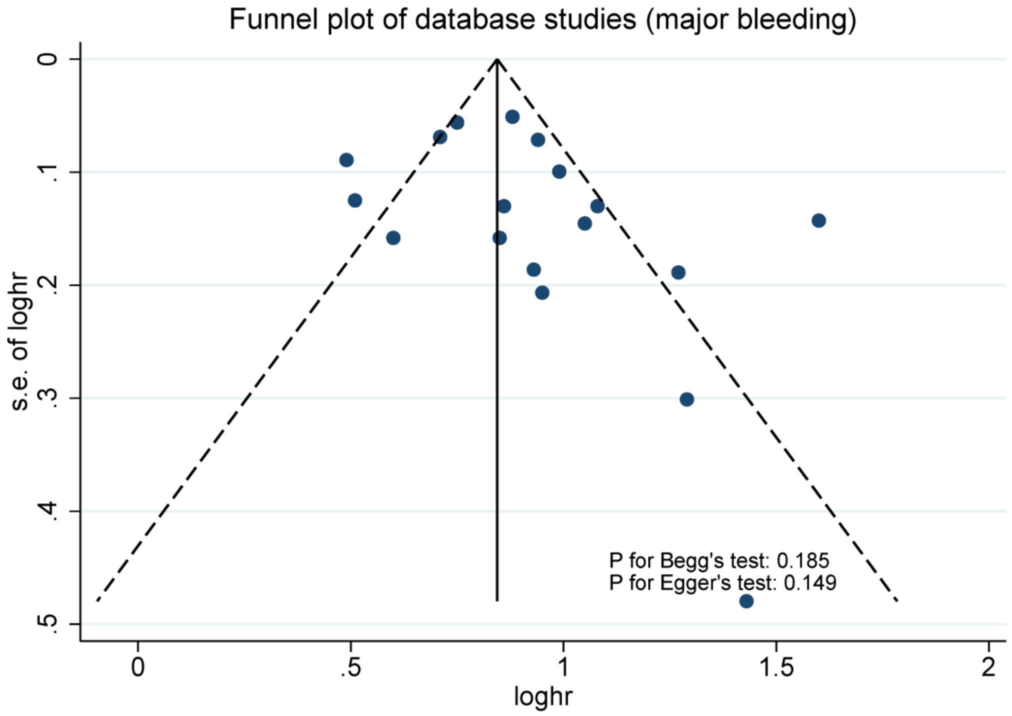

Supplementary Figure 43. Funnel Plot of OSs (major bleeding)

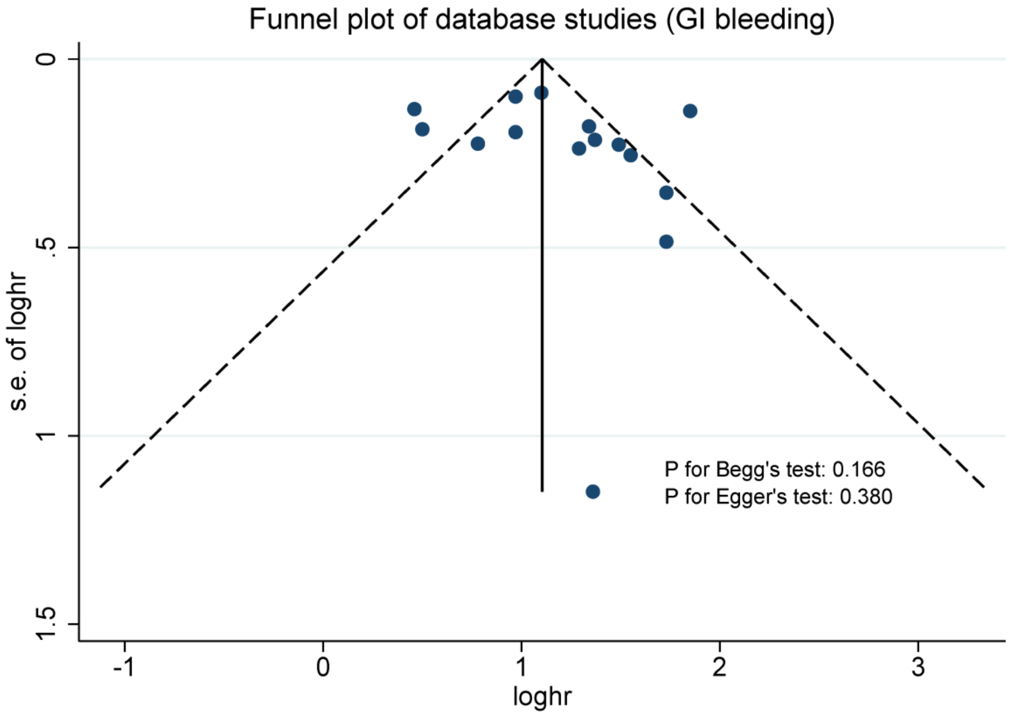

Supplementary Figure 44. Funnel Plot of OSs (GI bleeding)

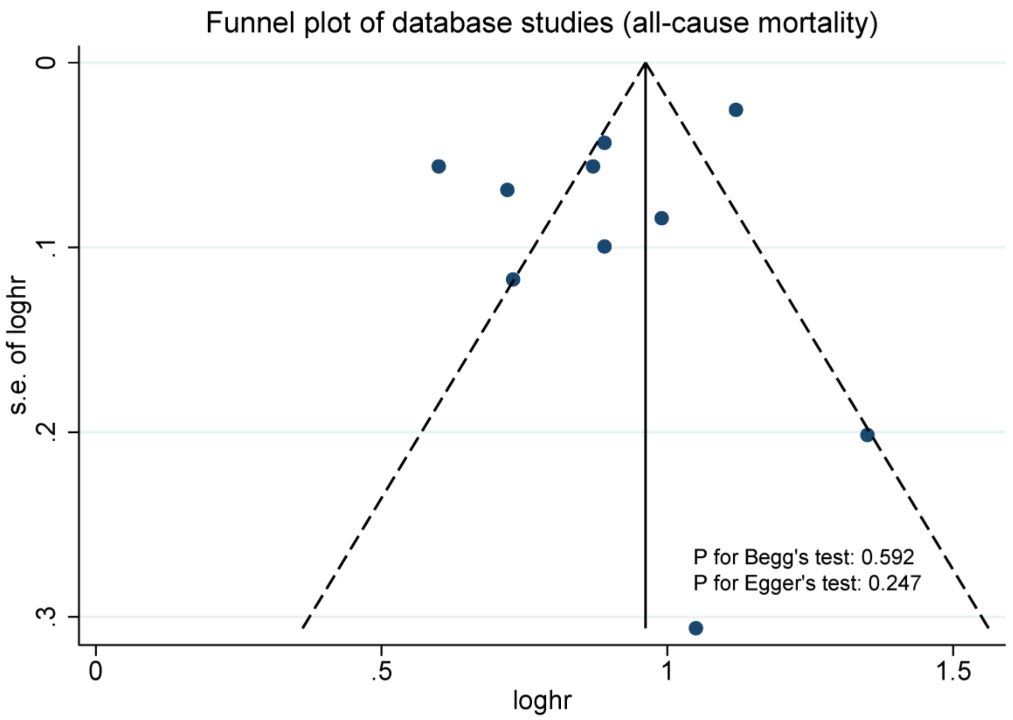

Supplementary Figure 45. Funnel Plot of OSs (all-cause mortality)

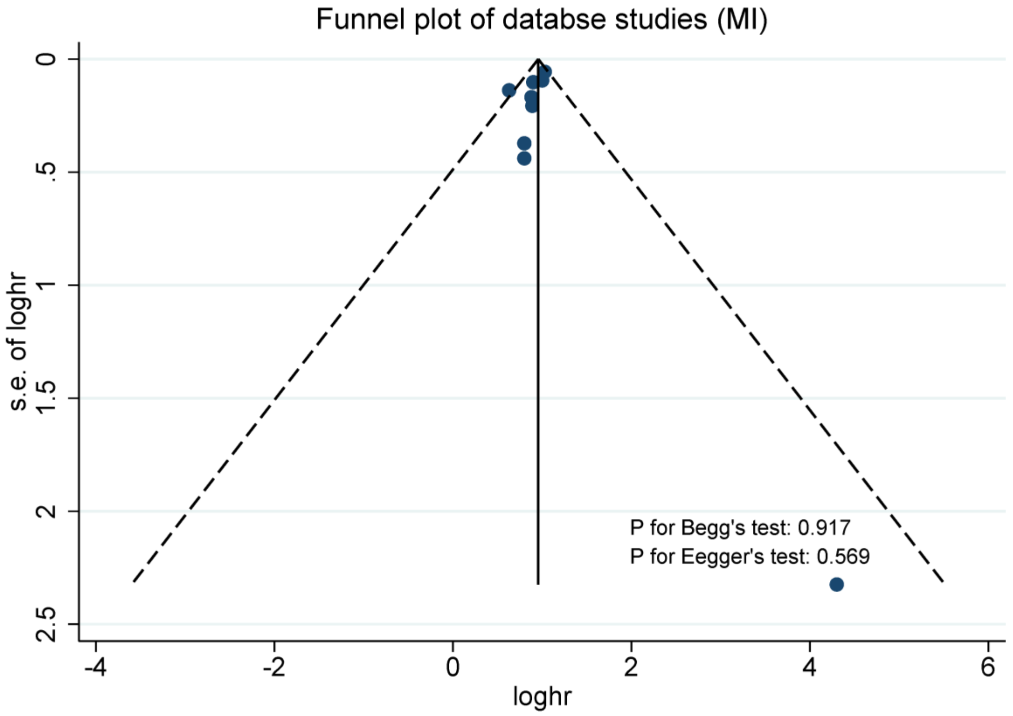

Supplementary Figure 46. Funnel Plot of OSs (MI)

References

Abe, J., Umetsu, R., Kato, Y., Ueda, N., Nakayama, Y., Suzuki, Y., et al. (2015). Evaluation of Dabigatran- and Warfarin-Associated Hemorrhagic Events Using the FDA-Adverse Event Reporting System Database Stratified by Age. *Int J Med Sci* 12(4)**,** 312-321. doi: 10.7150/ijms.10703.

Abraham, N.S., Hartman, C., Richardson, P., Castillo, D., Street, R.L., Jr., and Naik, A.D. (2013). Risk of lower and upper gastrointestinal bleeding, transfusions, and hospitalizations with complex antithrombotic therapy in elderly patients. *Circulation* 128(17)**,** 1869-1877. doi: 10.1161/CIRCULATIONAHA.113.004747.

Abraham, N.S., Singh, S., Alexander, G.C., Heien, H., Haas, L.R., Crown, W., et al. (2015). Comparative risk of gastrointestinal bleeding with dabigatran, rivaroxaban, and warfarin: population based cohort study. *BMJ* 350**,** h1857. doi: 10.1136/bmj.h1857.

Adeboyeje, G., Sylwestrzak, G., Barron, J.J., White, J., Rosenberg, A., Abarca, J., et al. (2017). Major Bleeding Risk During Anticoagulation with Warfarin, Dabigatran, Apixaban, or Rivaroxaban in Patients with Nonvalvular Atrial Fibrillation. *J Manag Care Spec Pharm* 23(9)**,** 968-978. doi: 10.18553/jmcp.2017.23.9.968.

Amin, A., Keshishian, A., Vo, L., Zhang, Q., Dina, O., Patel, C., et al. (2018). Real-world comparison of all-cause hospitalizations, hospitalizations due to stroke and major bleeding, and costs for non-valvular atrial fibrillation patients prescribed oral anticoagulants in a US health plan. *J Med Econ* 21(3)**,** 244-253. doi: 10.1080/13696998.2017.1394866.

Andersson, N.W., Svanstrom, H., Lund, M., Pasternak, B., and Melbye, M. (2018). Comparative effectiveness and safety of apixaban, dabigatran, and rivaroxaban in patients with non-valvular atrial fibrillation. *Int J Cardiol*. doi: 10.1136/bmjopen-2017-020286

10.1016/j.ijcard.2018.03.047.

Arihiro, S., Todo, K., Koga, M., Furui, E., Kinoshita, N., Kimura, K., et al. (2016). Three-month risk-benefit profile of anticoagulation after stroke with atrial fibrillation: The SAMURAI-Nonvalvular Atrial Fibrillation (NVAF) study. *Int J Stroke* 11(5)**,** 565-574. doi: 10.1177/1747493016632239.

Avgil-Tsadok, M., Jackevicius, C.A., Essebag, V., Eisenberg, M.J., Rahme, E., Behlouli, H., et al. (2016). Dabigatran use in elderly patients with atrial fibrillation. *Thromb Haemost* 115(1)**,** 152-160. doi: 10.1160/TH15-03-0247.

Becattini, C., Franco, L., Beyer-Westendorf, J., Masotti, L., Nitti, C., Vanni, S., et al. (2017). Major bleeding with vitamin K antagonists or direct oral anticoagulants in real-life. *Int J Cardiol* 227**,** 261-266. doi: 10.1016/j.ijcard.2016.11.117.

Bengtson, L.G.S., Lutsey, P.L., Chen, L.Y., MacLehose, R.F., and Alonso, A. (2017). Comparative effectiveness of dabigatran and rivaroxaban versus warfarin for the treatment of non-valvular atrial fibrillation. *J Cardiol* 69(6)**,** 868-876. doi: 10.1016/j.jjcc.2016.08.010.

Cappato, R., Marchlinski, F.E., Hohnloser, S.H., Naccarelli, G.V., Xiang, J., Wilber, D.J., et al. (2015). Uninterrupted rivaroxaban vs. uninterrupted vitamin K antagonists for catheter ablation in non-valvular atrial fibrillation. *Eur Heart J* 36(28)**,** 1805-1811. doi: 10.1093/eurheartj/ehv177.

Cha, M.J., Choi, E.K., Han, K.D., Lee, S.R., Lim, W.H., Oh, S., et al. (2017). Effectiveness and Safety of Non-Vitamin K Antagonist Oral Anticoagulants in Asian Patients With Atrial Fibrillation. *Stroke* 48(11)**,** 3040-3048. doi: 10.1161/strokeaha.117.018773.

Chan, K.E., Edelman, E.R., Wenger, J.B., Thadhani, R.I., and Maddux, F.W. (2015). Dabigatran and rivaroxaban use in atrial fibrillation patients on hemodialysis. *Circulation* 131(11)**,** 972-979. doi: 10.1161/circulationaha.114.014113.

Chan, Y.-H., Lee, H.-F., See, L.-C., Tu, H.-T., Chao, T.-F., Yeh, Y.-H., et al. (2019). Effectiveness and Safety of Four Direct Oral Anticoagulants in Asian Patients With Nonvalvular Atrial Fibrillation. *Chest*. doi: 10.1016/j.chest.2019.04.108.

Chan, Y.H., Yen, K.C., See, L.C., Chang, S.H., Wu, L.S., Lee, H.F., et al. (2016). Cardiovascular, Bleeding, and Mortality Risks of Dabigatran in Asians With Nonvalvular Atrial Fibrillation. *Stroke* 47(2)**,** 441-449. doi: 10.1161/STROKEAHA.115.011476.

Chao, T.F., Liu, C.J., Lin, Y.J., Chang, S.L., Lo, L.W., Hu, Y.F., et al. (2018). Oral Anticoagulation in Very Elderly Patients With Atrial Fibrillation: A Nationwide Cohort Study. *Circulation* 138(1)**,** 37-47. doi: 10.1161/circulationaha.117.031658.

Coleman, C.I., Antz, M., Bowrin, K., Evers, T., Simard, E.P., Bonnemeier, H., et al. (2016). Real-world evidence of stroke prevention in patients with nonvalvular atrial fibrillation in the United States: the REVISIT-US study. *Curr Med Res Opin* 32(12)**,** 2047-2053. doi: 10.1080/03007995.2016.1237937.

Coleman, C.I., Peacock, W.F., and Antz, M. (2018). Comparative Effectiveness and Safety of Apixaban and Vitamin K Antagonist Therapy in Patients With Nonvalvular Atrial Fibrillation Treated in Routine German Practice. *Heart Lung Circ* 27(3)**,** 390-393. doi: 10.1016/j.hlc.2017.04.002.

Connolly, S.J., Eikelboom, J., Dorian, P., Hohnloser, S.H., Gretler, D.D., Sinha, U., et al. (2013). Betrixaban compared with warfarin in patients with atrial fibrillation: results of a phase 2, randomized, dose-ranging study (Explore-Xa). *Eur Heart J* 34(20)**,** 1498-1505. doi: 10.1093/eurheartj/eht039.

Connolly, S.J., Ezekowitz, M.D., Yusuf, S., Eikelboom, J., Oldgren, J., Parekh, A., et al. (2009). Dabigatran versus warfarin in patients with atrial fibrillation. *N Engl J Med* 361(12)**,** 1139-1151. doi: 10.1056/NEJMoa0905561.

Deitelzweig, S., Luo, X., Gupta, K., Trocio, J., Mardekian, J., Curtice, T., et al. (2017). Comparison of effectiveness and safety of treatment with apixaban vs. other oral anticoagulants among elderly nonvalvular atrial fibrillation patients. *Curr Med Res Opin* 33(10)**,** 1745-1754. doi: 10.1080/03007995.2017.1334638.

Ellis, M.H., Neuman, T., Bitterman, H., Dotan, S.G., Hammerman, A., Battat, E., et al. (2016). Bleeding in patients with atrial fibrillation treated with dabigatran, rivaroxaban or warfarin: A retrospective population-based cohort study. *Eur J Intern Med* 33**,** 55-59. doi: 10.1016/j.ejim.2016.05.023.

Forslund, T., Wettermark, B., Andersen, M., and Hjemdahl, P. (2018). Stroke and bleeding with non-vitamin K antagonist oral anticoagulant or warfarin treatment in patients with non-valvular atrial fibrillation: a population-based cohort study. *Europace* 20(3)**,** 420-428. doi: 10.1093/europace/euw416.

Friberg, L., and Oldgren, J. (2017). Efficacy and safety of non-Vitamin K antagonist oral anticoagulants compared with warfarin in patients with atrial fibrillation. *Open Heart* 4(2). doi: 10.1136/openhrt-2017-000682.

Gieling, E.M., van den Ham, H.A., van Onzenoort, H., Bos, J., Kramers, C., de Boer, A., et al. (2017). Risk of major bleeding and stroke associated with the use of vitamin K antagonists, nonvitamin K antagonist oral anticoagulants and aspirin in patients with atrial fibrillation: a cohort study. *British Journal of Clinical Pharmacology* 83(8)**,** 1844-1859. doi: 10.1111/bcp.13265.

Giugliano, R.P., Ruff, C.T., Braunwald, E., Murphy, S.A., Wiviott, S.D., Halperin, J.L., et al. (2013). Edoxaban versus warfarin in patients with atrial fibrillation. *N Engl J Med* 369(22)**,** 2093-2104. doi: 10.1056/NEJMoa1310907.

Giustozzi, M., Vedovati, M.C., Verso, M., Scrucca, L., Conti, S., Verdecchia, P., et al. (2019). Patients aged 90years or older with atrial fibrillation treated with oral anticoagulants: A multicentre

observational study. *Int J Cardiol* 281, 56-61. doi: 10.1016/j.ijcard.2019.01.071.

Go, A.S., Singer, D.E., Toh, S., Cheetham, T.C., Reichman, M.E., Graham, D.J., et al. (2017). Outcomes of Dabigatran and Warfarin for Atrial Fibrillation in Contemporary Practice: A Retrospective Cohort Study. *Ann Intern Med* 167(12), 845-854. doi: 10.7326/m16-1157.

Graham, D.J., Reichman, M.E., Wernecke, M., Zhang, R., Southworth, M.R., Levenson, M., et al. (2014). Cardiovascular, bleeding, and mortality risks in elderly Medicare patients treated with dabigatran or warfarin for nonvalvular atrial fibrillation. *Circulation* 131(2), 157-164. doi: 10.1161/CIRCULATIONAHA.114.012061.

Granger, C.B., Alexander, J.H., McMurray, J.J., Lopes, R.D., Hylek, E.M., Hanna, M., et al. (2011). Apixaban versus warfarin in patients with atrial fibrillation. *N Engl J Med* 365(11), 981-992. doi: 10.1056/NEJMoa1107039.

Harel, Z., Mamdani, M., Juurlink, D.N., Garg, A.X., Wald, R., Yao, Z., et al. (2016). Novel Oral Anticoagulants and the Risk of Major Hemorrhage in Elderly Patients With Chronic Kidney Disease: A Nested Case-Control Study. *Can J Cardiol* 32(8), 986.e917-922. doi: 10.1016/j.cjca.2016.01.013.

Hernandez, I., Baik, S.H., Pinera, A., and Zhang, Y. (2015). Risk of bleeding with dabigatran in atrial fibrillation. *JAMA Intern Med* 175(1), 18-24. doi: 10.1001/jamainternmed.2014.5398.

Ho, C.W., Ho, M.H., Chan, P.H., Hai, J.J., Cheung, E., Yeung, C.Y., et al. (2015). Ischemic stroke and intracranial hemorrhage with aspirin, dabigatran, and warfarin: impact of quality of anticoagulation control. *Stroke* 46(1), 23-30. doi: 10.1161/STROKEAHA.114.006476.

Hohmann, C., Hohnloser, S.H., Jacob, J., Walker, J., Baldus, S., and Pfister, R. (2019). Non-Vitamin K Oral Anticoagulants in Comparison to Phenprocoumon in Geriatric and Non-Geriatric Patients with Non-Valvular Atrial Fibrillation. *Thromb Haemost*. doi: 10.1055/s-0039-1683422.

Hori, M., Matsumoto, M., Tanahashi, N., Momomura, S., Uchiyama, S., Goto, S., et al. (2012). Rivaroxaban vs. warfarin in Japanese patients with atrial fibrillation - the J-ROCKET AF study. *Circ J* 76(9), 2104-2111.

Hsu, C.C., Hsu, P.F., Sung, S.H., Tu, S.T., Yu, B.H., Huang, C.J., et al. (2018). Is There a Preferred Stroke Prevention Strategy for Diabetic Patients with Non-Valvular Atrial Fibrillation? Comparing Warfarin, Dabigatran and Rivaroxaban. *Thromb Haemost* 118(1), 72-81. doi: 10.1160/th17-02-0095.

Jung, H., Yang, P.S., Jang, E., Yu, H.T., Kim, T.H., Uhm, J.S., et al. (2019). Effectiveness and Safety of Non-Vitamin K Antagonist Oral Anticoagulants in Patients With Atrial Fibrillation With Hypertrophic Cardiomyopathy: A Nationwide Cohort Study. *Chest* 155(2), 354-363. doi: 10.1016/j.chest.2018.11.009.

Kohsaka, S., Katada, J., Saito, K., and Terayama, Y. (2018). Safety and effectiveness of apixaban in comparison to warfarin in patients with nonvalvular atrial fibrillation: a propensity-matched analysis from Japanese administrative claims data. *Curr Med Res Opin*, 1-21. doi: 10.1016/j.jacc.2018.04.001

10.1080/03007995.2018.1478282.

Korenstra, J., Wijtvliet, E.P., Veeger, N.J., Geluk, C.A., Bartels, G.L., Posma, J.L., et al. (2016). Effectiveness and safety of dabigatran versus acenocoumarol in 'real-world' patients with atrial fibrillation. *Europace* 18(9), 1319-1327. doi: 10.1093/europace/euv397.

Koretsune, Y., Yamashita, T., Yasaka, M., Ono, Y., Hirakawa, T., Ishida, K., et al. (2019). Comparative effectiveness and safety of warfarin and dabigatran in patients with non-valvular atrial fibrillation in Japan: A claims database analysis. *J Cardiol* 73(3), 204-209. doi: 10.1016/j.jjcc.2018.09.004.

Kwong, L.M., Turpie, A.G.G., Tamayo, S., Peacock, W.F., Yuan, Z., Sicignano, N., et al. (2017). A

post-marketing assessment of major bleeding in total hip and total knee replacement surgery patients receiving rivaroxaban. *Curr Med Res Opin* 33(9), 1717-1723. doi: 10.1080/03007995.2017.1351935.

Lai, C.L., Chen, H.M., Liao, M.T., and Lin, T.T. (2018). Dabigatran, Rivaroxaban, and Warfarin in the Oldest Adults with Atrial Fibrillation in Taiwan. *J Am Geriatr Soc*. doi: 10.1111/jgs.15430.

Lai, C.L., Chen, H.M., Liao, M.T., Lin, T.T., and Chan, K.A. (2017). Comparative Effectiveness and Safety of Dabigatran and Rivaroxaban in Atrial Fibrillation Patients. *J Am Heart Assoc* 6(4). doi: 10.1161/JAHA.116.005362.

Laliberte, F., Cloutier, M., Nelson, W.W., Coleman, C.I., Pilon, D., Olson, W.H., et al. (2014). Real-world comparative effectiveness and safety of rivaroxaban and warfarin in nonvalvular atrial fibrillation patients. *Curr Med Res Opin* 30(7), 1317-1325. doi: 10.1185/03007995.2014.907140.

Lamberts, M., Staerk, L., Olesen, J.B., Fosbol, E.L., Hansen, M.L., Harboe, L., et al. (2017). Major Bleeding Complications and Persistence With Oral Anticoagulation in Non-Valvular Atrial Fibrillation: Contemporary Findings in Real-Life Danish Patients. *J Am Heart Assoc* 6(2). doi: 10.1161/jaha.116.004517.

Lamsam, L., Sussman, E.S., Iyer, A.K., Bhambhvani, H.P., Han, S.S., Skirboll, S., et al. (2018). Intracranial Hemorrhage in Deep Vein Thrombosis/Pulmonary Embolus Patients Without Atrial Fibrillation: Direct Oral Anticoagulants Versus Warfarin. *Stroke*. doi: 10.1161/strokeaha.118.022156.

Larsen, T.B., Skjoth, F., Nielsen, P.B., Kjaeldgaard, J.N., and Lip, G.Y. (2016). Comparative effectiveness and safety of non-vitamin K antagonist oral anticoagulants and warfarin in patients with atrial fibrillation: propensity weighted nationwide cohort study. *BMJ* 353, i3189. doi: 10.1136/bmj.i3189.

Lau, W.C.Y., Li, X., Wong, I.C.K., Man, K.K.C., Lip, G.Y.H., Leung, W.K., et al. (2017). Bleeding-related hospital admissions and 30-day readmissions in patients with non-valvular atrial fibrillation treated with dabigatran versus warfarin. *J Thromb Haemost* 15(10), 1923-1933. doi: 10.1016/j.tcm.2017.06.012

10.1111/jth.13780.

Lauffenburger, J.C., Farley, J.F., Gehi, A.K., Rhoney, D.H., Brookhart, M.A., and Fang, G. (2015). Effectiveness and safety of dabigatran and warfarin in real-world US patients with non-valvular atrial fibrillation: a retrospective cohort study. *J Am Heart Assoc* 4(4). doi: 10.1161/JAHA.115.001798.

Lee, S.R., Choi, E.K., Han, K.D., Jung, J.H., Oh, S., and Lip, G.Y.H. (2018). Edoxaban in Asian Patients With Atrial Fibrillation: Effectiveness and Safety. *J Am Coll Cardiol* 72(8), 838-853. doi: 10.1016/j.jacc.2018.05.066.

Leschke, M., Hess, S., Weber, E., and Haas, S. (2017). Stroke prevention with rivaroxaban in routine clinical practice - current study data concerning stroke prophylaxis in patients with non-valvular atrial fibrillation in Germany. *Klinikarzt* 46(3), 104-110. doi: 10.1055/s-0043-105184.

Li, W.H., Huang, D., Chiang, C.E., Lau, C.P., Tse, H.F., Chan, E.W., et al. (2017). Efficacy and safety of dabigatran, rivaroxaban, and warfarin for stroke prevention in Chinese patients with atrial fibrillation: the Hong Kong Atrial Fibrillation Project. *Clin Cardiol* 40(4), 222-229. doi: 10.1002/clc.22649.

Lindsay, A., Bengtson, L.G., MacLehose, R.F., Lutsey, P.L., Chen, L.Y., and Lakshminarayan, K. (2014). Intracranial hemorrhage mortality in atrial fibrillation patients treated with dabigatran or warfarin. *Stroke* 45(8), 2286-2291. doi: 10.1161/STROKEAHA.114.006016.

Lip, G.Y., Keshishian, A., Kamble, S., Pan, X., Mardekian, J., Horblyuk, R., et al. (2016). Real-world

comparison of major bleeding risk among non-valvular atrial fibrillation patients initiated on apixaban, dabigatran, rivaroxaban, or warfarin. A propensity score matched analysis. *Thromb Haemost* 116(5)**,** 975-986. doi: 10.1160/TH16-05-0403.

Lip, G.Y.H., Keshishian, A., Li, X., Hamilton, M., Masseria, C., Gupta, K., et al. (2018). Effectiveness and Safety of Oral Anticoagulants Among Nonvalvular Atrial Fibrillation Patients. *Stroke* 49(12)**,** 2933-2944. doi: 10.1161/strokeaha.118.020232.

Martinez, B.K., Sood, N.A., Bunz, T.J., and Coleman, C.I. (2018). Effectiveness and Safety of Apixaban, Dabigatran, and Rivaroxaban Versus Warfarin in Frail Patients With Nonvalvular Atrial Fibrillation. *J Am Heart Assoc* 7(8). doi: 10.1161/jaha.118.008643.

Maura, G., Blotiere, P.O., Bouillon, K., Billionnet, C., Ricordeau, P., Alla, F., et al. (2015). Comparison of the short-term risk of bleeding and arterial thromboembolic events in nonvalvular atrial fibrillation patients newly treated with dabigatran or rivaroxaban versus vitamin K antagonists: a French nationwide propensity-matched cohort study. *Circulation* 132(13)**,** 1252-1260. doi: 10.1161/CIRCULATIONAHA.115.015710.

Meng, S.W., Lin, T.T., Liao, M.T., Chen, H.M., and Lai, C.L. (2019). Direct Comparison of Low-Dose Dabigatran and Rivaroxaban for Effectiveness and Safety in Patients with Non-Valvular Atrial Fibrillation. *Acta Cardiol Sin* 35(1)**,** 42-54. doi: 10.6515/acs.201901_35(1).20180817a.

Moustafa, F., Pesavento, R., di Micco, P., Gonzalez-Martinez, J., Quintavalla, R., Peris, M.L., et al. (2018). Real-life Use of Anticoagulants in Venous Thromboembolism With a Focus on Patients With Exclusion Criteria for Direct Oral Anticoagulants. *Clin Pharmacol Ther* 103(4)**,** 684-691. doi: 10.1002/cpt.781.

Nielsen, P.B., Skjoth, F., Sogaard, M., Kjaeldgaard, J.N., Lip, G.Y., and Larsen, T.B. (2017). Effectiveness and safety of reduced dose non-vitamin K antagonist oral anticoagulants and warfarin in patients with atrial fibrillation: propensity weighted nationwide cohort study. *BMJ* 356**,** j510. doi: 10.1136/bmj.j510.

Norby, F.L., Bengtson, L.G.S., Lutsey, P.L., Chen, L.Y., MacLehose, R.F., Chamberlain, A.M., et al. (2017). Comparative effectiveness of rivaroxaban versus warfarin or dabigatran for the treatment of patients with non-valvular atrial fibrillation. *BMC Cardiovasc Disord* 17(1)**,** 238. doi: 10.1186/s12872-017-0672-5.

Ogawa, S., Shinohara, Y., and Kanmuri, K. (2011). Safety and efficacy of the oral direct factor xa inhibitor apixaban in Japanese patients with non-valvular atrial fibrillation. -The ARISTOTLE-J study. *Circ J* 75(8)**,** 1852-1859.

Okumura, K., Aonuma, K., Kumagai, K., Hirao, K., Inoue, K., Kimura, M., et al. (2016). Efficacy and Safety of Rivaroxaban and Warfarin in the Perioperative Period of Catheter Ablation for Atrial Fibrillation- Outcome Analysis From a Prospective Multicenter Registry Study in Japan. *Circ J* 80(11)**,** 2295-2301. doi: 10.1253/circj.CJ-16-0621.

Palamaner Subash Shantha, G., Bhave, P.D., Girotra, S., Hodgson-Zingman, D., Mazur, A., Giudici, M., et al. (2017). Sex-Specific Comparative Effectiveness of Oral Anticoagulants in Elderly Patients With Newly Diagnosed Atrial Fibrillation. *Circ Cardiovasc Qual Outcomes* 10(4). doi: 10.1161/CIRCOUTCOMES.116.003418.

Patel, M.R., Mahaffey, K.W., Garg, J., Pan, G., Singer, D.E., Hacke, W., et al. (2011). Rivaroxaban versus warfarin in nonvalvular atrial fibrillation. *N Engl J Med* 365(10)**,** 883-891. doi: 10.1056/NEJMoa1009638.

Patti, G., Pecen, L., Lucerna, M., Huber, K., Rohla, M., Renda, G., et al. (2019). Net Clinical Benefit of Non-Vitamin K Antagonist vs Vitamin K Antagonist Anticoagulants in Elderly Patients with Atrial Fibrillation. *Am J Med*. doi: 10.1016/j.pathol.2018.11.008

10.1016/j.amjmed.2018.12.036.

Schafer, J.H., Casey, A.L., Dupre, K.A., and Staubes, B.A. (2018). Safety and Efficacy of Apixaban Versus Warfarin in Patients With Advanced Chronic Kidney Disease. *Ann Pharmacother***,** 1060028018781853. doi: 10.1161/circulationaha.118.034125

10.1177/1060028018781853.

Seeger, J.D., Bykov, K., Bartels, D.B., Huybrechts, K., Zint, K., and Schneeweiss, S. (2015). Safety and effectiveness of dabigatran and warfarin in routine care of patients with atrial fibrillation. *Thromb Haemost* 114(6)**,** 1277-1289. doi: 10.1160/TH15-06-0497.

Shah, S., Norby, F.L., Datta, Y.H., Lutsey, P.L., MacLehose, R.F., Chen, L.Y., et al. (2018). Comparative effectiveness of direct oral anticoagulants and warfarin in patients with cancer and atrial fibrillation. *Blood Adv* 2(3)**,** 200-209. doi: 10.1182/bloodadvances.2017010694.

Siontis, K.C., Zhang, X., Eckard, A., Bhave, N., Schaubel, D.E., He, K., et al. (2018). Outcomes Associated with Apixaban Use in End-Stage Kidney Disease Patients with Atrial Fibrillation in the United States. *Circulation*. doi: 10.1161/circulationaha.118.035418.

Song, X., Gandhi, P., Gilligan, A.M., Arora, P., Wang, C., Henriques, C., et al. (2017). Comparison of All-Cause, Stroke, and Bleed-Specific Healthcare Resource Utilization among Patients with Non-Valvular Atrial Fibrillation (NVAF) and Newly Treated with Dabigatran or Warfarin. *Expert Rev Pharmacoecon Outcomes Res***,** 1-10. doi: 10.1080/14737167.2017.1347041.

Sorensen, R., Gislason, G., Torp-Pedersen, C., Olesen, J.B., Fosbol, E.L., Hvidtfeldt, M.W., et al. (2013). Dabigatran use in Danish atrial fibrillation patients in 2011: a nationwide study. *BMJ Open* 3(5). doi: 10.1136/bmjopen-2013-002758.

Staerk, L., Fosbol, E.L., Lamberts, M., Bonde, A.N., Gadsboll, K., Sindet-Pedersen, C., et al. (2018). Resumption of oral anticoagulation following traumatic injury and risk of stroke and bleeding in patients with atrial fibrillation: a nationwide cohort study. *Eur Heart J* 39(19)**,** 1698-1705a. doi: 10.1093/eurheartj/ehx598.

Staerk, L., Lip, G.Y., Olesen, J.B., Fosbol, E.L., Pallisgaard, J.L., Bonde, A.N., et al. (2015). Stroke and recurrent haemorrhage associated with antithrombotic treatment after gastrointestinal bleeding in patients with atrial fibrillation: nationwide cohort study. *BMJ* 351**,** h5876. doi: 10.1136/bmj.h5876.

Steinberg, B.A., Shrader, P., Pieper, K., Thomas, L., Allen, L.A., Ansell, J., et al. (2018). Frequency and Outcomes of Reduced Dose Non-Vitamin K Antagonist Anticoagulants: Results From ORBIT-AF II (The Outcomes Registry for Better Informed Treatment of Atrial Fibrillation II). *J Am Heart Assoc* 7(4). doi: 10.1161/jaha.117.007633.

Stolk, L.M., de Vries, F., Ebbelaar, C., de Boer, A., Schalekamp, T., Souverein, P., et al. (2017). Risk of myocardial infarction in patients with atrial fibrillation using vitamin K antagonists, aspirin or direct acting oral anticoagulants. *Br J Clin Pharmacol* 83(8)**,** 1835-1843. doi: 10.1111/bcp.13264.

Ujeyl, M., Koster, I., Wille, H., Stammschulte, T., Hein, R., Harder, S., et al. (2018). Comparative risks of bleeding, ischemic stroke and mortality with direct oral anticoagulants versus phenprocoumon in patients with atrial fibrillation. *Eur J Clin Pharmacol*. doi: 10.1017/s0266462318000211

10.1007/s00228-018-2504-7.

van Rein, N., Heide-Jorgensen, U., Lijfering, W.M., Dekkers, O.M., Sorensen, H.T., and Cannegieter, S.C. (2019). Major Bleeding Rates in Atrial Fibrillation Patients on Single, Dual, or Triple Antithrombotic Therapy. *Circulation* 139(6)**,** 775-786. doi: 10.1161/circulationaha.118.036248.

Villines, T.C., Ahmad, A., Petrini, M., Tang, W., Evans, A., Rush, T., et al. (2019). Comparative safety and effectiveness of dabigatran vs. rivaroxaban and apixaban in patients with non-valvular atrial fibrillation: a retrospective study from a large healthcare system. *Eur Heart J*

Cardiovasc Pharmacother 5(2), 80-90. doi: 10.1007/s11606-018-4758-2

10.1093/ehjcvp/pvy044.

Vinogradova, Y., Coupland, C., Hill, T., and Hippisley-Cox, J. (2018). Risks and benefits of direct oral anticoagulants versus warfarin in a real world setting: cohort study in primary care. *BMJ* 362, k2505. doi: 10.1136/bmj.k2505.

Weir, M.R., Berger, J.S., Ashton, V., Laliberte, F., Brown, K., Lefebvre, P., et al. (2017). Impact of renal function on ischemic stroke and major bleeding rates in nonvalvular atrial fibrillation patients treated with warfarin or rivaroxaban: a retrospective cohort study using real-world evidence. *Curr Med Res Opin* 33(10), 1891-1900. doi: 10.1080/03007995.2017.1339674.

Weitz, J.I., Connolly, S.J., Patel, I., Salazar, D., Rohatagi, S., Mendell, J., et al. (2010). Randomised, parallel-group, multicentre, multinational phase 2 study comparing edoxaban, an oral factor Xa inhibitor, with warfarin for stroke prevention in patients with atrial fibrillation. *Thromb Haemost* 104(3), 633-641. doi: 10.1160/th10-01-0066.

Yamashita, T., Koretsune, Y., Yasaka, M., Inoue, H., Kawai, Y., Yamaguchi, T., et al. (2012). Randomized, multicenter, warfarin-controlled phase II study of edoxaban in Japanese patients with non-valvular atrial fibrillation. *Circ J* 76(8), 1840-1847.

Yamashita, Y., Uozumi, R., Hamatani, Y., Esato, M., Chun, Y.H., Tsuji, H., et al. (2017). Current status and outcomes of direct oral anticoagulant use in real-world atrial fibrillation patients — fushimi AF registry —. *Circulation Journal* 81(9), 1278-1285. doi: 10.1253/circj.CJ-16-1337.

Yavuz, B., Ayturk, M., Ozkan, S., Ozturk, M., Topaloglu, C., Aksoy, H., et al. (2016). A real world data of dabigatran etexilate: multicenter registry of oral anticoagulants in nonvalvular atrial fibrillation. *J Thromb Thrombolysis* 42(3), 399-404. doi: 10.1007/s11239-016-1361-4.

Yoshimura, S., Koga, M., Sato, S., Todo, K., Yamagami, H., Kumamoto, M., et al. (2018). Two-Year Outcomes of Anticoagulation for Acute Ischemic Stroke With Nonvalvular Atrial Fibrillation-SAMURAI-NVAF Study. *Circ J* 82(7), 1935-1942. doi: 10.1253/circj.CJ-18-0067.

Zoppellaro, G., Zanella, L., Denas, G., Gennaro, N., Ferroni, E., Fedeli, U., et al. (2018). Different safety profiles of oral anticoagulants in very elderly non-valvular atrial fibrillation patients. A retrospective propensity score matched cohort study. *Int J Cardiol*. doi: 10.1016/j.ijcard.2018.04.117.
